# Supplementary material for: Iron phosphide nanocrystals as an air-stable heterogeneous catalyst for liquid-phase nitrile hydrogenation
Source: Nat Commun. 2023 Sep 28;14:5959. doi: 10.1038/s41467-023-41627-6 (PMC10539298; doi:10.1038/s41467-023-41627-6)
Supplement: Supplementary file 1 — Supplementary Information [file 41467_2023_41627_MOESM1_ESM.pdf]

Supplementary Information

Iron phosphide nanocrystals as an air-stable heterogeneous catalyst for liquid-phase nitrile hydrogenation

Table of Contents

1. Characterization and reaction studies

**Supplementary Fig. 1**	XRD pattern of the prepared Fe_2P NCs.
**Supplementary Fig. 2**	Size distribution histograms of Fe_2P NCs.
**Supplementary Fig. 3**	EDX analysis of Fe_2P NCs.
**Supplementary Fig. 4**	Structural characterization of Fe_2P NC/TiO_2.
**Supplementary Fig. 5**	EELS spectrum of the Fe $L_{2,3}$ edge in Fe_2P NCs.
**Supplementary Fig. 6**	P 2*p* XPS spectrum of Fe_2P NCs.
**Supplementary Fig. 7**	Time course of the hydrogenation of **1a** using Fe_2P NC/TiO_2 in the presence or absence of NH_3.
**Supplementary Fig. 8**	Dependency of NH_3 partial pressure in hydrogenation of **1a**.
**Supplementary Fig. 9**	A plausible reaction pathway for the hydrogenation of **1a** to **2a**.
**Supplementary Fig. 10**	Hydrogenation of expected imine intermediates using Fe_2P NC/TiO_2.
**Supplementary Fig. 11**	^{1}H NMR spectrum of **2a**-hydrochloride synthesized from the hydrogenation of **1a** using 2-propanol or 2-propanol-d_8 as solvent.
**Supplementary Fig. 12**	Hydrogenation of **1a** under argon atmosphere.
**Supplementary Fig. 13**	Reuse experiments.
**Supplementary Fig. 14**	TEM images of supported Fe_2P NCs before and after the reaction.
**Supplementary Fig. 15**	Fe 2*p* XPS spectra of Fe_2P NC/TiO_2 before and after the reaction.
**Supplementary Fig. 16**	EXAFS fitting curves in *k*-space and *R*-space of Fe foil, bulk Fe_2P, Fe_2P NCs, and Fe_2P NC/TiO_2.
**Supplementary Table 1**	Hydrogenation of **1a** to **2a** using various Fe_2P NC catalysts.
**Supplementary Table 2**	Comparison of activity between Fe_2P NC/TiO_2 and reported transition metal (iron, nickel, and cobalt) based heterogeneous catalysts for hydrogenation of **1a**.
**Supplementary Table 3**	ICP-AES elemental analyses of Fe_2P NCs and Fe_2P NC/TiO_2.
**Supplementary Table 4**	Curve-fitting results of Fe *K*-edge EXAFS for Fe foil, bulk Fe_2P, Fe_2P NCs, and Fe_2P NC/TiO_2.

2. Product identification

3. ^{1}H and ^{13}C NMR spectra

4. Supplementary discussion

5. Supplementary References

1. Characterization and reaction studies

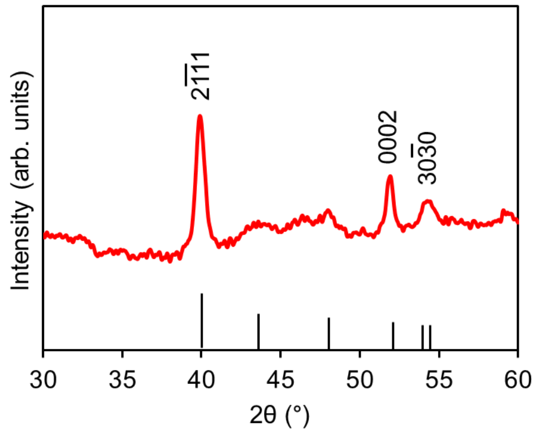

Supplementary Fig. 1|XRD pattern of the prepared Fe_2P NCs. Black bars below the pattern show the diffraction peaks referring to JCPDS card number 51-0943.

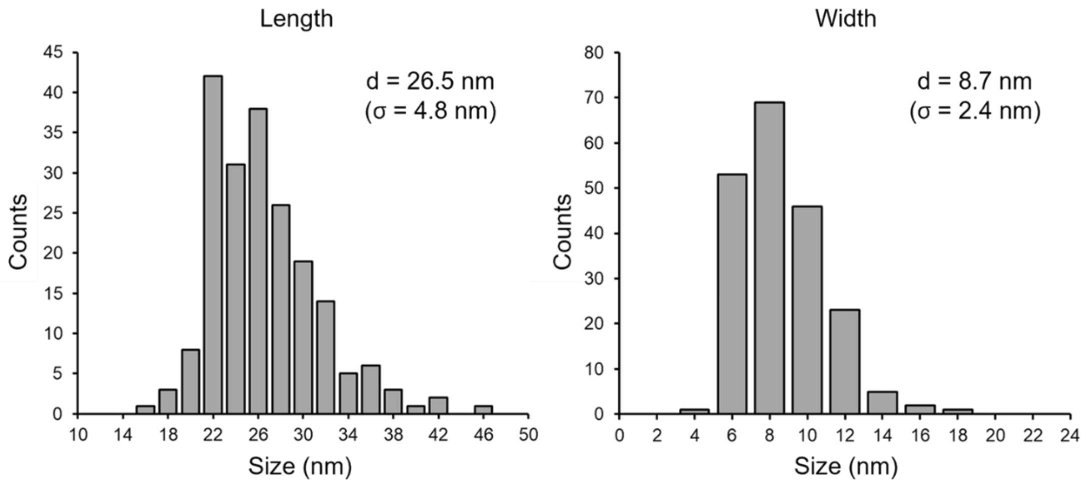

Supplementary Fig. 2|Size distribution histograms of Fe_2P NCs. (left) Length and (right) width of Fe_2P NCs.

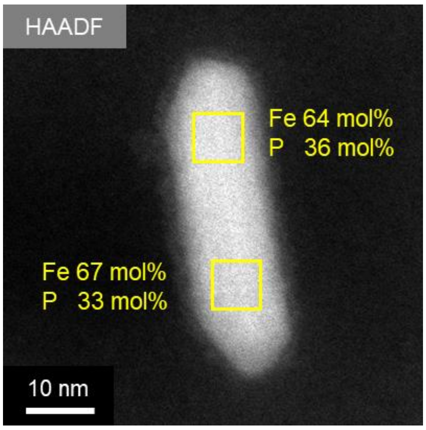

Supplementary Fig. 3|EDX analysis of Fe_2P NCs. The yellow squares indicate the analysis areas.

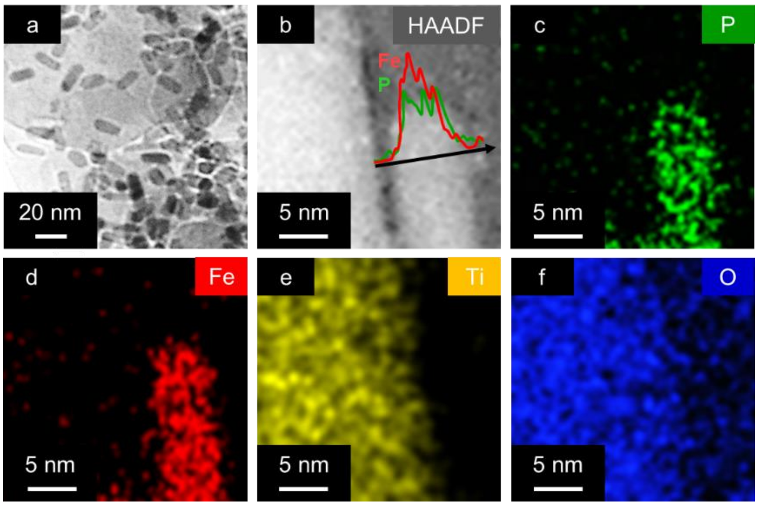

Supplementary Fig. 4|Structural characterization of Fe_2P NC/TiO_2. a, TEM image of Fe_2P NC/TiO_2. **b**, HAADF-STEM image of Fe_2P NC/TiO_2 with line scan analysis and elemental mapping images of **c**, P, **d**, Fe, **e**, Ti, and **f**, O.

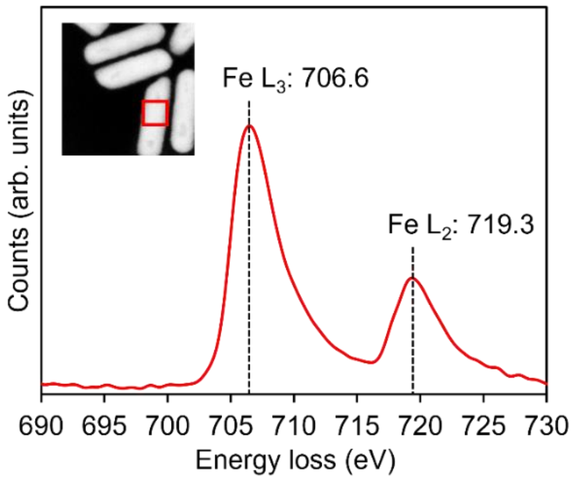

Supplementary Fig. 5|EELS spectrum of the Fe $L_{2,3}$ edge in Fe_2P NCs. The red square in the inset HAADF-STEM image indicates the analysis area.

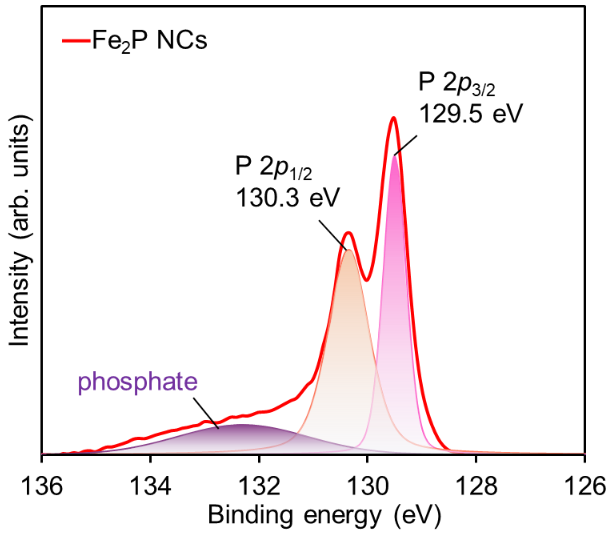

Supplementary Fig. 6|P 2*p* XPS spectrum of Fe_2P NCs. Pink and orange areas show the asymmetric split of two P^0 peaks ($2p_{3/2}$ and $2p_{1/2}$). Purple area shows the phosphate species.

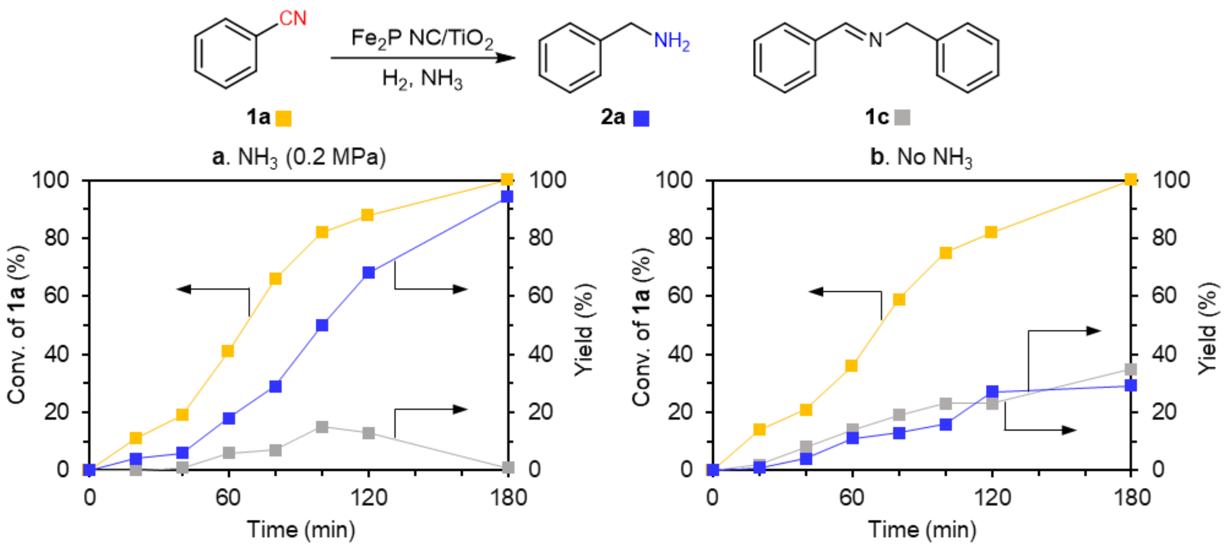

Supplementary Fig. 7|Time course of the hydrogenation of 1a using Fe_2P NC/TiO_2 (a) in the presence or (b) absence of NH_3. Reaction conditions: Fe_2P NC/TiO_2 (0.1 g, Fe: 7.6 mol%), **1a** (0.5 mmol), 2-propanol (3 mL), H_2 (3.8 MPa), 453 K. Conversion and yield were determined by gas chromatography (GC) using an internal standard technique. We confirmed the influence of NH_3 on the selectivity of **2a** in the hydrogenation of **1a**. In the absence of NH_3, the selectivity of **2a** considerably decreased due to the formation of the *N*-benzylidenebenzylamine (**1c**), which is produced by the attack of **2a** on the primary imine intermediate, resulting in the concurrent release of NH_3. Therefore, the addition of NH_3 is effective to suppress the deammoniation process, thereby enhancing the selectivity of **2a**.

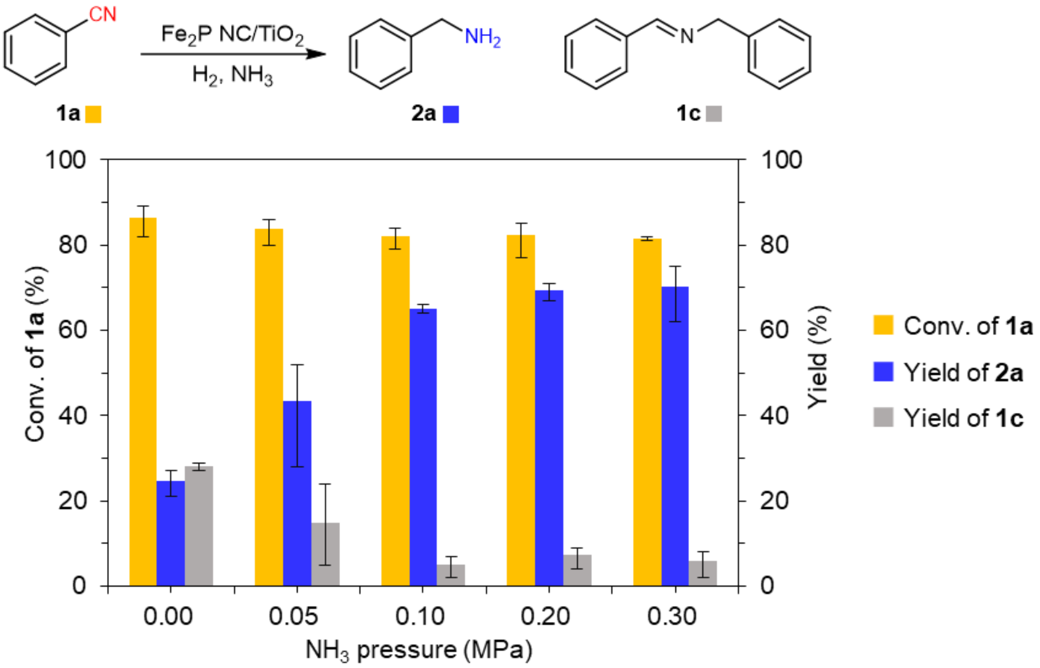

Supplementary Fig. 8|Dependency of NH_3 partial pressure in hydrogenation of 1a. Reaction conditions: Fe_2P NC/TiO_2 (0.1 g, Fe: 7.6 mol%), **1a** (0.5 mmol), 2-propanol (3 mL), H_2 (3.8 MPa), 453 K, 2 h. The columns denote the data mean values, and the error bars show the range. We investigated the influence of NH_3 pressure on the yield of the primary amine product in the hydrogenation of **1a**. The result demonstrated that the increase of NH_3 pressure enhanced the yield of the primary amine product. However, beyond an NH_3 injection pressure of 0.1 MPa, there was no further improvement in the amine yield.

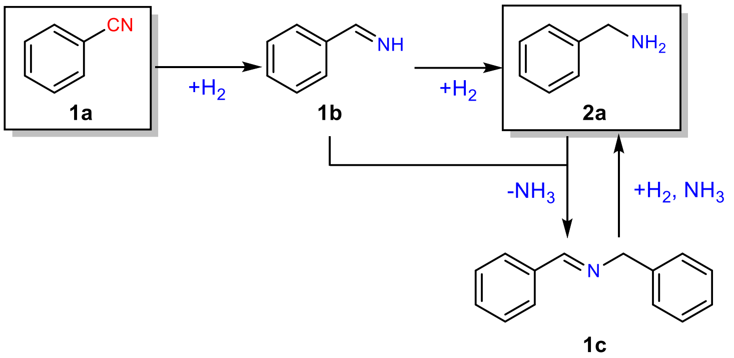

Supplementary Fig. 9|A plausible reaction pathway for the hydrogenation of 1a to 2a. The hydrogenation of **1a** produces benzylideneamine (**1b**), which is then hydrogenated to **2a**. Subsequently, **1c** is generated through the condensation of **1b** and **2a**, followed by its decomposition to produce **2a**.

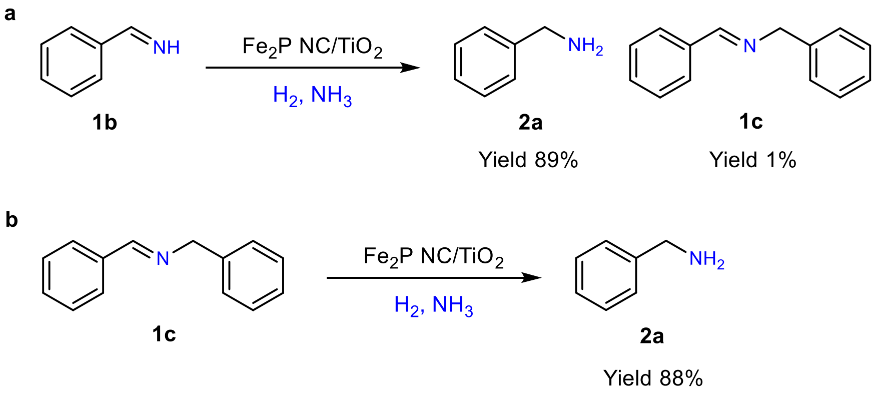

Supplementary Fig. 10|Hydrogenation of expected imine intermediates using Fe_2P NC/TiO_2. a, Hydrogenation of **1b**. Reaction conditions: Fe_2P NC/TiO_2 (0.1 g, Fe: 7.6 mol%), **1b** (0.5 mmol), 2-propanol (3 mL), H_2 (3.8 MPa), NH_3 (0.2 MPa), 453 K, 3 h. **b**, Hydrogenation of **1c**. Reaction conditions: Fe_2P NC/TiO_2 (0.1 g, Fe: 7.6 mol%), **1c** (0.5 mmol), 2-propanol (3 mL), H_2 (3.8 MPa), NH_3 (0.2 MPa), 453 K, 3 h. Fe_2P NC/TiO_2 facilitated the hydrogenation of **1b** to give desired product **2a** in 89% along with the production of **1c**. Furthermore, **1c** was efficiently transformed to **2a** with high yield.

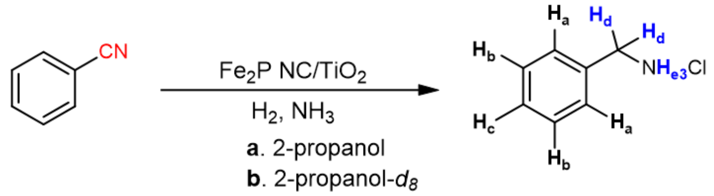

a. 2-propanol as solvent

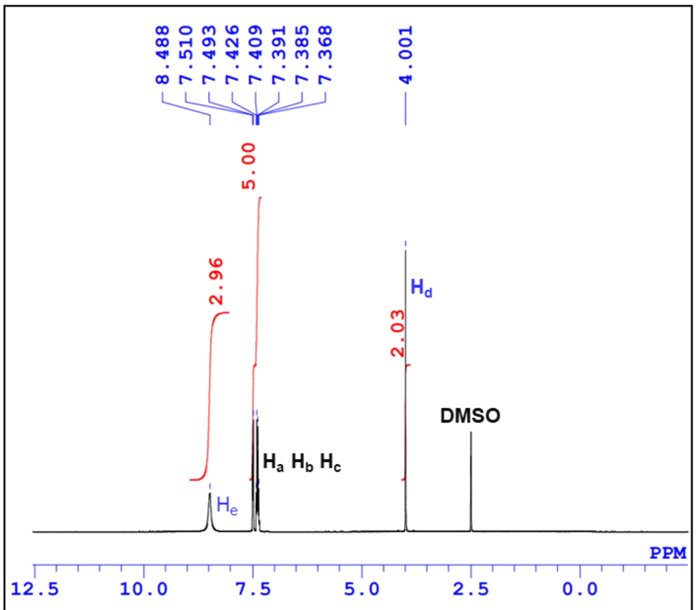

proton	integration	
	theoretical	experimental
$H_a + H_b + H_c$	5.00	5.00
H_d	2.00	2.03
H_e	3.00	2.96

b. 2-propanol-d_8 as solvent

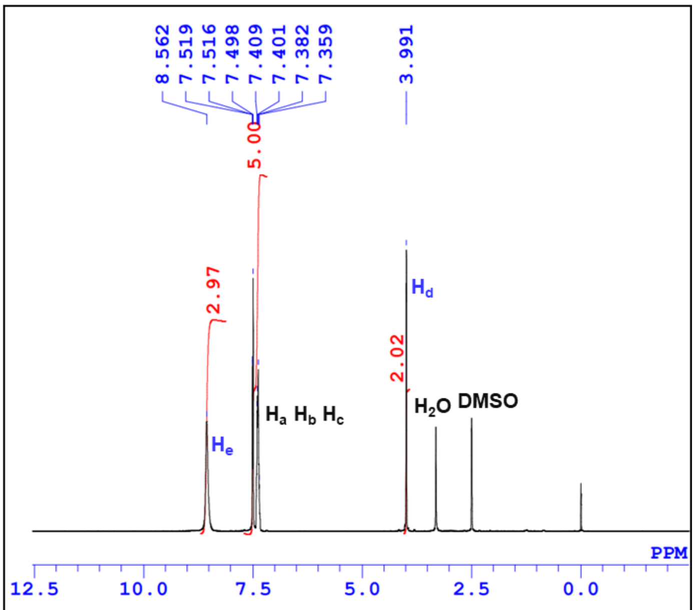

proton	integration	
	theoretical	experimental
$H_a + H_b + H_c$	5.00	5.00
H_d	2.00	2.02
H_e	3.00	2.97

Supplementary Fig. 11|^{1}H NMR spectrum of 2a-hydrochloride synthesized from the hydrogenation of 1a using (a) 2-propanol or (b) 2-propanol-*d₈* as solvent. We conducted the hydrogenation of benzonitrile using deuterium-labeled 2-propanol (2-propanol-d_8) as a solvent. After the reaction, we analyzed the hydrochloride salt of the product using ^{1}H NMR spectroscopy. It was observed that the resulting amine product did not show any deuterium incorporation.

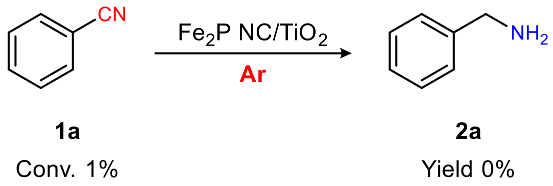

Supplementary Fig. 12|Hydrogenation of 1a under argon atmosphere. Reaction conditions: Fe_2P NC/TiO_2 (0.1 g, Fe: 7.6 mol%), **1a** (0.5 mmol), 2-propanol (3 mL), Ar (1.0 MPa), 453 K, 3 h. Fe_2P NC/TiO_2 did not promote the hydrogenation of **1a** under argon atmosphere. This result clearly shows that 2-propanol does not act as a hydrogen source in this reaction.

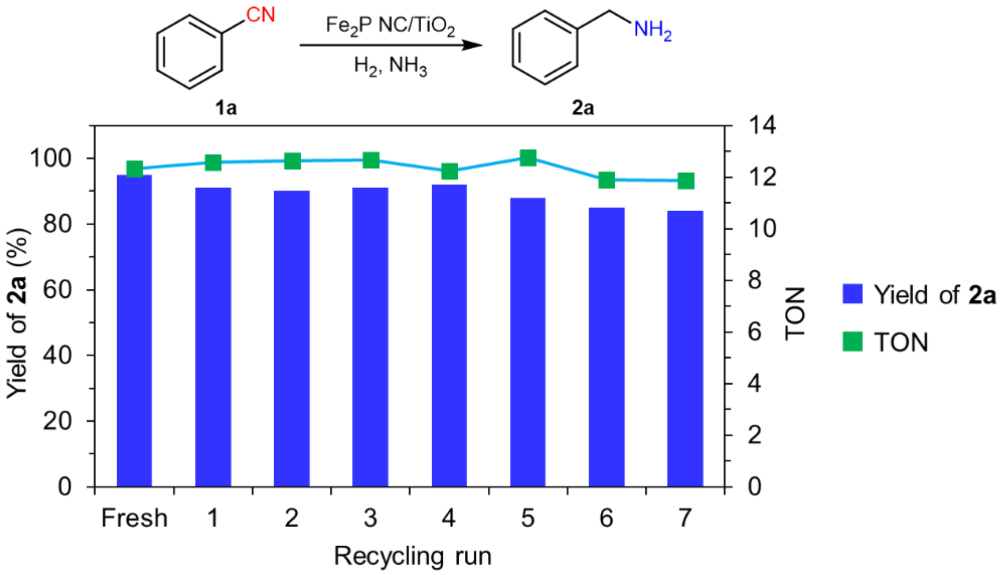

Supplementary Fig. 13|Reuse experiments. Reaction conditions: Fe_2P NC/TiO_2 (0.1 g), **1a** (0.5 mmol), 2-propanol (3 mL), H_2 (3.8 MPa), NH_3 (0.2 MPa), 453 K, 3 h. To investigate the underlying cause of slight decrease in the yield of **2a**, we calculated the turnover number (TON) based on the amount of iron in the recovered Fe_2P/TiO_2 catalyst. Despite the observed decrease in yield of **2a** (indicated by blue bars), the TON values for each cycle remained relatively consistent (represented by green squares). These results provide further evidence that the decrease in yield primarily arises from the minor loss of catalyst during the recovery process.

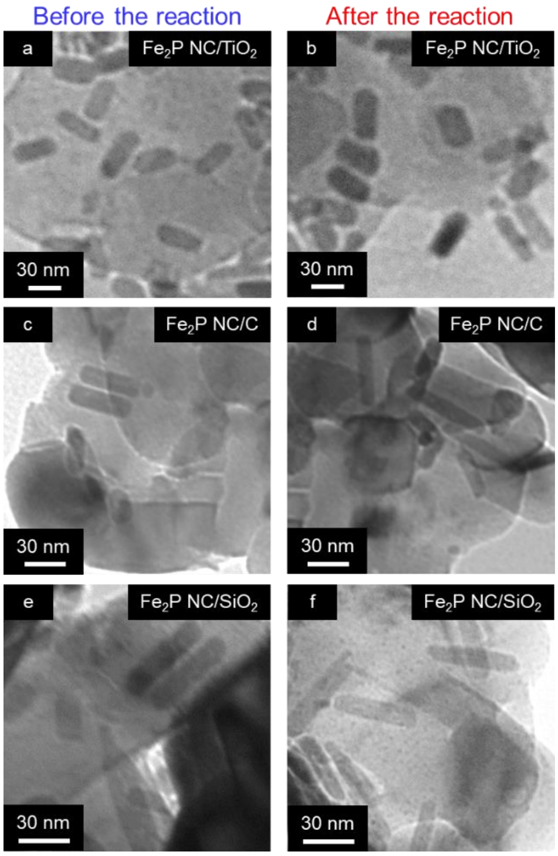

Supplementary Fig. 14|TEM images of supported Fe_2P NCs before and after the reaction. (**a**, **b**) Fe_2P NC/TiO_2, (**c**, **d**) Fe_2P NC/C, and (**e**, **f**) Fe_2P NC/SiO_2 catalysts before (left panels) and after (right panels) the reaction.

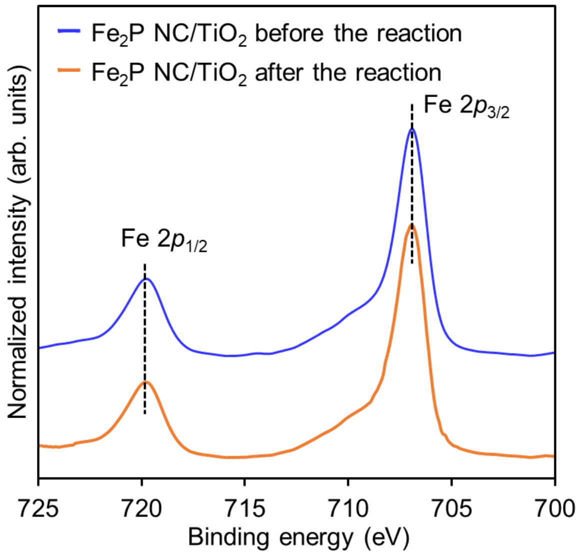

Supplementary Fig. 15|Fe 2*p* XPS spectra of Fe_2P NC/TiO_2 before and after the reaction. Fe 2*p* XPS spectra (blue) before the reaction, and (orange) after the reaction.

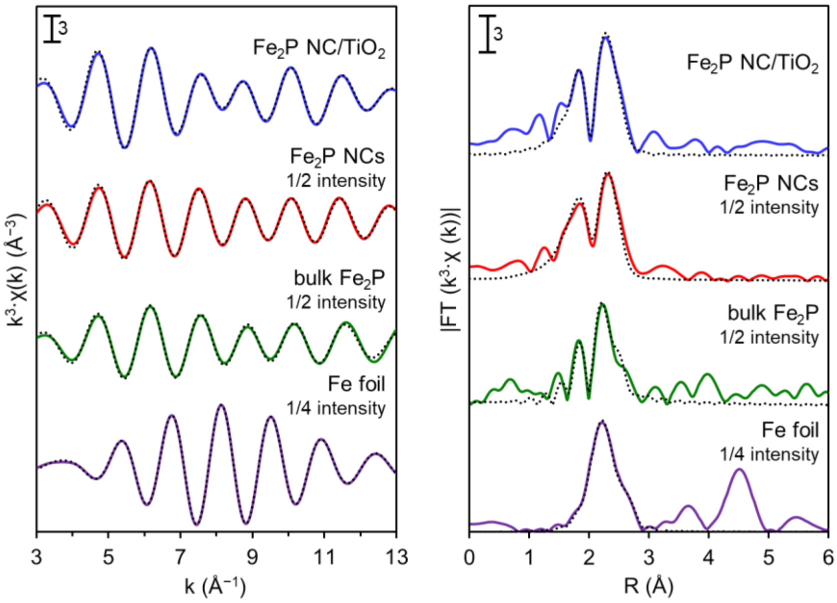

Supplementary Fig. 16|EXAFS fitting curves in *k*-space and *R*-space of Fe foil, bulk Fe_2P, Fe_2P NCs, and Fe_2P NC/TiO_2. EXAFS fitting curves in (left) *k*-space and (right) *R*-space of (purple) Fe foil, (green) bulk Fe_2P, (red) Fe_2P NCs, and (blue) Fe_2P NC/TiO_2. The solid lines are the obtained spectra. The dashed lines show the fitting curves.

Supplementary Table 1|Hydrogenation of 1a to 2a using various Fe_2P NC catalysts.

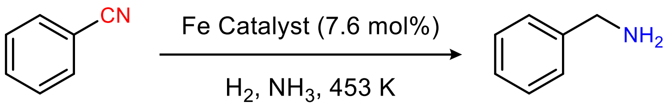

1a **2a**

Entry	Catalyst	Conv. of **1a** (%)	Yield of **2a** (%)
1	Fe_2P NCs	36	20
2	Fe_2P NC/TiO_2	89	78
3	Fe_2P NC/C	45	28
4	Fe_2P NC/ZrO_2	34	26
5	Fe_2P NC/CeO_2	41	24
6	Fe_2P NC/SiO_2	33	17
7	Fe_2P NC/Fe_2O_3	31	17

Reaction conditions: **1a** (0.5 mmol), 2-propanol (3 mL), H_2 (3.8 MPa), NH_3 (0.2 MPa), 2 h. Conversion and yield were determined by GC using the internal standard technique.

Supplementary Table 2|Comparison of activity between Fe_2P NC/TiO_2 and reported transition metal (iron, nickel, and cobalt) based heterogeneous catalysts for hydrogenation of 1a.

Active metal	Catalyst	Reaction conditions	TON	Ref.
**Fe**	Fe_2P NC/TiO_2	7.6 mol% Fe, 3.8 MPa H_2, 0.2 MPa NH_3, 453 K, 3 h.	13	**This work**
	Fe_2P NC/TiO_2	0.33 mol% Fe, 4.4 MPa H_2, 0.6 MPa NH_3, 453 K, 24 h.	**295**	**This work**
	Fe/Fe–O@SiO_2	8.5 mol% Fe, 20 mol% Al foil, 5 MPa H_2, 0.5–0.7 MPa NH_3, 393 K, 24 h.	11	[S1]
**Ni**	nano-Ni_2P	5 mol% Ni, NH_3 aq., 4 MPa H_2, 403 K, 3 h.	19	[S2]
	MC/Ni	13 mol% Ni, NH_3 aq. (36 wt. %), 0.25 MPa H_2, 353 K, 6 h.	8	[S3]
	Ni nanoparticles	0.7 mol% Ni, 2.5 MPa H_2, 363 K, 22 h.	114	[S4]
	Ni/Al_2O_3-600	12 mol% Ni, NH_3 aq. (36.5 wt%), 0.25 MPa H_2, 333 K, 6 h.	8	[S5]
	Ni-phen@SiO_2	4.5 mol% Ni, 7 M NH_3/MeOH, 5 MPa H_2, 373 K, 20 h.	20	[S6]
	$NiMg_{0.75}Al_{0.25}O$-op	12 mol% Ni, 4 MPa H_2, 373 K, 2 h.	8	[S7]
**Co**	nano-Co_2P/HT	4.2 mol% Co, NH_3 aq., 4 MPa H_2, 403 K, 1 h.	22	[S8]
	$Co(acac)_3$ + Tetradentate phosphine	4 mol% Co, 4.4 mol% tetradentate phosphine, 10 mol% KO^{t}Bu, 3 MPa H_2, 353 K, 18 h.	25	[S9]
	Zr_{12}-TPDC-Co	0.5 mol% Co, 1 M $NaBEt_3H$/THF, 4 MPa H_2, 383 K, 42 h.	200	[S10]
	$Co(OAc)_2$/Phen@α-Al_2O_3	4 mol% Co, NH_3 aq., 4 MPa H_2, 353 K, 2 h.	25	[S11]
	Cobalt-terephthalic acid MOF@C-800	3.8 mol% Co, 2 MPa H_2, 0.5 MPa NH_3, 393 K, 20 h.	25	[S12]
	Co_3O_4/NGr@CeO_2	1.6 mol% Co, NH_3 aq., 3 MPa H_2, 393 K, 15 h.	60	[S13]
	Co-N-C@MgO-700	1 mol% Co, 2 MPa H_2, NH_3 aq., 353 K, 24 h.	98	[S14]
	Co_2P NR/HT	2.5 mol% Co, NH_3 aq., 4 MPa H_2, 403 K, 4 h.	37	[S15]

Supplementary Table 3|ICP-AES elemental analyses of Fe_2P NCs and Fe_2P NC/TiO_2.

Sample	Element	Amount (wt%)	Fe/P (molar ratio)
Fe_2P NCs	Fe	76.6	2.09
	P	20.4	
Fe_2P NC/TiO_2 before reaction	Fe	2.30	1.62
	P	0.79	
Fe_2P NC/TiO_2 after reaction	Fe	2.11	1.56
	P	0.75	

Supplementary Table 4|Curve-fitting results of Fe *K*-edge EXAFS for Fe foil, bulk Fe_2P, Fe_2P NCs, and Fe_2P NC/TiO_2.

Sample	Shell	CN^a	r (Å)^b	D.W.^{c}	R factor (%)
Fe foil	Fe–Fe	7.3 ± 0.2	2.48 ± 0.02	0.006 ± 0.002	2.4
		4.5 ± 0.2	2.86 ± 0.03	0.007 ± 0.003	
bulk Fe_2P	Fe–P	0.5 ± 0.1	2.24 ± 0.04	0.001 ± 0.001	10.5
	Fe–Fe	1.8 ± 0.1	2.56 ± 0.03	0.003 ± 0.002	
	Fe–Fe	0.8 ± 0.2	2.69 ± 0.03	0.001 ± 0.001	
Fe_2P NCs	Fe–P	2.2 ± 0.2	2.23 ± 0.04	0.006 ± 0.004	5.0
	Fe–Fe	3.7 ± 0.3	2.65 ± 0.03	0.011 ± 0.005	
Fe_2P NC/TiO_2	Fe–P	1.4 ± 0.2	2.22 ± 0.04	0.003 ± 0.002	6.1
	Fe–Fe	4.2 ± 0.2	2.63 ± 0.03	0.010 ± 0.004	

^{a}Coordination number. ^{b}Bond distance. ^{c}Debye–Waller factor.

2. Product identification

All reaction products were characterized by GC and NMR spectroscopy. The ^{1}H and ^{13}C NMR chemical shifts of the products agreed with those of authentic samples or previously reported values.

benzylamine hydrochloride (**2a**) [S11]

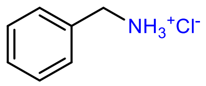

^{1}H NMR (DMSO-*d*$_6$, 400 MHz): δ = 8.49 (br s, 3H), 7.50 (d, *J* = 7.2 Hz, 2H), 7.43–7.35 (m, 3H), 4.00 (s, 2H); ^{13}C NMR (DMSO-*d*$_6$, 100 MHz): δ = 134.07, 128.88, 128.51, 128.35, 42.12.

o-methylbenzylamine hydrochloride (**2b**) [S16]

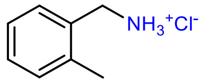

^{1}H NMR (DMSO-*d*$_6$, 400 MHz): δ = 8.59 (br s, 3H), 7.46–7.43 (m, 1H), 7.33–7.21 (m, 3H), 3.98 (s, 2H), 2.36 (s, 3H); ^{13}C NMR (DMSO-*d*$_6$, 100 MHz): δ = 136.64, 132.33, 130.26, 129.20, 128.41, 126.03, 39.39, 18.83.

m-methylbenzylamine hydrochloride (**2c**) [S16]

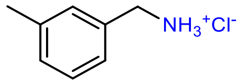

^{1}H NMR (DMSO-*d*$_6$, 400 MHz): δ = 8.45 (s, 3H), 7.32–7.28 (m, 3H), 7.20–7.18 (m, 1H), 3.96 (s, 2H), 2.32 (s, 3H); ^{13}C NMR (DMSO-*d*$_6$, 100 MHz): δ = 137.68, 133.98, 129.45, 128.94, 128.47, 125.91, 42.07, 20.92.

p-methylbenzylamine hydrochloride (**2d**) [S16]

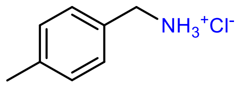

^{1}H NMR (DMSO-*d*$_6$, 400 MHz): δ = 8.38 (s, 3H), 7.37 (d, *J* = 8.4 Hz, 2H), 7.22 (d, *J* = 8.4 Hz, 2H), 3.95 (s, 2H), 2.30 (s, 3H); ^{13}C NMR (DMSO-*d*$_6$, 100 MHz): δ = 137.64, 131.00, 128.97, 128.85, 41.80, 20.67.

p-*tert*-butylbenzylamine hydrochloride (**2e**) [S17]

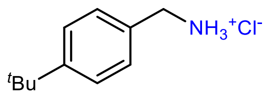

^{1}H NMR (DMSO-d_6, 400 MHz): δ = 8.36 (s, 3H), 7.45–7.40 (m, 4H), 3.96 (s, 2H), 1.28 (s, 9H); ^{13}C NMR (DMSO-d_6, 100 MHz): δ = 150.95, 131.16, 128.68, 125.29, 41.82, 34.30, 31.02.

o-methoxybenzylamine hydrochloride (**2f**) [S11]

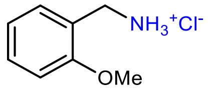

^{1}H NMR (DMSO-d_6, 400 MHz): δ = 8.17 (br s, 3H), 7.40–7.36 (m, 2H), 7.07–7.06 (m, 1H), 6.99–6.96 (m, 1H), 3.95 (q, *J* = 6.0 Hz, 2H), 3.83 (s, 3H); ^{13}C NMR (DMSO-d_6, 100 MHz): δ = 157.20, 130.30, 130.25, 121.60, 120.30, 110.93, 55.51, 37.69.

m-methoxybenzylamine hydrochloride (**2g**) [S17]

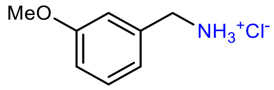

^{1}H NMR (DMSO-d_6, 400 MHz): δ = 8.62 (br s, 3H), 7.31 (t, *J* = 8.0 Hz, 1H), 7.19 (s, 1H), 7.06 (d, *J* = 8.0 Hz, 1H), 6.94–6.92 (m, 1H), 3.97 (s, 2H), 3.77 (s, 3H); ^{13}C NMR (DMSO-d_6, 100 MHz): δ = 159.29, 135.56, 129.62, 120.95, 114.48, 113.92, 55.19, 42.05.

p-methoxybenzylamine hydrochloride (**2h**) [S18]

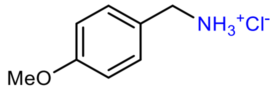

^{1}H NMR (DMSO-d_6, 400 MHz): δ = 8.40 (s, 3H), 7.43 (d, *J* = 8.4 Hz, 2H), 6.96 (d, *J* = 8.4 Hz, 2H), 3.93 (s, 2H), 3.79 (s, 3H); ^{13}C NMR (DMSO-d_6, 100 MHz): δ = 159.22, 130.40, 125.86, 113.79, 55.07, 41.51.

o-chlorobenzylamine hydrochloride (**2i**) [S11]

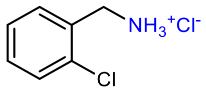

^{1}H NMR (DMSO-d_6, 400 MHz): δ = 8.66 (br s, 3H), 7.68–7.63 (m, 1H), 7.56–7.52 (m, 1H), 7.45–7.41 (m, 2H), 4.12 (s, 2H); ^{13}C NMR (DMSO-d_6, 100 MHz): δ = 132.86, 131.62, 130.65, 130.34, 129.45, 127.49, 39.43.

m-chlorobenzylamine hydrochloride (**2j**) [S16]

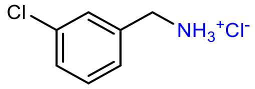

^{1}H NMR (DMSO-d_6, 400 MHz): δ = 8.63 (br s, 3H), 7.65 (s, 1H), 7.50–7.43 (m, 3H), 4.03 (s, 2H); ^{13}C NMR (DMSO-d_6, 100 MHz): δ = 136.52, 133.04, 130.41, 128.91, 128.31, 127.75, 41.46.

p-chlorobenzylamine hydrochloride (**2k**) [S19]

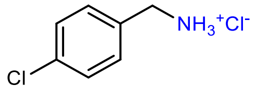

^{1}H NMR (DMSO-d_6, 400 MHz): δ = 8.55 (br s, 3H), 7.58–7.47 (m, 4H), 4.01 (s, 2H); ^{13}C NMR (DMSO-d_6, 100 MHz): δ = 133.11, 133.08, 130.95, 128.47, 41.35.

p-bromobenzylamine hydrochloride (**2l**) [S19]

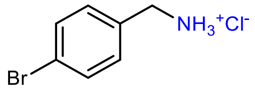

^{1}H NMR (DMSO-d_6, 400 MHz): δ = 8.53 (br s, 3H), 7.63 (d, J = 8.0 Hz, 2H), 7.46 (d, J = 8.0 Hz, 2H), 4.00 (s, 2H); ^{13}C NMR (DMSO-d_6, 100 MHz): δ = 133.53, 131.39, 131.24, 121.66, 41.42.

p-fluorobenzylamine hydrochloride (**2m**) [S11, S20]

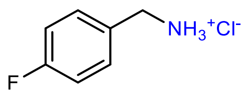

^{1}H NMR (DMSO-d_6, 400 MHz): δ = 8.47 (br s, 3H), 7.59–7.54 (m, 2H), 7.28–7.23 (m, 2H), 4.01 (q, *J* = 6.0 Hz, 2H); ^{13}C NMR (DMSO-d_6, 100 MHz): δ = 162.07 (d, *J* = 244.0 Hz), 131.34 (d, *J* = 8.6 Hz), 130.36 (d, *J* = 2.9 Hz), 115.35 (d, *J* = 21.9 Hz), 41.38.

2,4-difluorobenzylamine hydrochloride (**2n**) [S21]

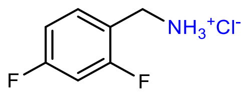

^{1}H NMR (D_2O, 400 MHz): δ = 7.46–7.40 (m, 1H), 7.04–6.97 (m, 2H), 4.17 (s, 2H); ^{13}C NMR (D_2O, 100 MHz): δ = 165.05 (dd, *J* = 246.9, 12.4 Hz), 162.75 (dd, *J* = 247.9, 12.4 Hz), 134.08 (dd, *J* = 10.4, 4.7 Hz), 117.40 (dd, *J* = 15.2, 3.8 Hz), 113.65 (dd, *J* = 22.0, 3.8 Hz), 105.84 (dd, *J* = 25.7, 25.7 Hz), 38.19 (d, *J* = 3.8 Hz).

3,4-dichlorobenzylamine hydrochloride (**2o**) [S19]

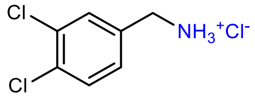

^{1}H NMR (CD_3OD, 400 MHz): δ = 7.70 (s, 1H), 7.64 (d, *J* = 8.4 Hz, 1H), 7.43 (d, *J* = 8.4 Hz, 1H), 4.15 (s, 2H); ^{13}C NMR (CD_3OD, 100 MHz): δ = 135.02, 134.31, 133.97, 132.32, 132.29, 130.00, 43.14.

p-(trifluoromethyl)benzylamine hydrochloride (**2p**) [S18]

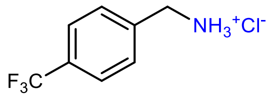

^{1}H NMR (DMSO-*d*$_6$, 400 MHz): δ = 8.48 (s, 3H), 7.81 (d, *J* = 8.0 Hz, 2H), 7.72 (d, *J* = 8.0 Hz, 2H), 4.14 (s, 2H); ^{13}C NMR (DMSO-*d*$_6$, 100 MHz): δ = 138.76, 129.71, 128.62 (q, *J* = 31.5 Hz), 125.42 (q, *J* = 3.8 Hz), 122.76, 41.64.

p-aminobenzylamine hydrochloride (**2q**) [S22, S23]

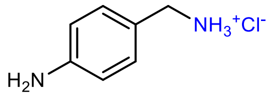

^{1}H NMR (DMSO-*d*$_6$, 400 MHz): δ = 8.46 (br s, 3H), 7.53 (d, *J* = 8.4 Hz, 2H), 7.30 (d, *J* = 8.4 Hz, 2H), 4.00 (q, *J* = 6.0 Hz, 2H); ^{13}C NMR (DMSO-*d*$_6$, 100 MHz): δ = 134.70, 131.84, 130.31, 121.90, 41.63.

p-(*N*,*N*-dimethylamino)benzylamine hydrochloride (**2r**) [S17]

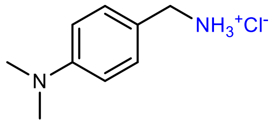

^{1}H NMR (DMSO-*d*$_6$, 400 MHz): δ = 8.29 (br s, 3H), 7.36 (d, *J* = 8.0 Hz, 2H), 6.95 (br s, 2H), 3.90 (q, *J* = 5.2 Hz, 2H), 2.94 (s, 6H); ^{13}C NMR (DMSO-*d*$_6$, 100 MHz): δ = 148.01, 130.22, 115.32, 41.94, 41.70.

p-(methylthio)benzylamine hydrochloride (**2s**) [S1]

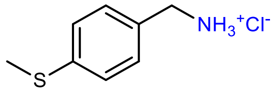

^{1}H NMR (DMSO-*d*$_6$, 400 MHz): δ = 8.32 (br s, 3H), 7.42 (d, *J* = 8.4 Hz, 2H), 7.30 (d, *J* = 8.4 Hz, 2H), 3.97 (s, 2H), 2.51–2.50 (m, 3H); ^{13}C NMR (DMSO-*d*$_6$, 100 MHz): δ = 138.72, 130.40, 129.58, 125.83, 41.75, 14.59.

p-phenoxybenzylamine hydrochloride (**2t**) [S17]

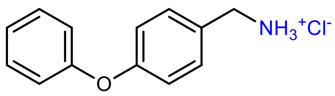

^{1}H NMR (DMSO-d_6, 400 MHz): δ = 8.43 (s, 3H), 7.52 (d, *J* = 8.0 Hz, 2H), 7.43–7.39 (m, 2H), 7.19–7.15 (m, 1H), 7.05–7.00 (m, 4H), 3.99 (s, 2H); ^{13}C NMR (DMSO-d_6, 100 MHz): δ = 156.83, 156.34, 130.97, 130.11, 129.04, 123.69, 118.72, 118.49, 41.56.

3,4-methylenedioxybenzylamine hydrochloride (**2u**) [S17]

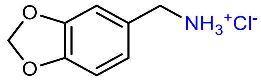

^{1}H NMR (DMSO-d_6, 400 MHz): δ = 8.29 (br s, 3H), 7.09 (s, 1H), 6.97–6.93 (m, 2H), 6.04 (s, 2H), 3.92 (s, 2H); ^{13}C NMR (DMSO-d_6, 100 MHz): δ = 147.28, 127.59, 122.83, 109.40, 108.26, 101.20, 42.02.

2-phenylethaneamine hydrochloride (**2v**) [S24]

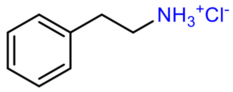

^{1}H NMR (DMSO-d_6, 400 MHz): δ = 8.07 (br s, 3H), 7.35–7.31 (m, 2H), 7.27–7.25 (m, 3H), 3.04–2.99 (m, 2H), 2.92–2.88 (m, 2H); ^{13}C NMR (DMSO-d_6, 100 MHz): δ = 137.32, 128.54, 128.51, 126.60, 39.79, 32.86.

p-phenylbenzylamine hydrochloride (**2w**) [S17, S25]

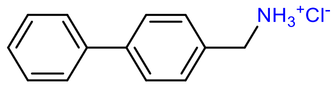

^{1}H NMR (DMSO-d_6, 400 MHz): δ = 8.61 (br s, 3H), 7.73–7.59 (m, 6H), 7.50–7.46 (m, 2H), 7.40–7.37 (m, 1H), 4.06 (s, 2H); ^{13}C NMR (DMSO-d_6, 100 MHz): δ = 140.20, 139.52, 133.28, 129.58, 128.98, 127.68, 126.77, 126.69, 41.82.

1-naphthalenemethylamine hydrochloride (**2x**) [S26]

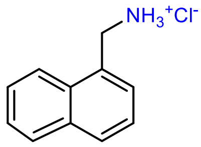

^{1}H NMR (DMSO-d_6, 400 MHz): δ = 8.39 (br s, 3H), 8.16–8.14 (m, 1H), 8.03–7.98 (m, 2H), 7.67–7.55 (m, 4H), 4.53 (s, 2H); ^{13}C NMR (DMSO-d_6, 100 MHz): δ = 133.23, 130.64, 129.94, 129.11, 128.66, 127.27, 126.74, 126.26, 125.35, 123.42, 38.94.

4-picolylamine hydrochloride (**2y**) [S23]

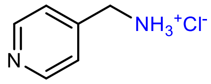

^{1}H NMR (DMSO-d_6, 400 MHz): δ = 9.06 (br s, 3H), 8.92 (d, J = 6.4 Hz, 2H), 8.08 (d, J = 6.4 Hz, 2H), 4.33 (s, 2H); ^{13}C NMR (DMSO-d_6, 100 MHz): δ = 151.68, 143.29, 125.77, 41.03.

3-picolylamine hydrochloride (**2z**) [S27]

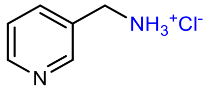

^{1}H NMR (DMSO-d_6, 400 MHz): δ = 8.95 (s, 1H), 8.83–8.81 (m, 1H), 8.68 (br s, 3H), 8.47–8.43 (m, 1H), 7.92–7.88 (m, 1H), 4.21 (q, J = 6.0 Hz, 2H); ^{13}C NMR (DMSO-d_6, 100 MHz): δ = 145.11, 143.70, 142.77, 133.34, 126.26, 39.02.

5-aminomethylindole (**2aa**) [S28, S29]

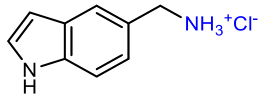

^{1}H NMR (DMSO-d_6, 400 MHz): δ = 10.95 (s, 1H), 7.43 (s, 1H), 7.33–7.27 (m, 2H), 7.04 (d, J = 8.4 Hz, 1H), 6.35 (s, 1H), 3.78 (br s, 2H), 1.78 (br s, 2H); ^{13}C NMR (DMSO-d_6, 100 MHz): δ = 134.85, 127.54, 125.18, 121.04, 118.12, 110.95, 100.82, 46.30.

furfurylamine hydrochloride (**2bb**) [S17]

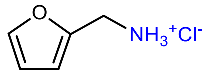

^{1}H NMR (DMSO-d_6, 400 MHz): δ = 8.50 (br s, 3H), 7.73–7.72 (m, 1H), 6.56–6.55 (m, 1H), 6.50–6.49 (m, 1H), 4.06 (s, 2H); ^{13}C NMR (DMSO-d_6, 100 MHz): δ = 147.59, 143.71, 111.04, 110.43, 35.10.

2-thiophenemethylamine hydrochloride (**2cc**) [S17]

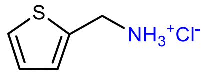

^{1}H NMR (DMSO-d_6, 400 MHz): δ = 8.57 (br s, 3H), 7.58–7.57 (m, 1H), 7.29–7.28 (m, 1H), 7.08–7.06 (m, 1H), 4.21 (s, 2H); ^{13}C NMR (DMSO-d_6, 100 MHz): δ = 135.33, 129.08, 127.24, 127.20, 36.62.

1-pentanamine hydrochloride (**2dd**) [S30]

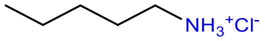

^{1}H NMR (DMSO-d_6, 400 MHz): δ = 8.04 (s, 3H), 2.73 (t, J = 7.6 Hz, 2H), 1.56–1.54 (m, 2H), 1.30–1.28 (m, 4H), 0.87–0.86 (m, 3H); ^{13}C NMR (DMSO-d_6, 100 MHz): δ = 38.55, 27.86, 26.47, 21.50, 13.60.

1-octanamine hydrochloride (**2ee**) [S16]

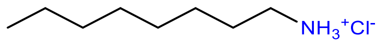

^{1}H NMR (DMSO-d_6, 400 MHz): δ = 8.06 (br s, 3H), 2.73 (t, *J* = 8.0 Hz, 2H), 1.57–1.51 (m, 2H), 1.32–1.19 (m, 10H), 0.88–0.85 (m, 3H); ^{13}C NMR (DMSO-d_6, 100 MHz): δ = 38.61, 31.08, 28.41, 28.35, 26.84, 25.78, 21.99, 13.87.

1-decanamine hydrochloride (**2ff**) [S15]

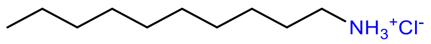

^{1}H NMR (DMSO-d_6, 400 MHz): δ = 7.75 (br s, 3H), 2.75 (t, *J* = 7.2 Hz, 2H), 1.54–1.50 (m, 2H), 1.28–1.22 (m, 14H), 0.86 (t, *J* = 7.2 Hz, 3H); ^{13}C NMR (DMSO-d_6, 100 MHz): δ = 38.78, 31.20, 28.81, 28.76, 28.62, 28.46, 26.91, 25.74, 22.03, 13.91.

1-dodecanamine hydrochloride (**2gg**) [S31]

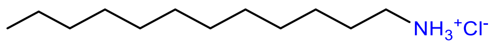

^{1}H NMR (DMSO-d_6, 400 MHz): δ = 7.80 (br s, 3H), 2.75 (t, *J* = 7.2 Hz, 2H), 1.54–1.49 (m, 2H), 1.31–1.22 (m, 18H), 0.86 (t, *J* = 7.2 Hz, 3H); ^{13}C NMR (DMSO-d_6, 100 MHz): δ = 38.78, 31.27, 29.01, 28.99, 28.91, 28.81, 28.69, 28.50, 26.97, 25.78, 22.07, 13.95.

neopentylamine hydrochloride (**2hh**) [S30]

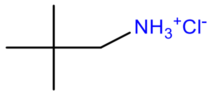

^{1}H NMR (DMSO-d_6, 400 MHz): δ = 7.97 (br s, 3H), 2.60 (s, 2H), 0.95 (s, 9H); ^{13}C NMR (DMSO-d_6, 100 MHz): δ = 49.72, 30.15, 26.85.

cyclopropanemethylamine hydrochloride (**2ii**) [S27]

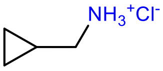

^{1}H NMR (DMSO-d_6, 400 MHz): δ = 8.10 (br s, 3H), 2.68–2.62 (m, 2H), 1.08–0.98 (m, 1H), 0.58–0.46 (m, 2H), 0.38–0.27 (m, 2H); ^{13}C NMR (DMSO-d_6, 100 MHz): δ = 43.41, 8.52, 3.77.

cyclohexanemethylamine hydrochloride (**2jj**) [S17]

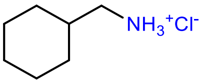

^{1}H NMR (DMSO-d_6, 400 MHz): δ = 8.09 (s, 3H), 2.60 (d, J = 6.8 Hz, 2H), 1.76–1.56 (m, 6H), 1.20–1.10 (m, 3H), 0.95–0.87 (m, 2H); ^{13}C NMR (DMSO-d_6, 100 MHz): δ = 44.30, 35.33, 29.75, 25.61, 25.01.

1-adamantanemethylamine hydrochloride (**2kk**) [S2, S32]

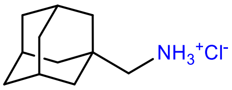

^{1}H NMR (DMSO-d_6, 400 MHz): δ = 7.96 (s, 3H), 2.46 (s, 2H), 1.96 (s, 3H), 1.69–1.52 (m, 12H); ^{13}C NMR (DMSO-d_6, 100 MHz): δ = 49.84, 38.82, 36.05, 31.55, 27.32.

N,N-dimethyl-1,3-propanediamine hydrochloride (**2ll**) [Consistent with an authentic sample prepared from a commercial **2ll**]

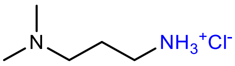

^{1}H NMR (DMSO-d_6, 400 MHz): δ = 8.37 (br s, 3H), 3.19–3.15 (m, 2H), 2.92–2.88 (m, 2H), 2.73 (s, 6H), 2.06–2.02 (m, 2H); ^{13}C NMR (DMSO-d_6, 100 MHz): δ = 53.40, 41.90, 36.05, 21.86.

3-amino-1-propanol hydrochloride (**2mm**) [Consistent with an authentic sample prepared from a commercial **2mm**]

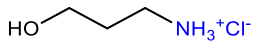

^{1}H NMR (DMSO-d_6, 400 MHz): δ = 8.09 (s, 3H), 5.43 (s, 1H), 3.40 (t, J = 6.0 Hz, 2H), 2.79–2.72 (m, 2H), 1.70–1.63 (m, 2H); ^{13}C NMR (DMSO-d_6, 100 MHz): δ = 57.94, 36.71, 30.12.

3-methoxypropanamine hydrochloride (**2nn**) [S33]

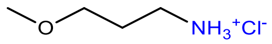

^{1}H NMR (D_2O, 400 MHz): δ = 3.59–3.56 (m, 2H), 3.35 (s, 3H), 3.10–3.07 (m, 2H), 1.96–1.90 (m, 2H); ^{13}C NMR (D_2O, 100 MHz): δ = 71.31, 59.58, 39.22, 27.95.

2-[(3-aminopropyl)amino]ethanol hydrochloride (**2oo**) [Consistent with an authentic sample prepared from a commercial **2oo**]

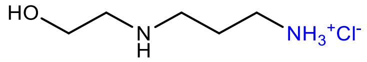

^{1}H NMR (DMSO-d_6, 400 MHz): δ = 6.61–6.48 (m, 5H), 3.57–3.51 (m, 2H), 2.86–2.82 (m, 2H), 2.76–2.73 (m, 2H), 2.70–2.67 (m, 2H), 1.82–1.75 (m, 2H); ^{13}C NMR (DMSO-d_6, 100 MHz): δ = 58.83, 50.59, 45.73, 37.42, 26.00.

3-ethoxypropanamine hydrochloride (**2pp**) [Consistent with an authentic sample prepared from a commercial **2pp**]

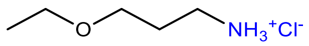

^{1}H NMR (DMSO-d_6, 400 MHz): δ = 8.18 (s, 3H), 3.44–3.40 (m, 4H), 2.81 (t, J = 7.2 Hz, 2H), 1.85–1.79 (m, 2H), 1.11 (t, J = 7.2 Hz, 3H); ^{13}C NMR (DMSO-d_6, 100 MHz): δ =66.67, 65.31, 36.48, 27.20, 15.06.

3-isopropoxypropanamine hydrochloride (**2qq**) [Consistent with an authentic sample prepared from a commercial **2qq**]

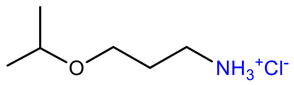

^{1}H NMR (DMSO-d_6, 400 MHz): δ = 8.15 (s, 3H), 3.52 (sep, J = 6.0 Hz, 1H), 3.41 (t, J = 6.0 Hz, 2H), 2.82–2.77 (m, 2H), 1.82–1.75 (m, 2H), 1.09–1.07 (m, 6H); ^{13}C NMR (DMSO-d_6, 100 MHz): δ = 70.62, 64.23, 36.51, 27.55, 21.99.

m-xylylenediamine dihydrochloride (**2rr**) [S2]

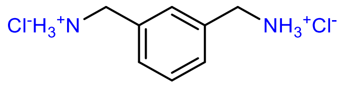

^{1}H NMR (DMSO-d_6, 400 MHz): δ = 8.35 (s, 6H), 7.53–7.49 (m, 4H), 4.03 (s, 4H); ^{13}C NMR (DMSO-d_6, 100 MHz): δ = 134.36, 129.53, 129.00, 128.72, 42.09.

p-xylylenediamine dihydrochloride (**2ss**) [S34]

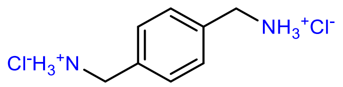

^{1}H NMR (DMSO-d_6, 400 MHz): δ = 8.60 (s, 6H), 7.53 (s, 4H), 4.01 (s, 4H); ^{13}C NMR (DMSO-d_6, 100 MHz): δ = 134.15, 129.01, 41.69.

1,4-butanediamine dihydrochloride (**2tt**) [S16]

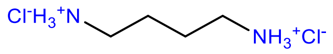

^{1}H NMR (D_2O, 400 MHz): δ = 3.04–3.02 (m, 4H), 1.75–1.72 (m, 4H); ^{13}C NMR (D_2O, 100 MHz): δ = 40.38, 25.44.

1,8-octanediamine dihydrochloride (**2uu**) [S34]

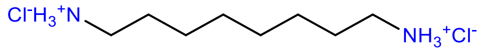

^{1}H NMR (DMSO-d_6, 400 MHz): δ = 7.90 (br s, 6H), 2.75 (t, *J* = 7.6 Hz, 4H), 1.58–1.51 (m, 4H), 1.34–1.26 (m, 8H); ^{13}C NMR (DMSO-d_6, 100 MHz): δ = 38.70, 28.24, 26.88, 25.66.

1,10-decanediamine dihydrochloride (**2vv**) [S34]

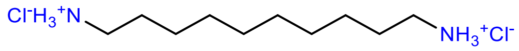

^{1}H NMR (DMSO-d_6, 400 MHz): δ = 7.97 (br s, 6H), 2.73 (t, *J* = 8.0 Hz, 4H), 1.58–1.51 (m, 4H), 1.32–1.23 (m, 12H); ^{13}C NMR (DMSO-d_6, 100 MHz): δ = 38.86, 28.84, 28.65, 27.09, 25.99.

bis(3-aminopropyl)amine dihydrochloride (**2ww**) [Consistent with an authentic sample prepared from a commercial **2ww**]

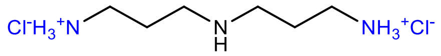

^{1}H NMR (D_2O, 400 MHz): δ = 2.82 (t, *J* = 7.2 Hz, 4H), 2.73–2.69 (m, 4H), 1.77–1.70 (m, 4H); ^{13}C NMR (D_2O, 100 MHz): δ = 47.22, 39.56, 29.48.

3. ^{1}H and ^{13}C NMR spectra

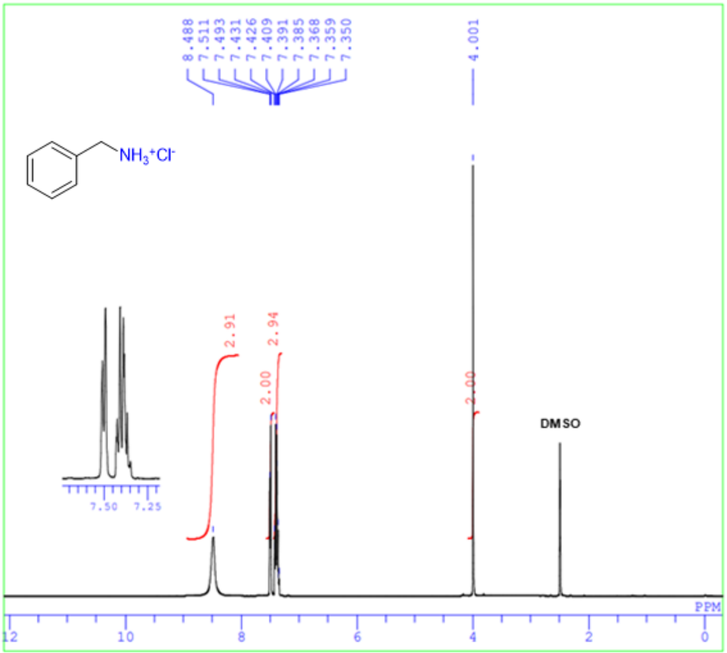

^{1}H NMR spectrum of benzylamine hydrochloride (**2a**)

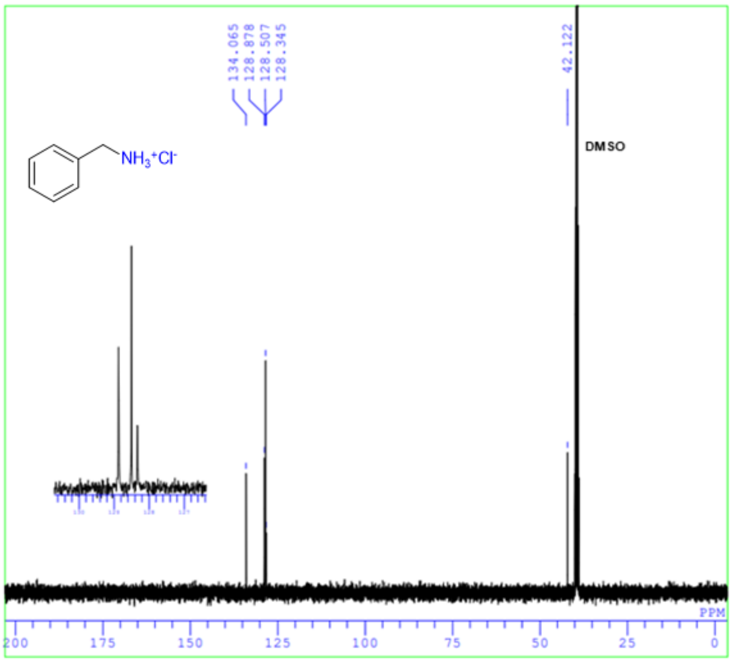

^{13}C NMR spectrum of benzylamine hydrochloride (**2a**)

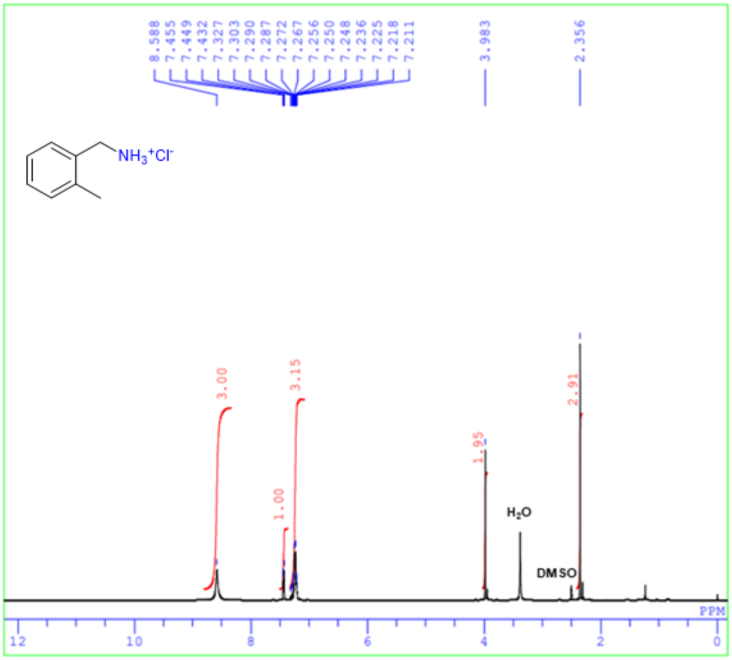

^{1}H NMR spectrum of *o*-methylbenzylamine hydrochloride (**2b**)

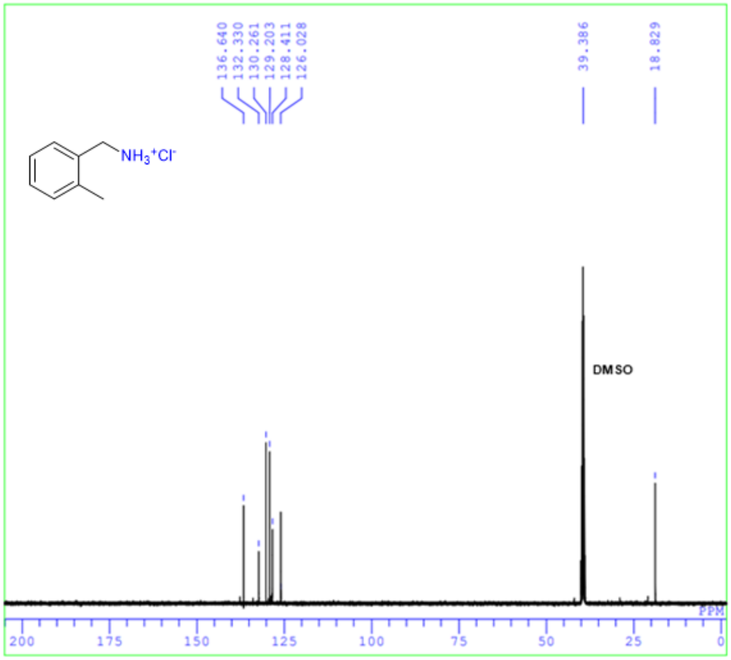

^{13}C NMR spectrum of *o*-methylbenzylamine hydrochloride (**2b**)

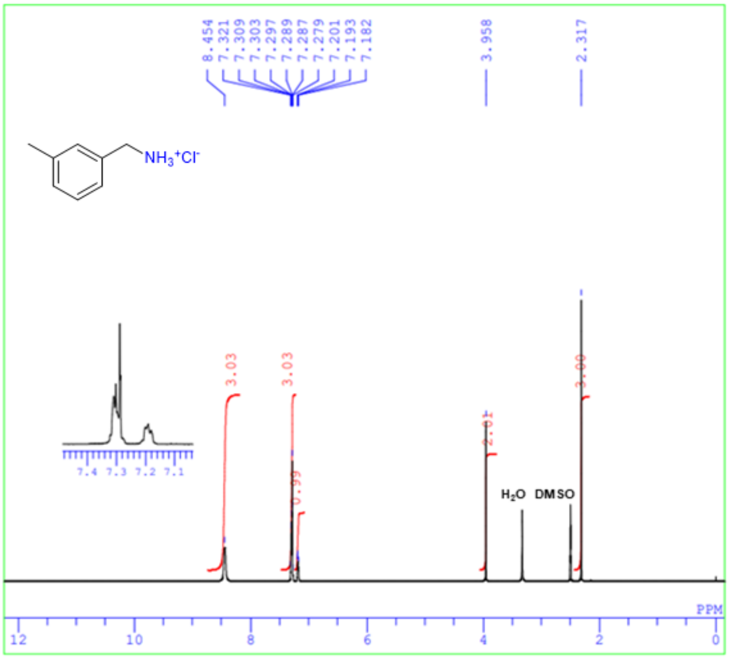

^{1}H NMR spectrum of *m*-methylbenzylamine hydrochloride (**2c**)

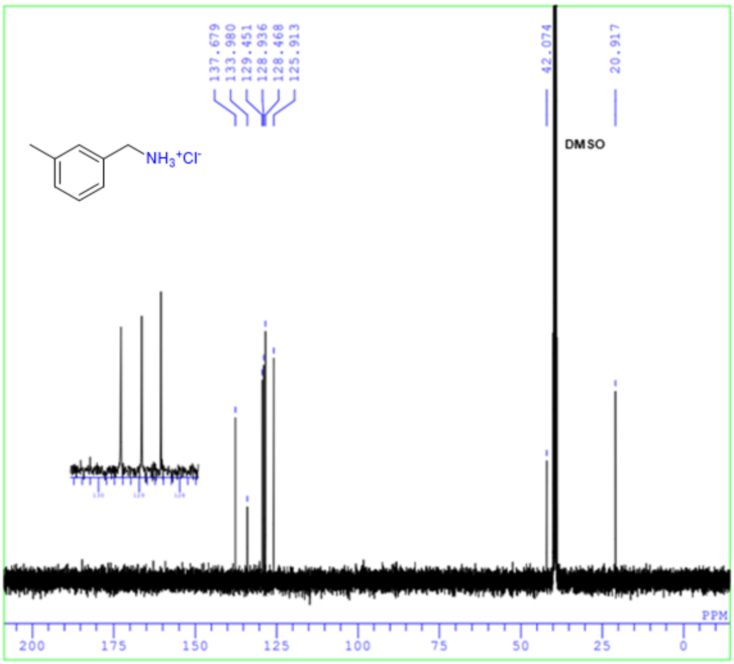

^{13}C NMR spectrum of *m*-methylbenzylamine hydrochloride (**2c**)

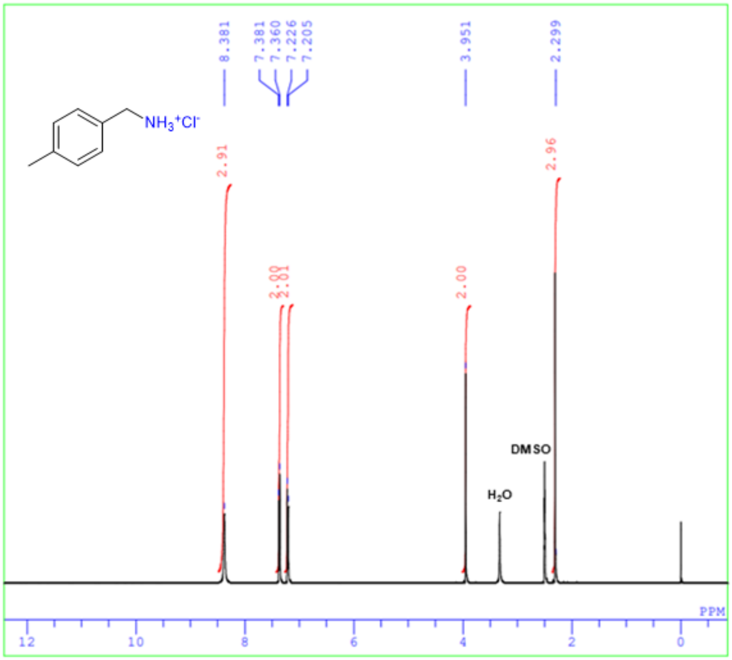

^{1}H NMR spectrum of *p*-methylbenzylamine hydrochloride (**2d**)

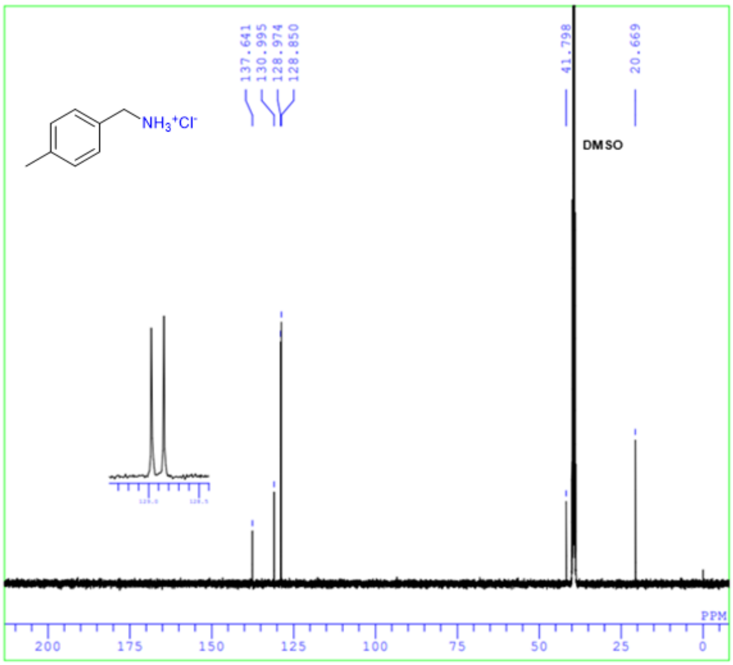

^{13}C NMR spectrum of *p*-methylbenzylamine hydrochloride (**2d**)

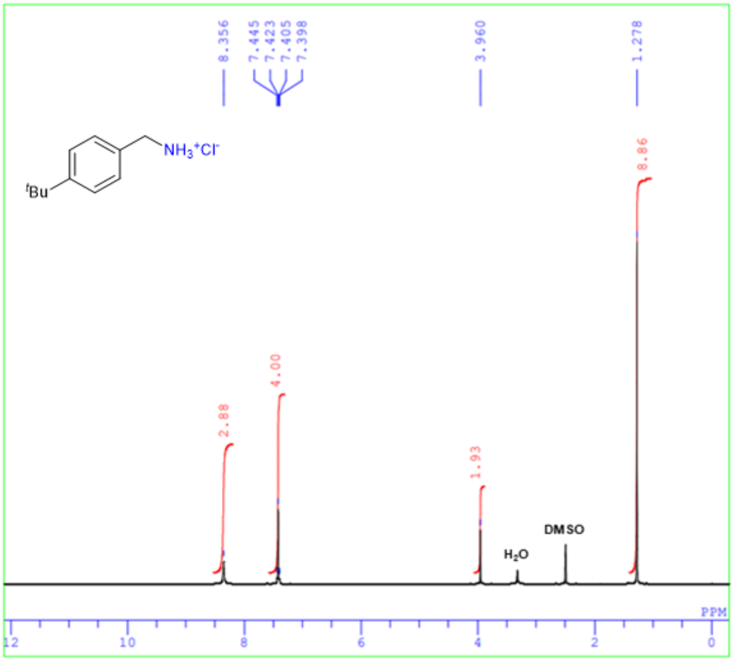

^{1}H NMR spectrum of *p*-*tert*-butylbenzylamine hydrochloride (**2e**)

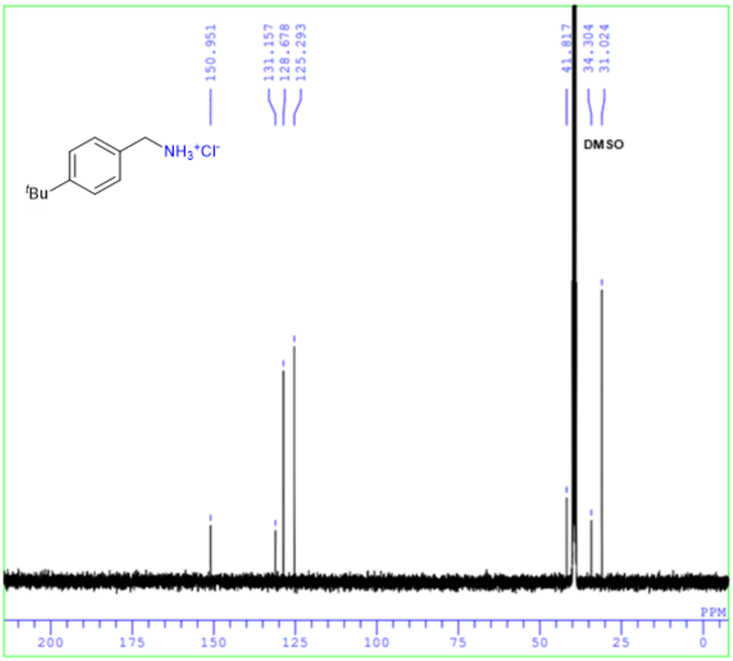

^{13}C NMR spectrum of *p*-*tert*-butylbenzylamine hydrochloride (**2e**)

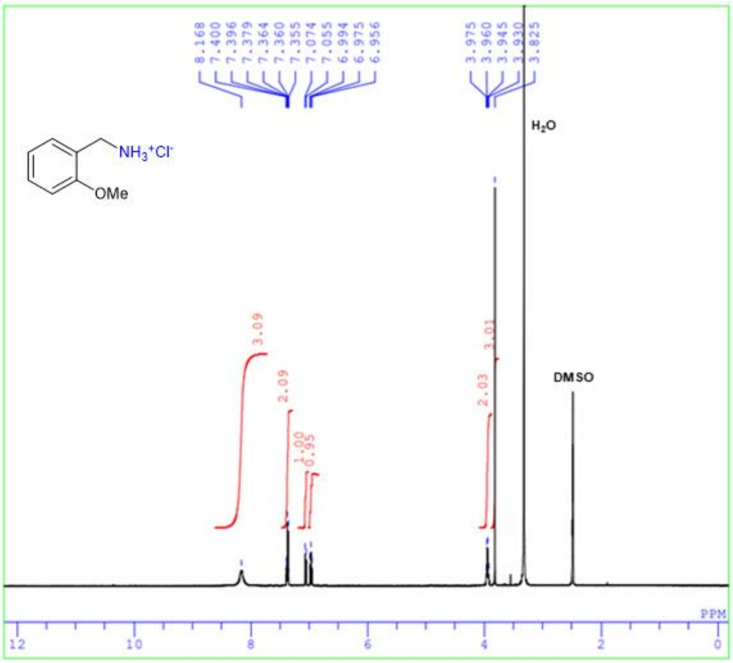

^{1}H NMR spectrum of *o*-methoxybenzylamine hydrochloride (**2f**)

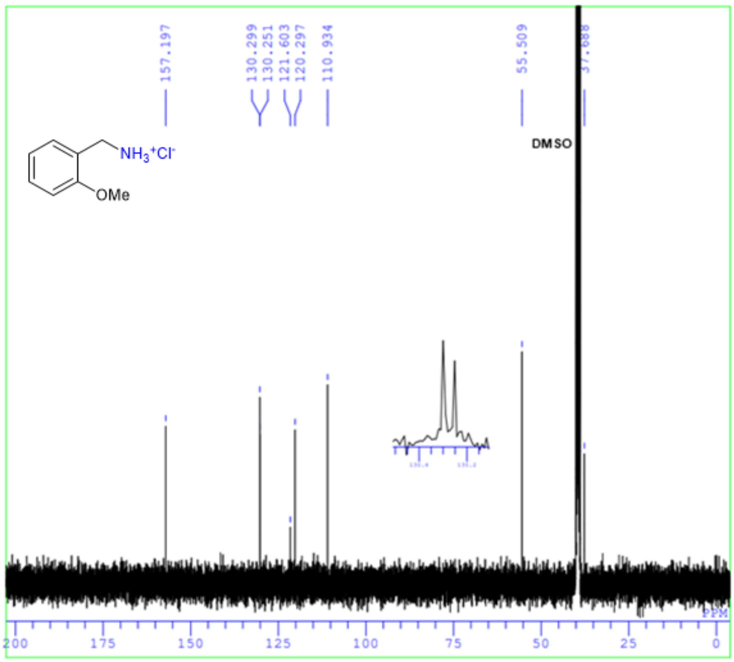

^{13}C NMR spectrum of *o*-methoxybenzylamine hydrochloride (**2f**)

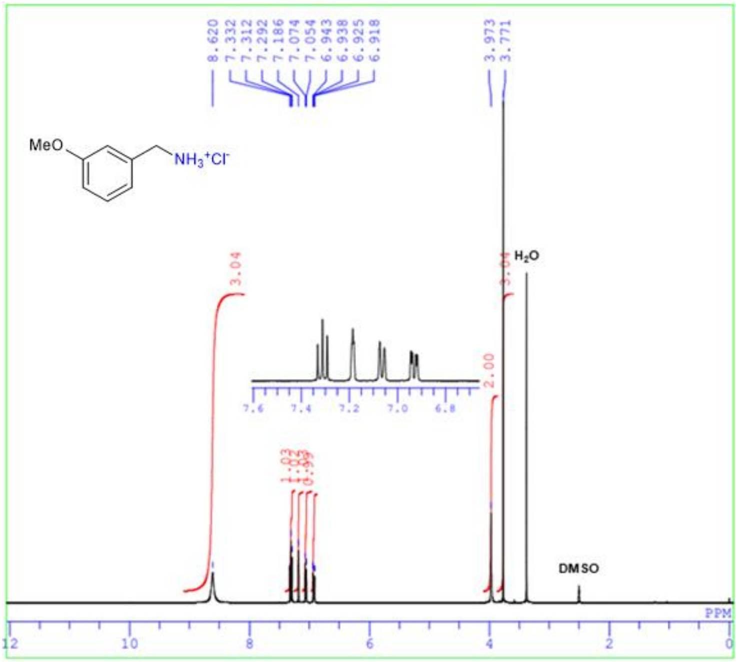

^{1}H NMR spectrum of *m*-methoxybenzylamine hydrochloride (**2g**)

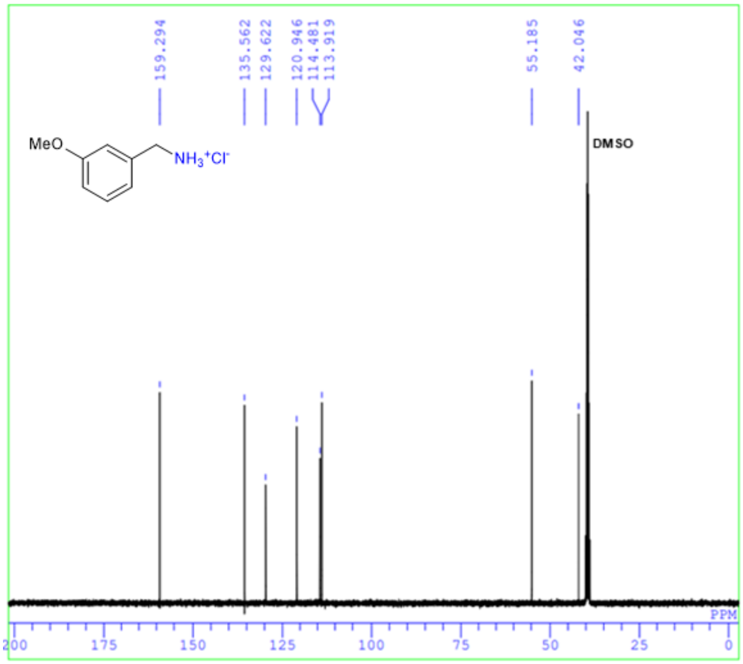

^{13}C NMR spectrum of *m*-methoxybenzylamine hydrochloride (**2g**)

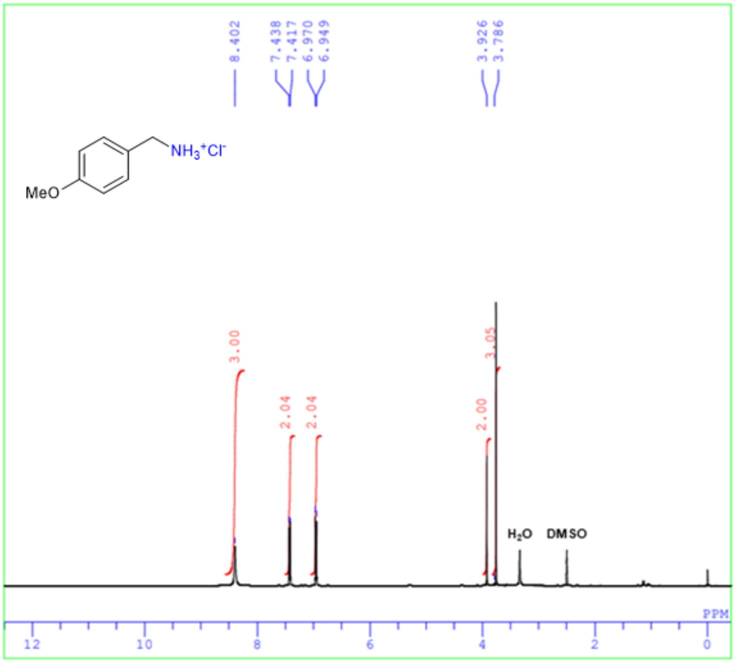

^{1}H NMR spectrum of *p*-methoxybenzylamine hydrochloride (**2h**)

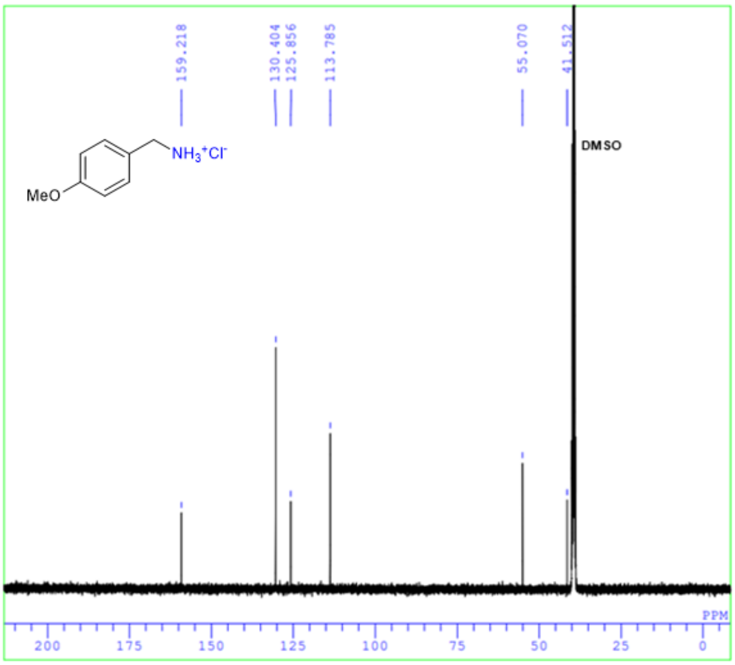

^{13}C NMR spectrum of *p*-methoxybenzylamine hydrochloride (**2h**)

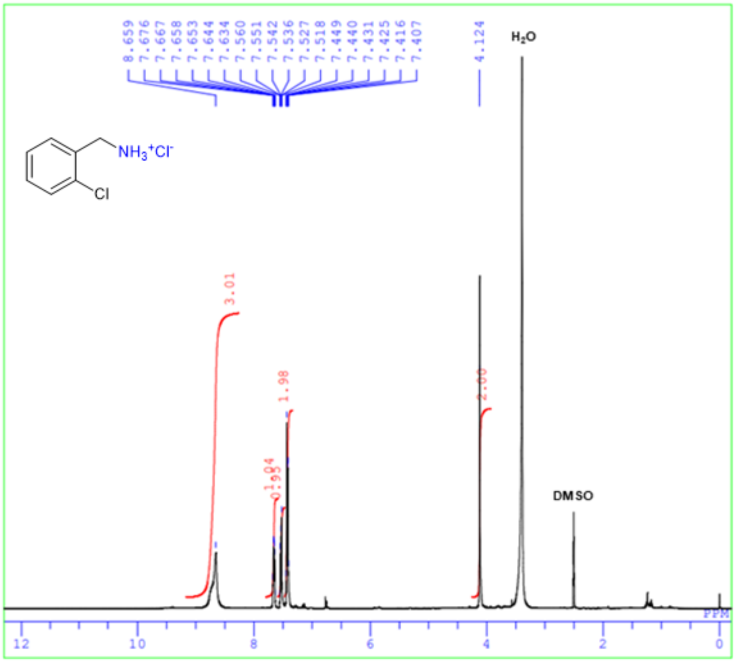

^{1}H NMR spectrum of *o*-chlorobenzylamine hydrochloride (**2i**)

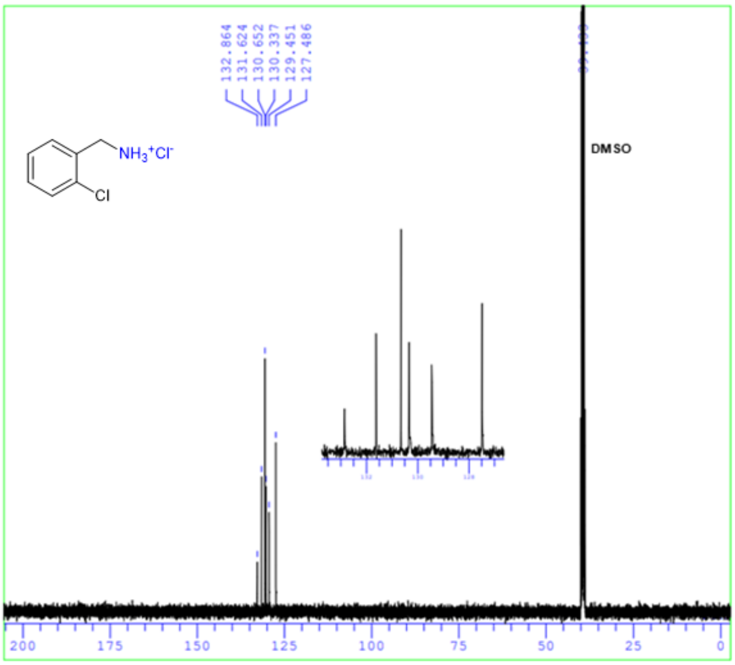

^{13}C NMR spectrum of *o*-chlorobenzylamine hydrochloride (**2i**)

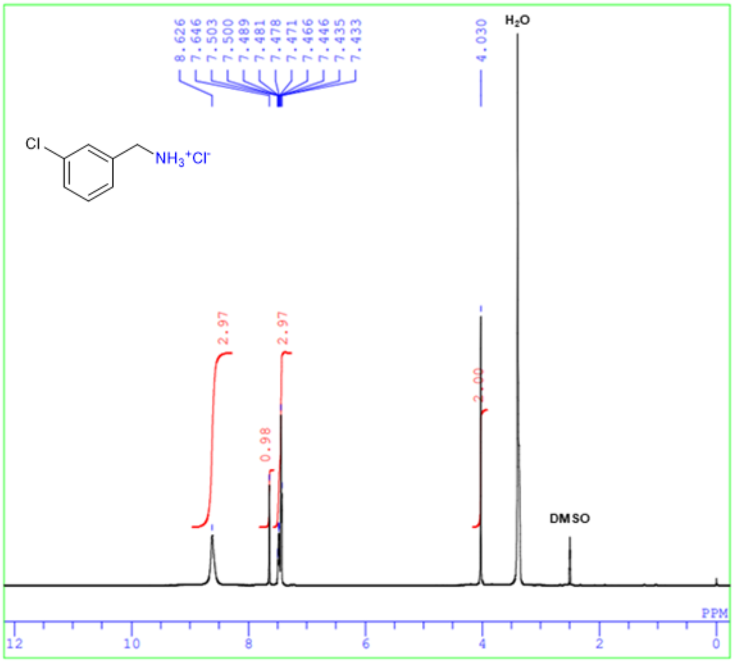

^{1}H NMR spectrum of *m*-chlorobenzylamine hydrochloride (**2j**)

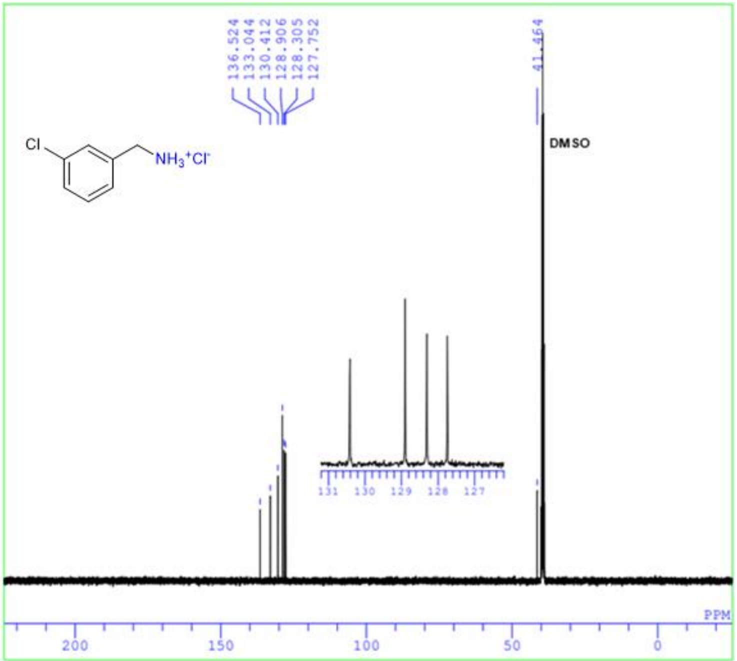

^{13}C NMR spectrum of *m*-chlorobenzylamine hydrochloride (**2j**)

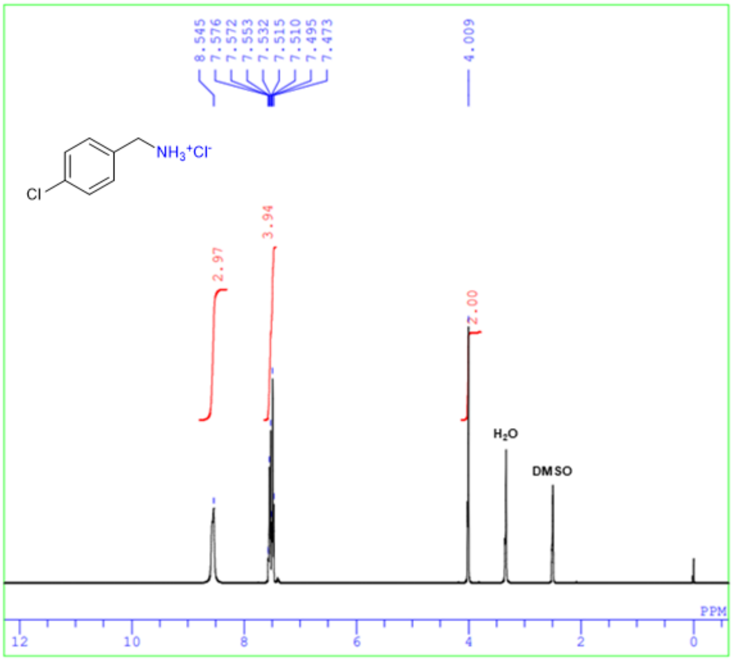

^{1}H NMR spectrum of *p*-chlorobenzylamine hydrochloride (**2k**)

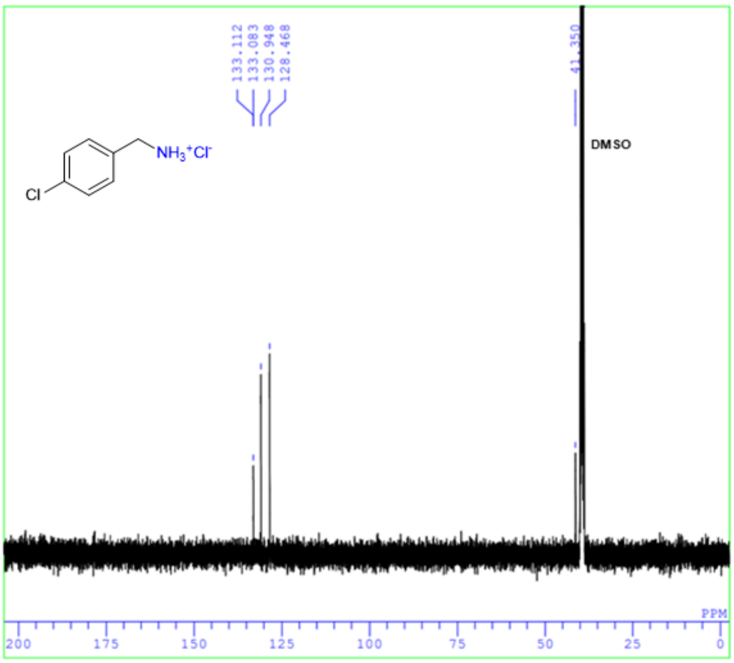

^{13}C NMR spectrum of *p*-chlorobenzylamine hydrochloride (**2k**)

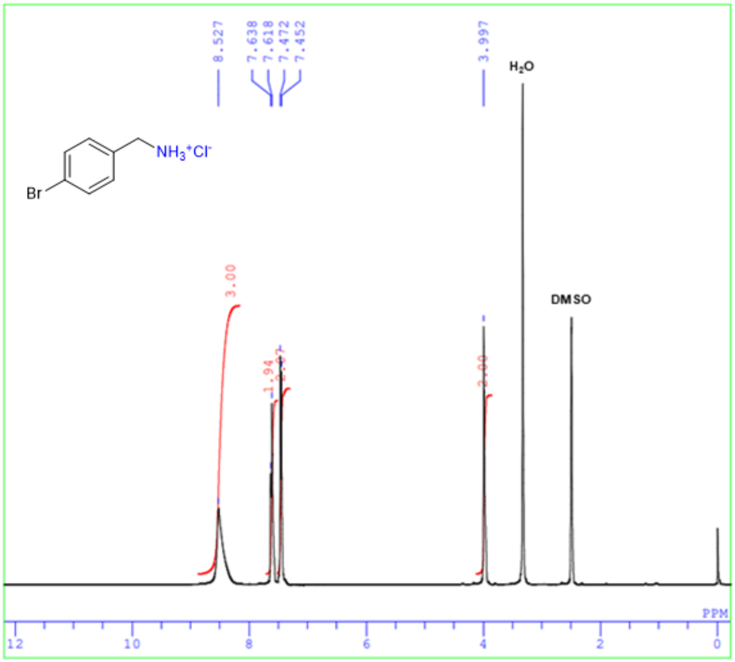

^{1}H NMR spectrum of *p*-bromobenzylamine hydrochloride (**2l**)

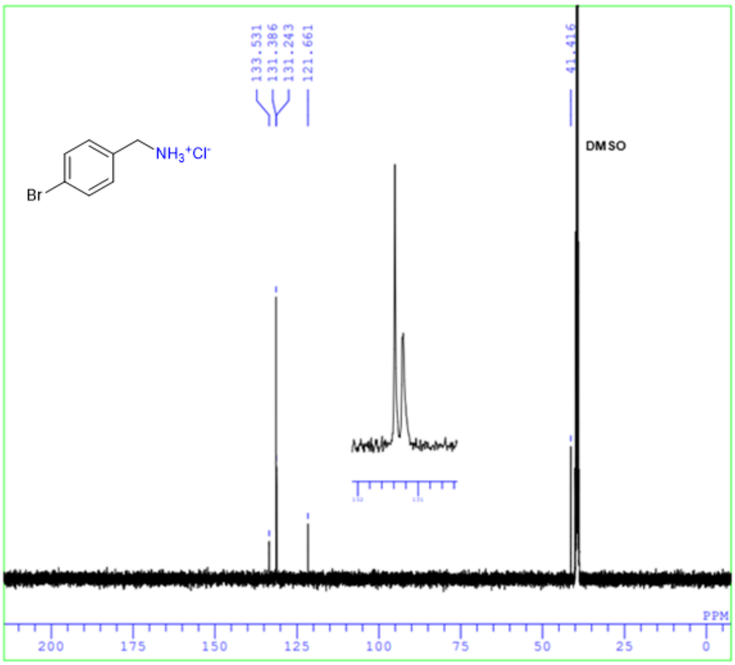

^{13}C NMR spectrum of *p*-bromobenzylamine hydrochloride (**2l**)

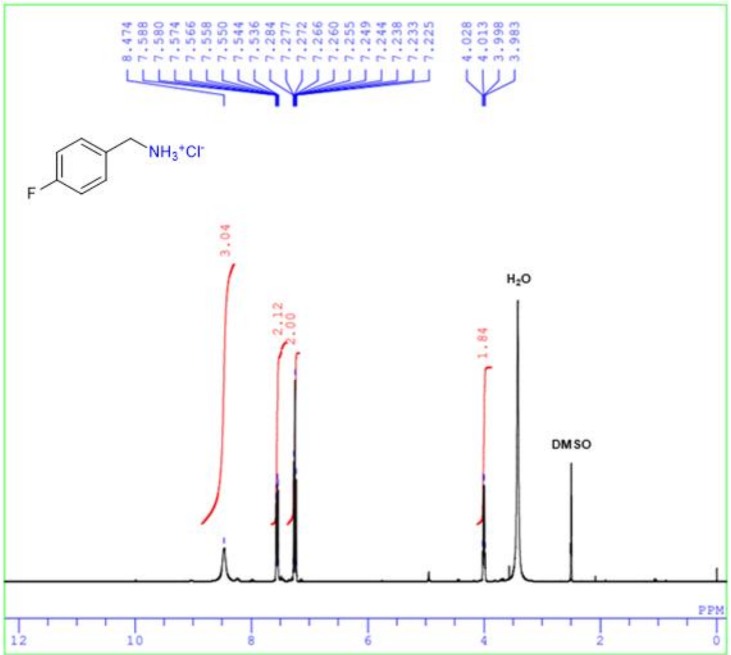

^{1}H NMR spectrum of *p*-fluorobenzylamine hydrochloride (**2m**)

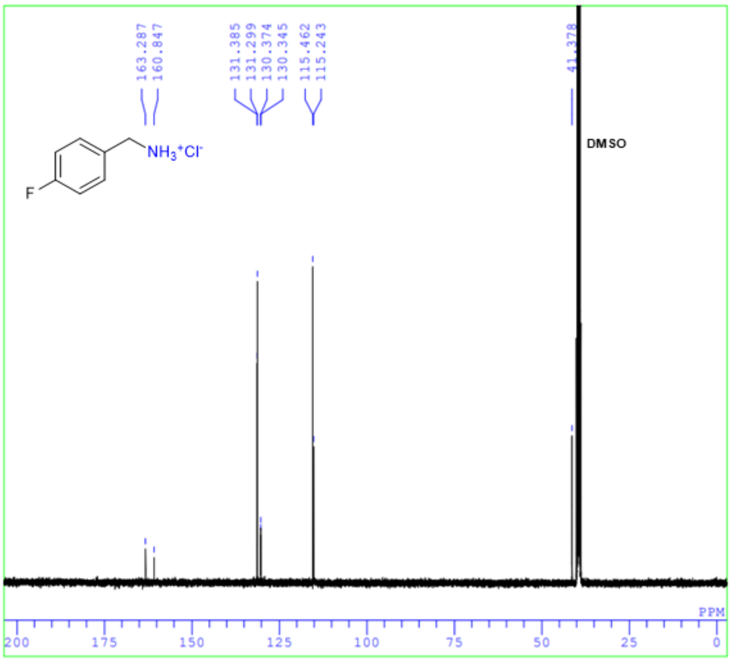

^{13}C NMR spectrum of *p*-fluorobenzylamine hydrochloride (**2m**)

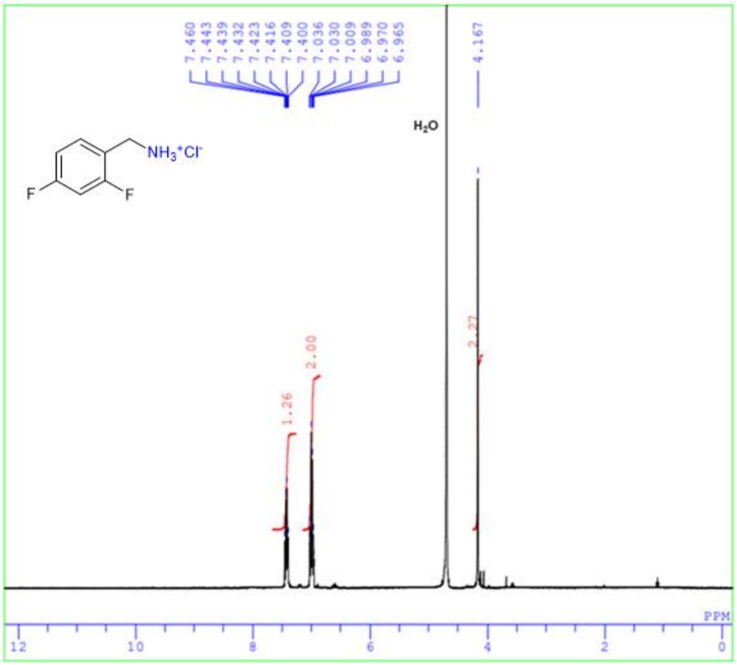

^{1}H NMR spectrum of 2,4-difluorobenzylamine hydrochloride (**2n**)

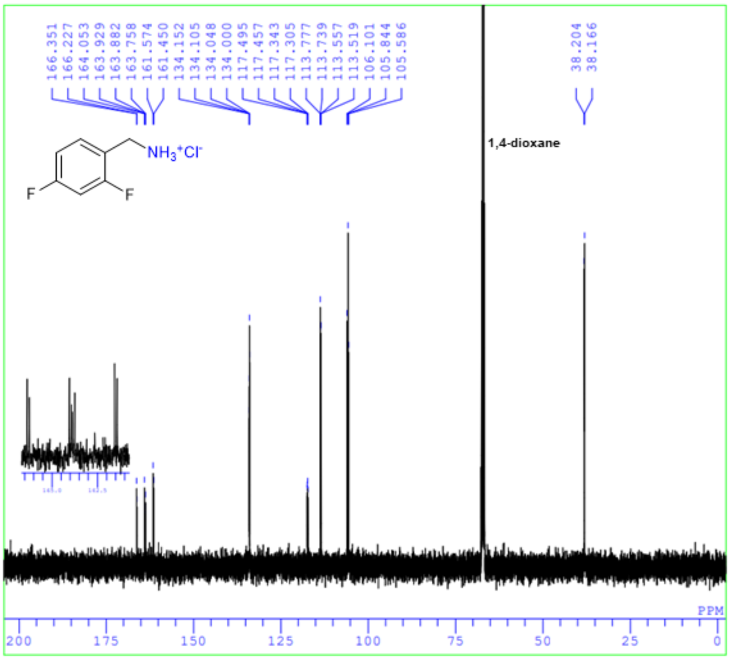

^{13}C NMR spectrum of 2,4-difluorobenzylamine hydrochloride (**2n**)

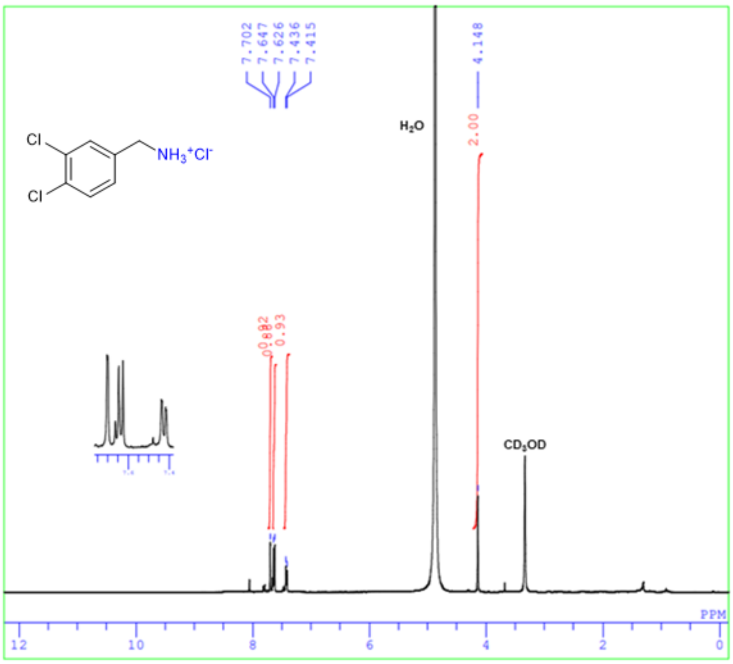

^{1}H NMR spectrum of 3,4-dichlorobenzylamine hydrochloride (**2o**)

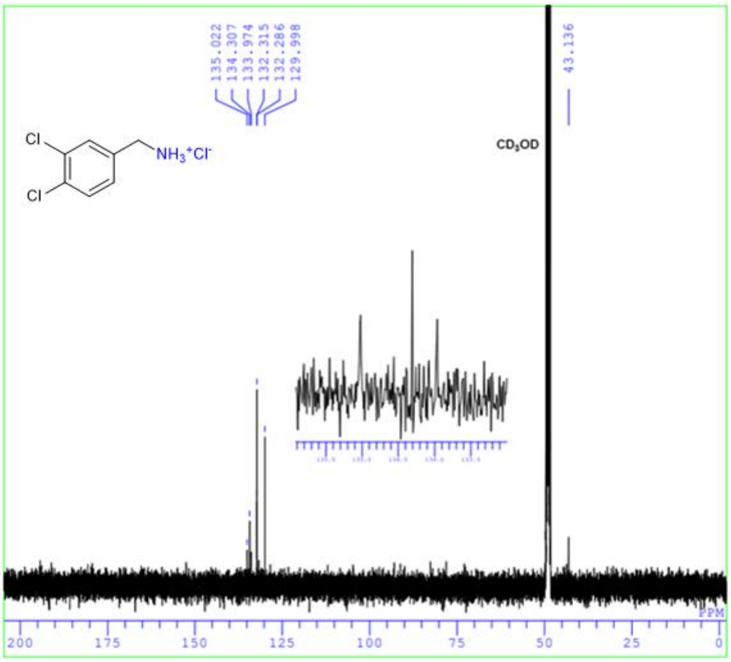

^{13}C NMR spectrum of 3,4-dichlorobenzylamine hydrochloride (**2o**)

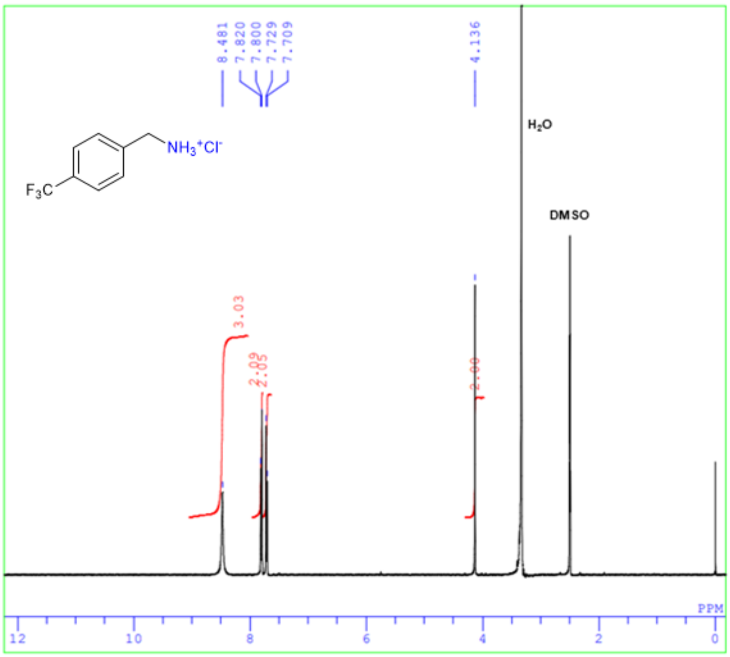

^{1}H NMR spectrum of *p*-(trifluoromethyl)benzylamine hydrochloride (**2p**)

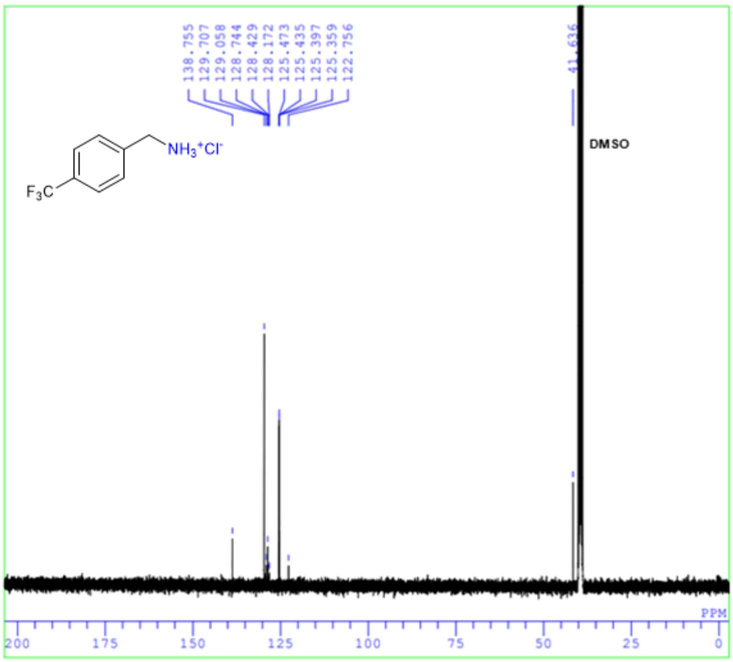

^{13}C NMR spectrum of *p*-(trifluoromethyl)benzylamine hydrochloride (**2p**)

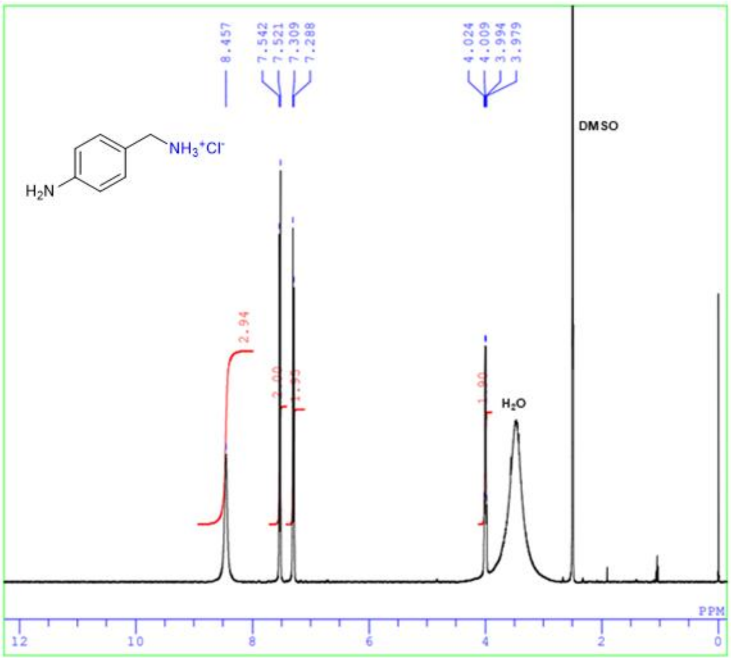

^{1}H NMR spectrum of *p*-aminobenzylamine hydrochloride (**2q**)

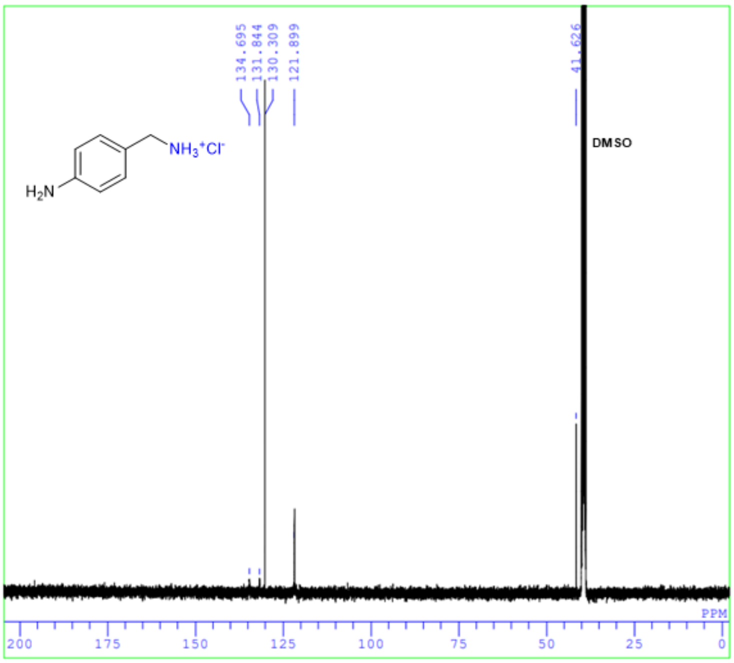

^{13}C NMR spectrum of *p*-aminobenzylamine hydrochloride (**2q**)

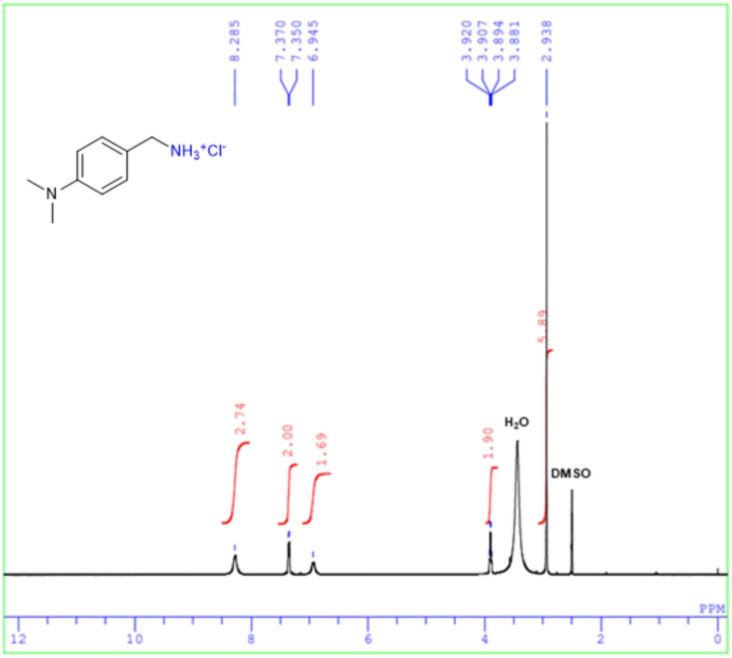

^{1}H NMR spectrum of *p*-(*N*,*N*-dimethylamino)benzylamine hydrochloride (**2r**)

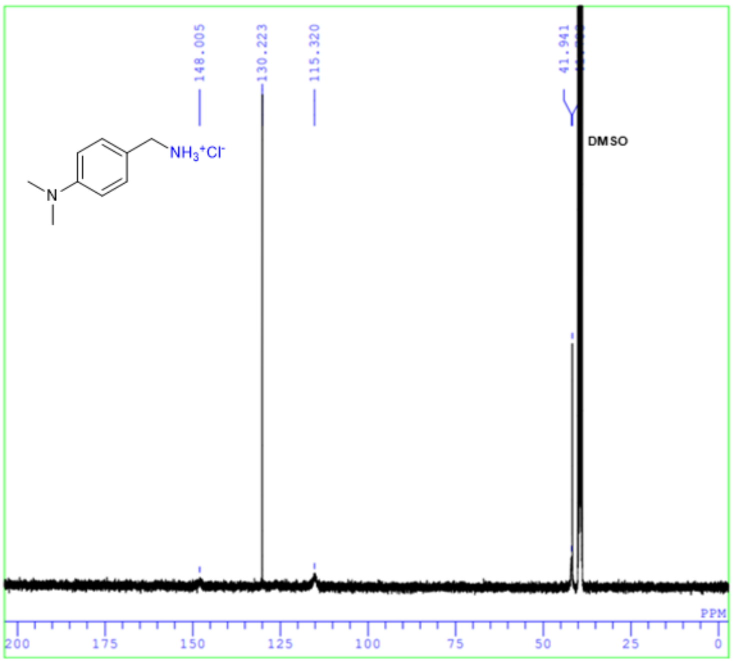

^{13}C NMR spectrum of *p*-(*N*,*N*-dimethylamino)benzylamine hydrochloride (**2r**)

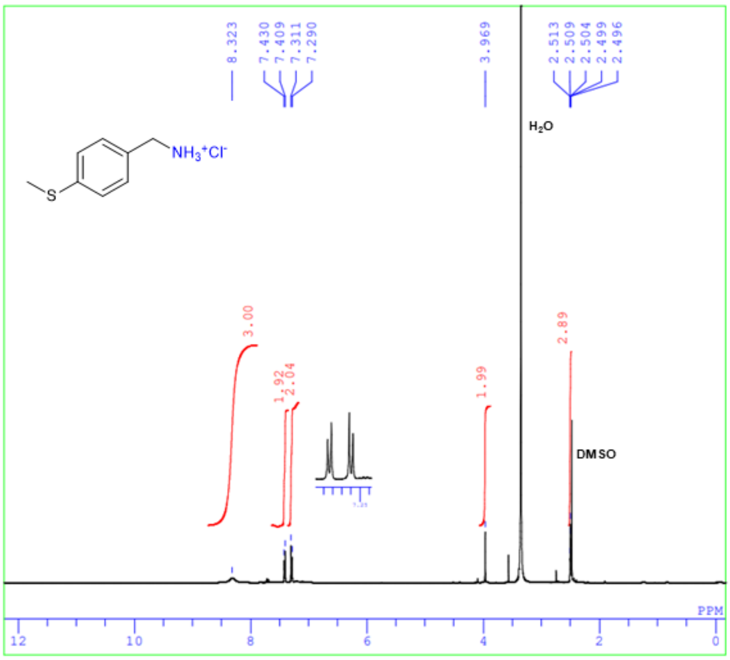

^{1}H NMR spectrum of *p*-(methylthio)benzylamine hydrochloride (**2s**)

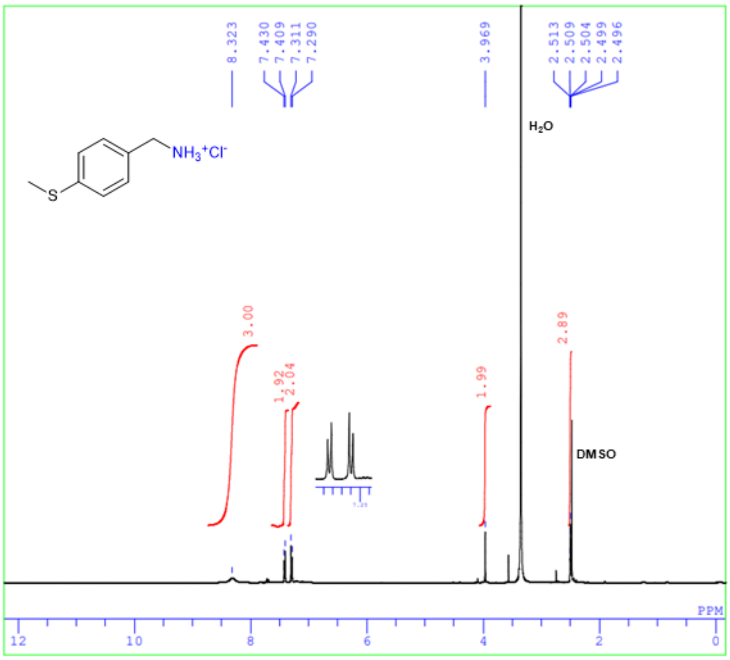

^{13}C NMR spectrum of *p*-(methylthio)benzylamine hydrochloride (**2s**)

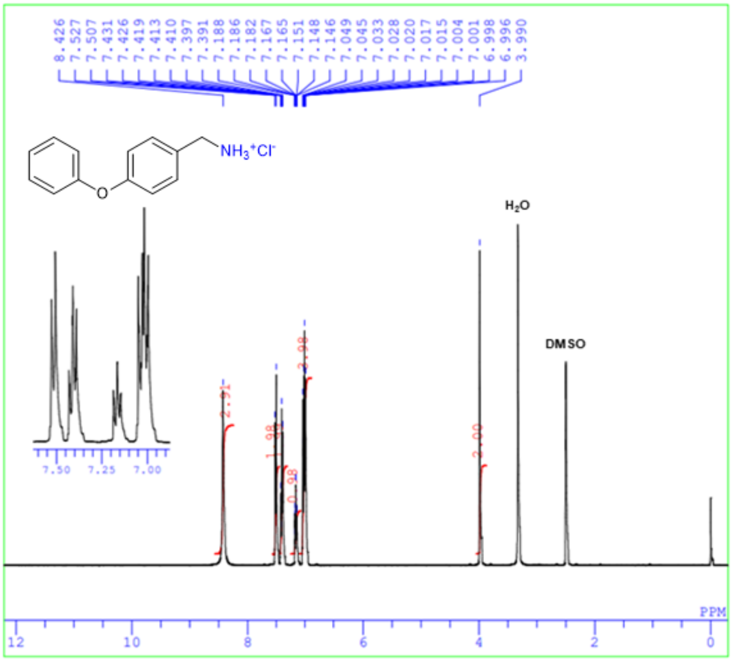

^{1}H NMR spectrum of *p*-phenoxybenzylamine hydrochloride (**2t**)

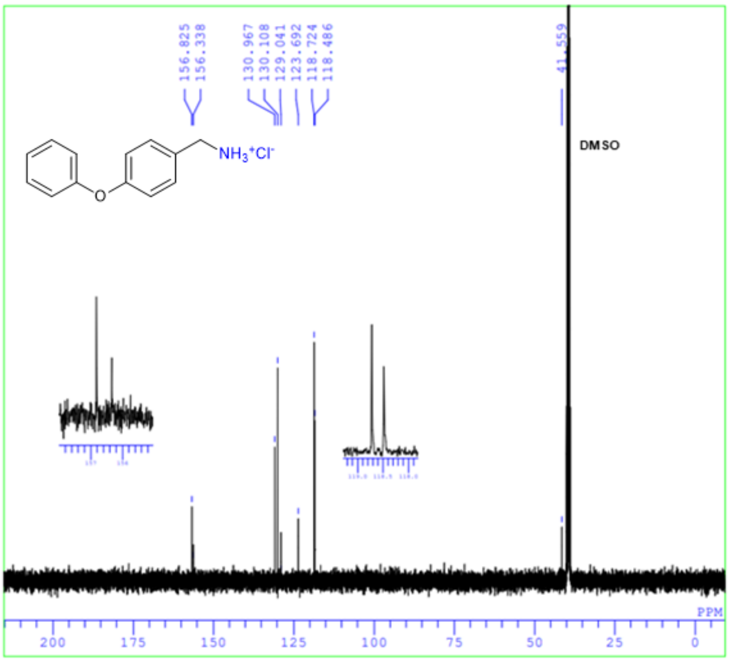

^{13}C NMR spectrum of *p*-phenoxybenzylamine hydrochloride (**2t**)

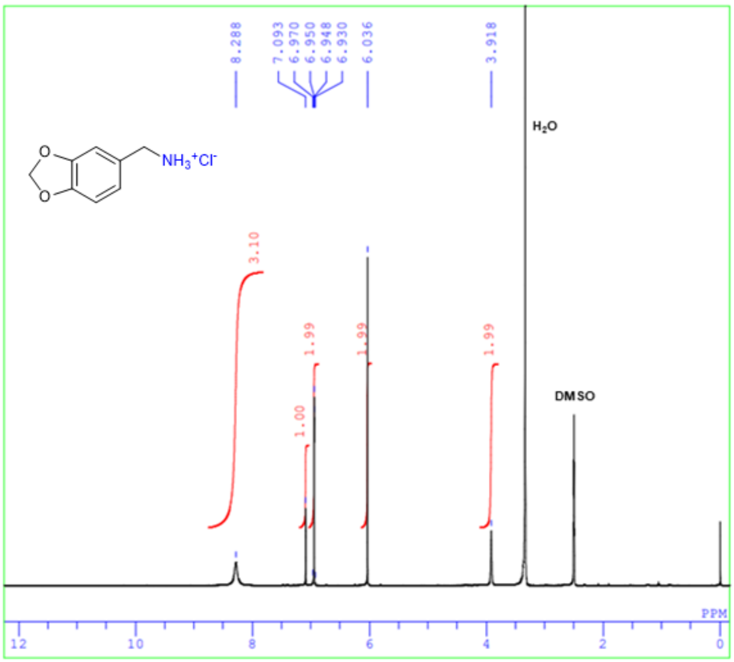

^{1}H NMR spectrum of 3,4-methylenedioxybenzylamine hydrochloride (**2u**)

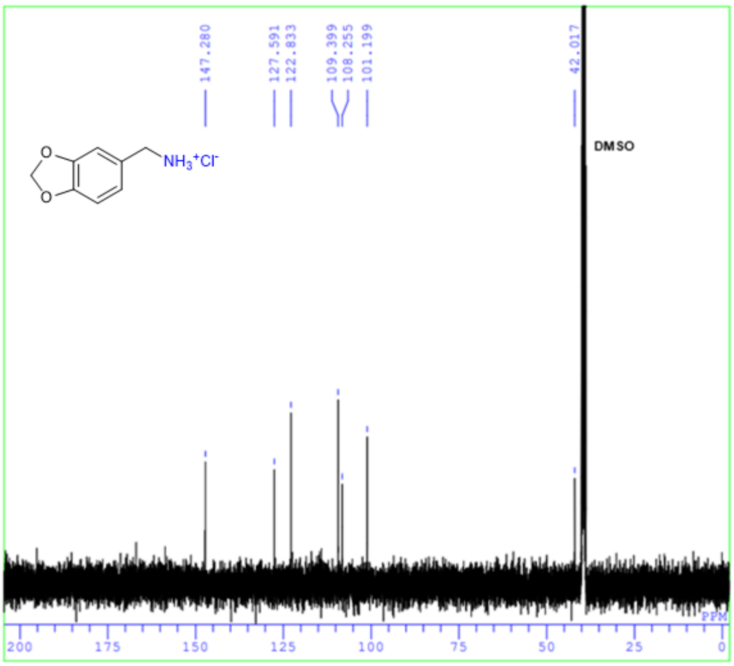

^{13}C NMR spectrum of 3,4-methylenedioxybenzylamine hydrochloride (**2u**)

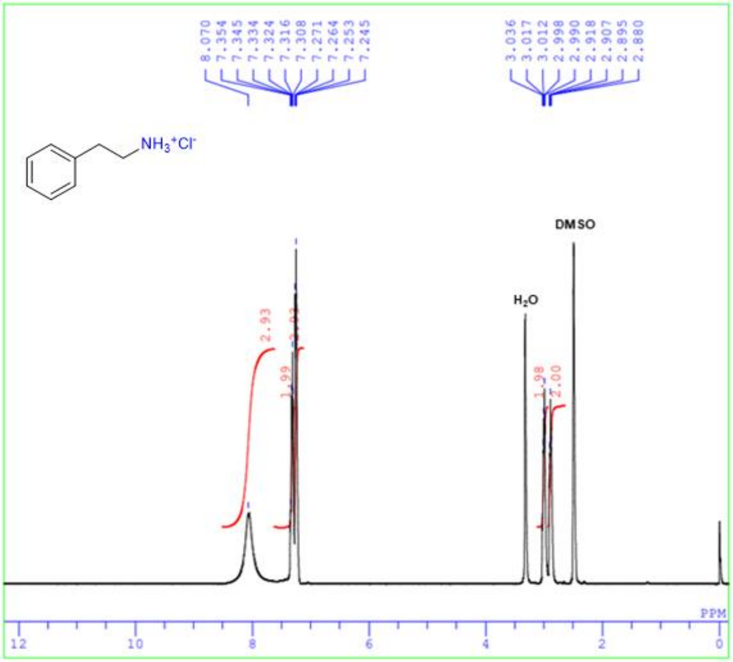

^{1}H NMR spectrum of 2-phenylethaneamine hydrochloride (**2v**)

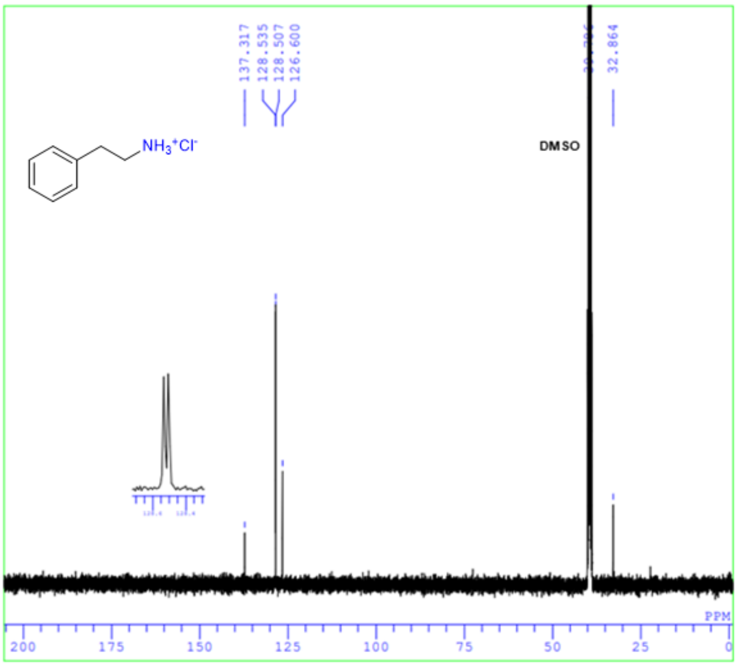

^{13}C NMR spectrum of 2-phenylethaneamine hydrochloride (**2v**)

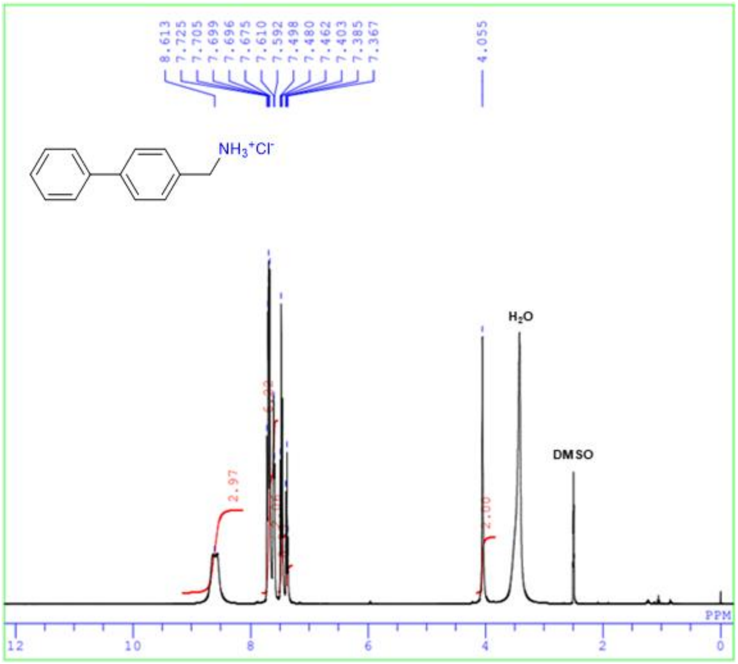

^{1}H NMR spectrum of *p*-phenylbenzylamine hydrochloride (**2w**)

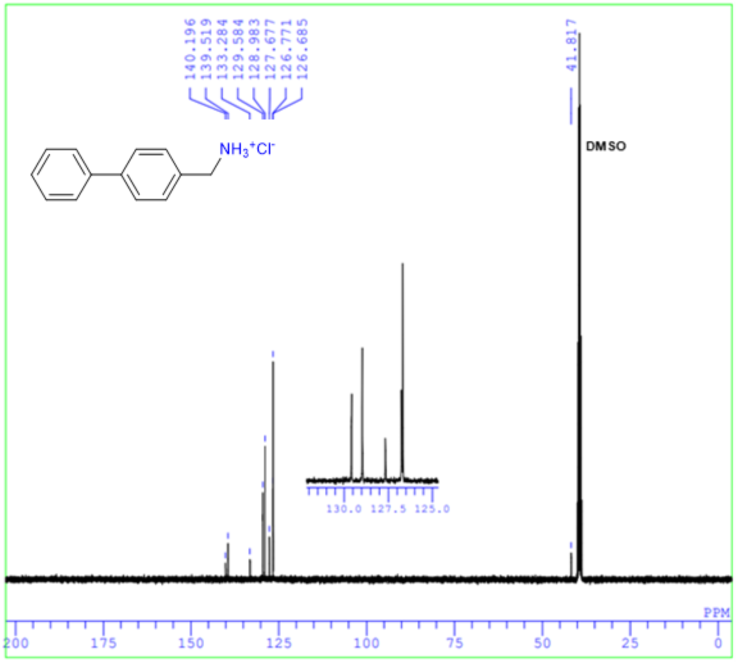

^{13}C NMR spectrum of *p*-phenylbenzylamine hydrochloride (**2w**)

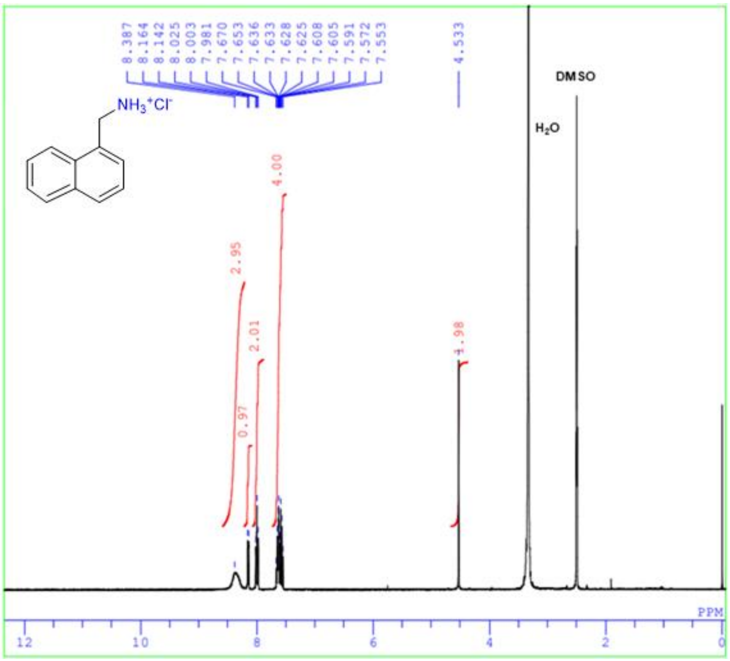

^{1}H NMR spectrum of 1-naphthalenemethylamine hydrochloride (**2x**)

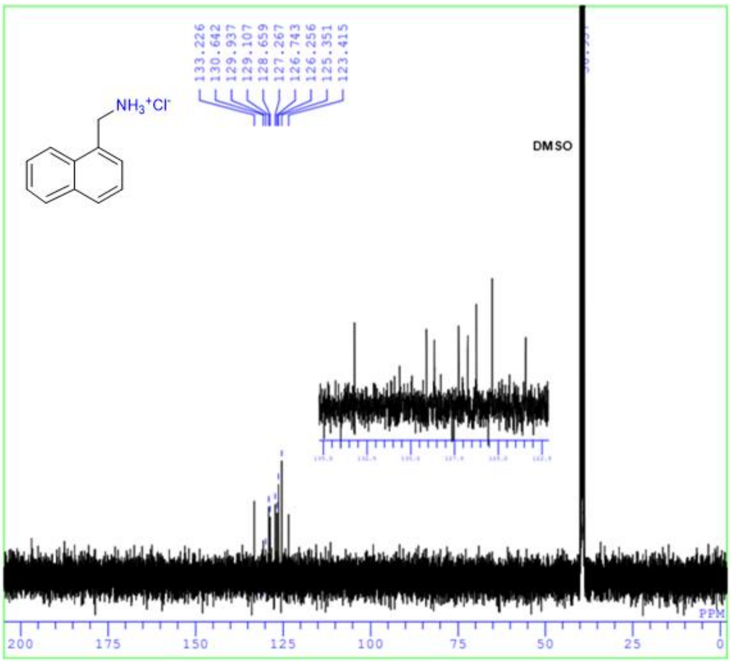

^{13}C NMR spectrum of 1-naphthalenemethylamine hydrochloride (**2x**)

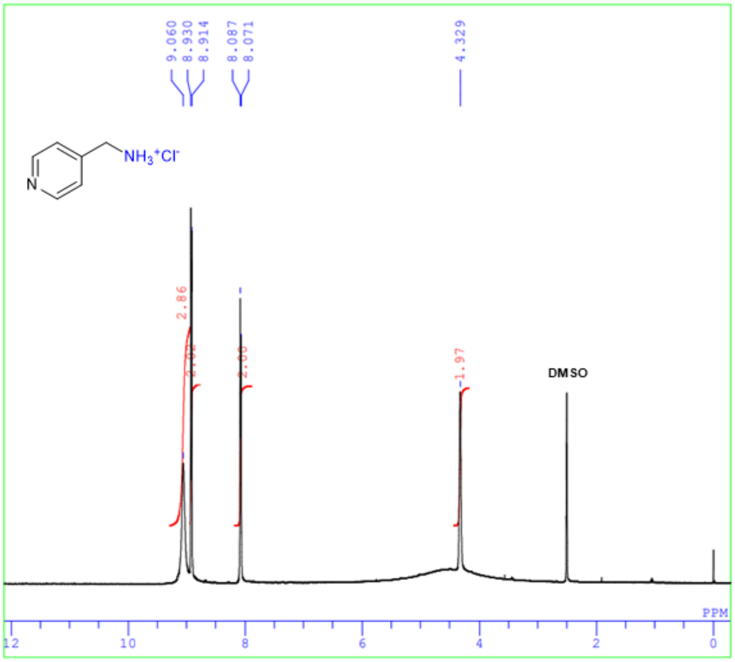

^{1}H NMR spectrum of 4-picolylamine hydrochloride (**2y**)

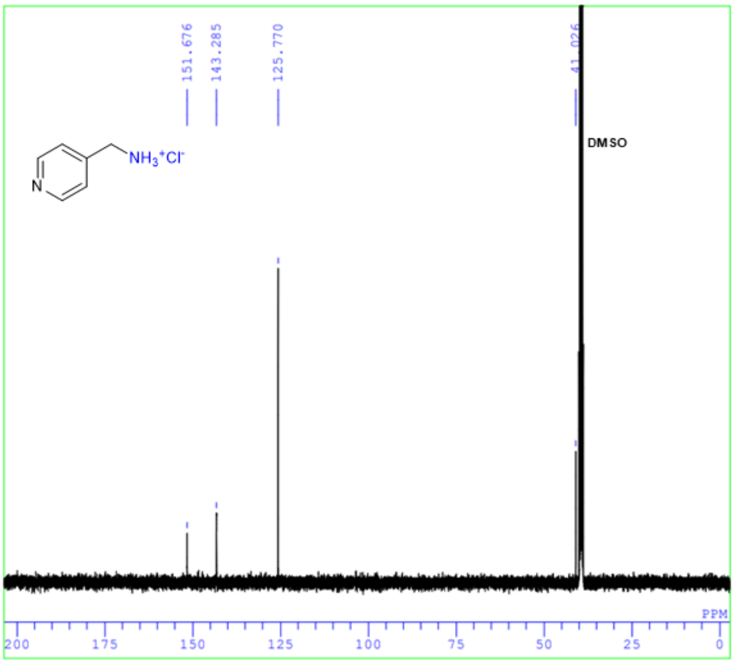

^{13}C NMR spectrum of 4-picolylamine hydrochloride (**2y**)

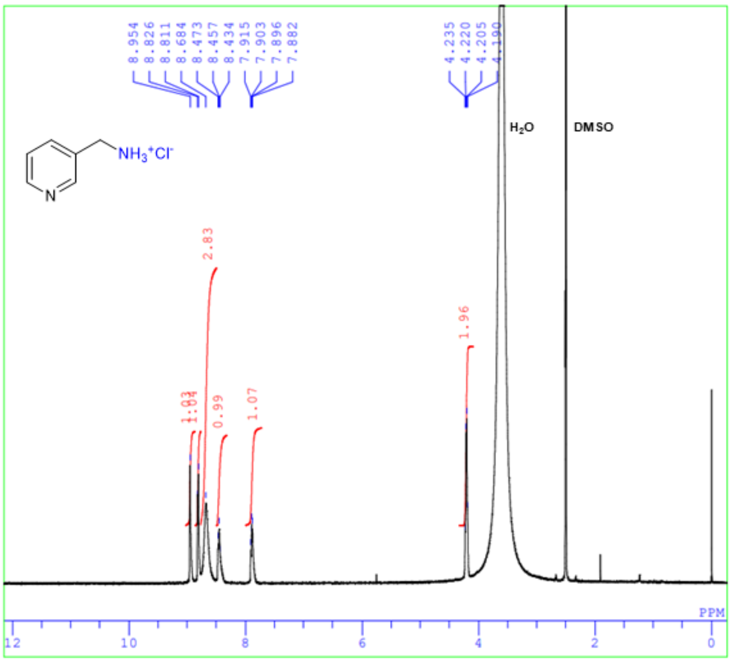

^{1}H NMR spectrum of 3-picolylamine hydrochloride (**2z**)

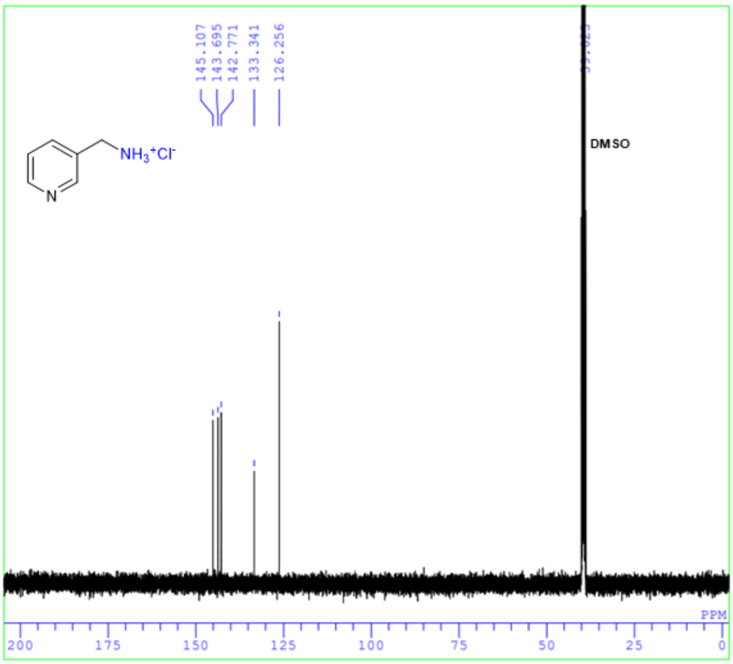

^{13}C NMR spectrum of 3-picolylamine hydrochloride (**2z**)

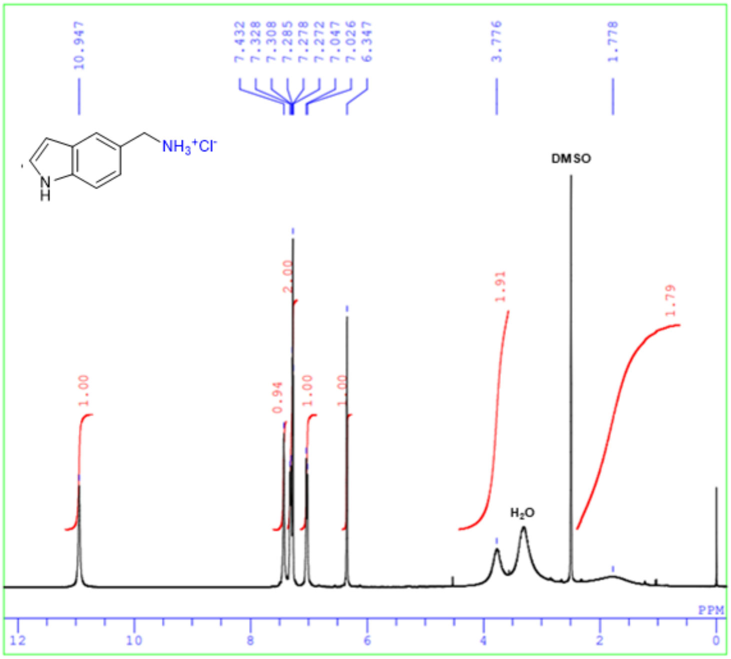

^{1}H NMR spectrum of 5-aminomethylindole (**2aa**)

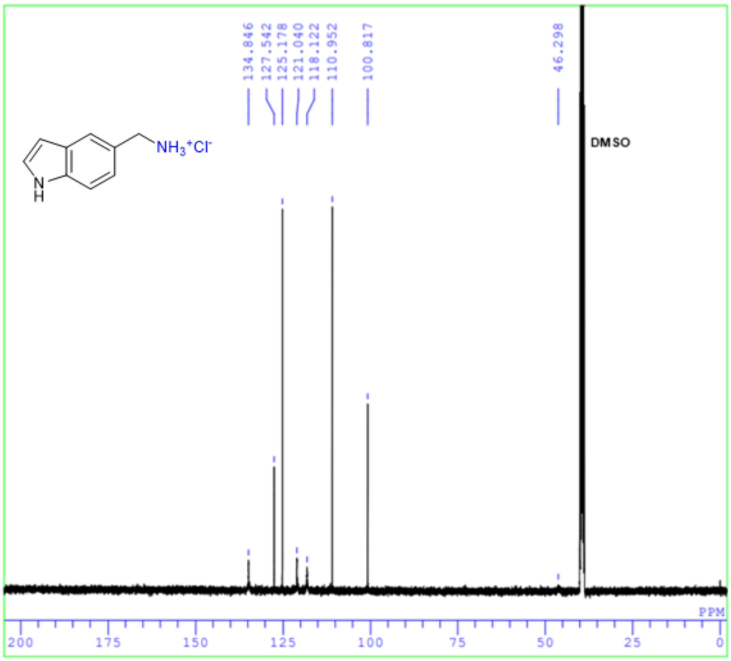

^{13}C NMR spectrum of 5-aminomethylindole (**2aa**)

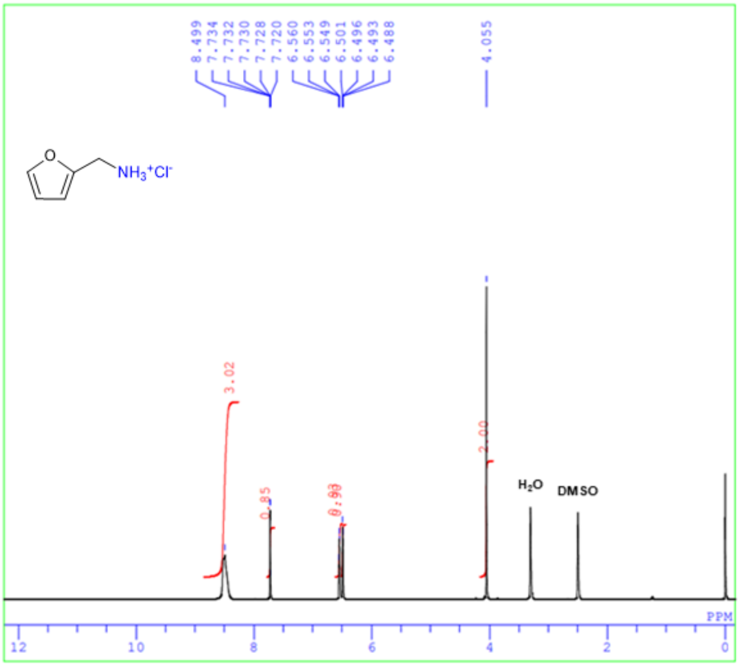

^{1}H NMR spectrum of furfurylamine hydrochloride (**2bb**)

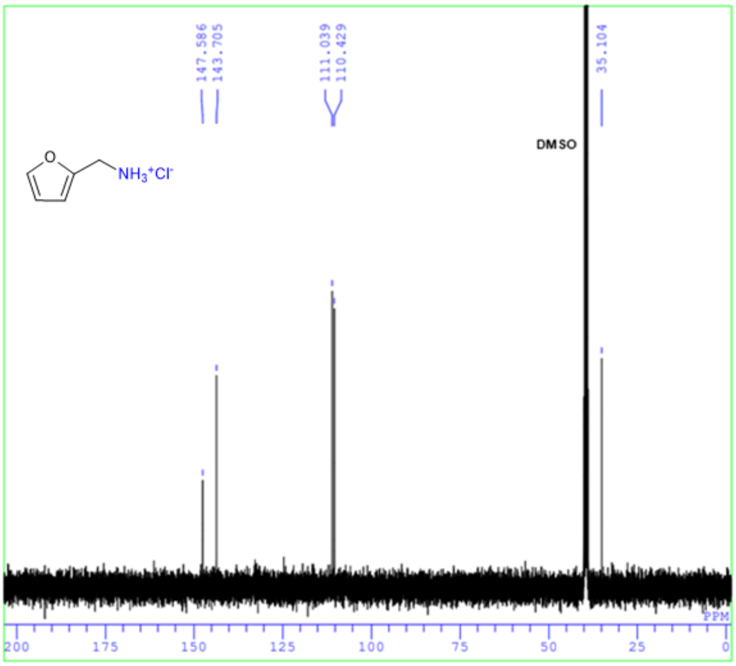

^{13}C NMR spectrum of furfurylamine hydrochloride (**2bb**)

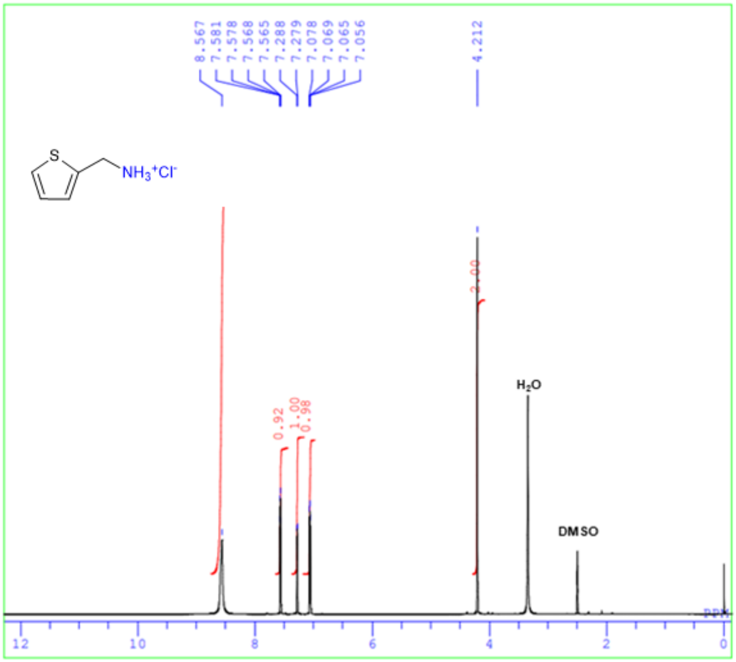

^{1}H NMR spectrum of 2-thiophenemethylamine hydrochloride (**2cc**)

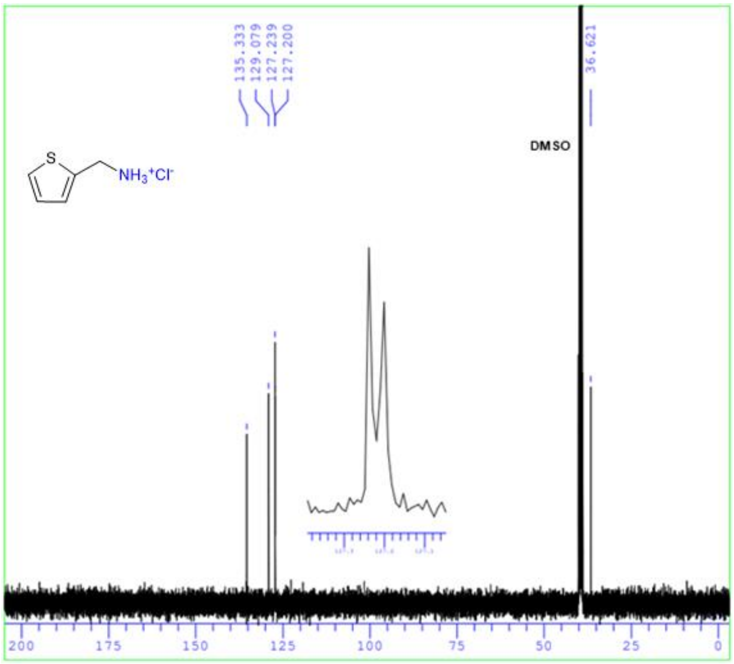

^{13}C NMR spectrum of 2-thiophenemethylamine hydrochloride (**2cc**)

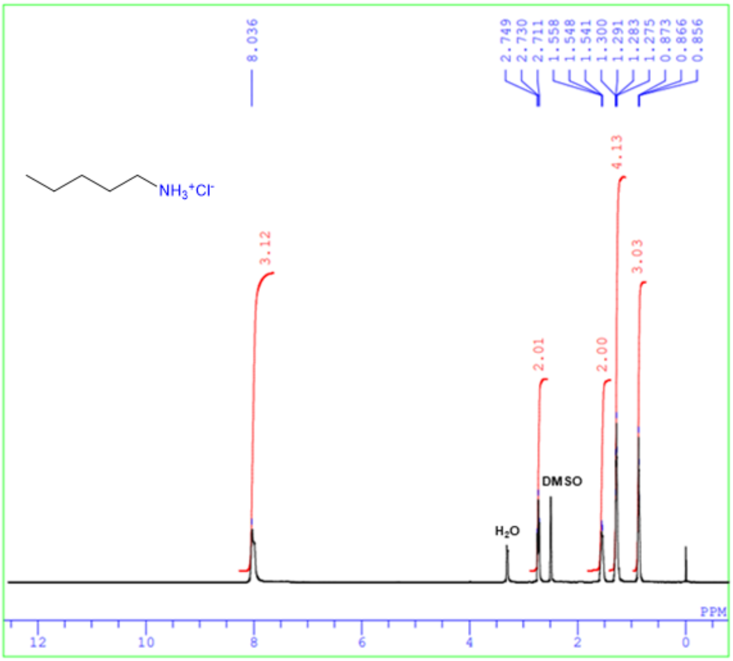

^{1}H NMR spectrum of 1-pentanamine hydrochloride (**2dd**)

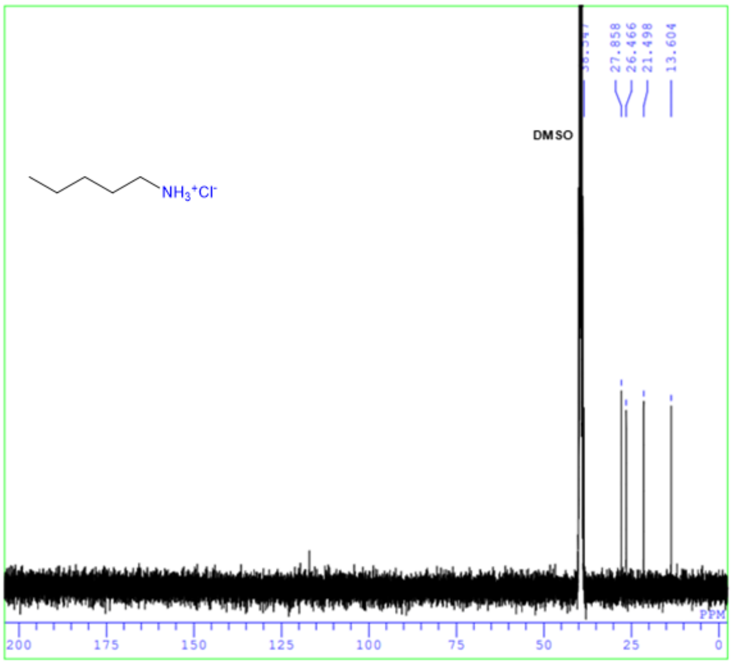

^{13}C NMR spectrum of 1-pentanamine hydrochloride (**2dd**)

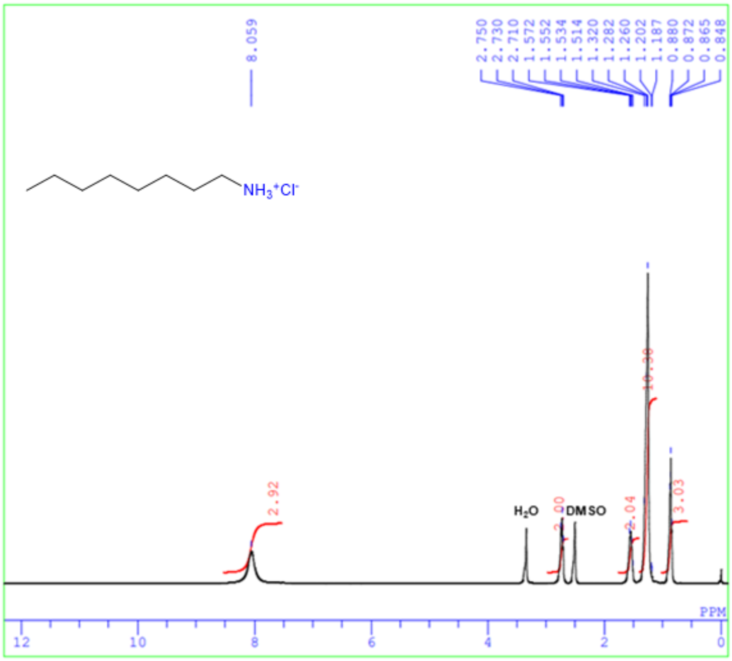

^{1}H NMR spectrum of 1-octanamine hydrochloride (**2ee**)

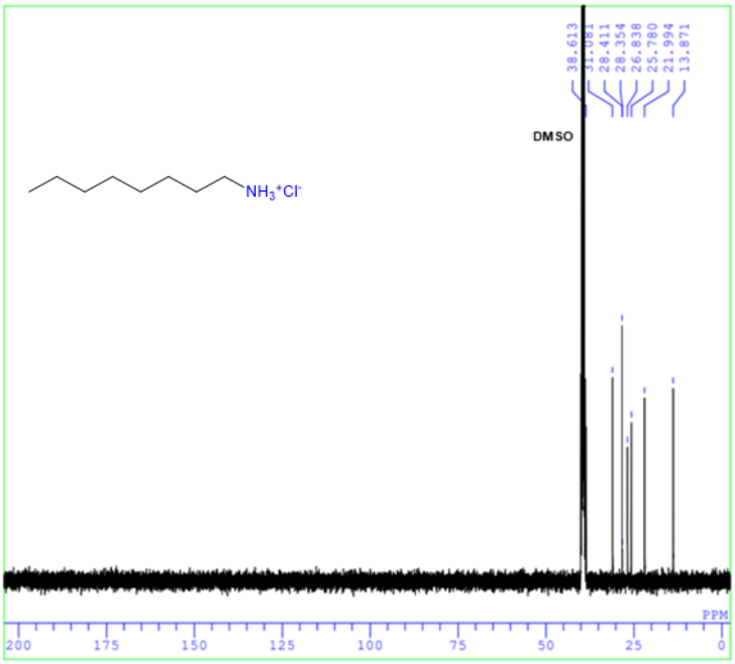

^{13}C NMR spectrum of 1-octanamine hydrochloride (**2ee**)

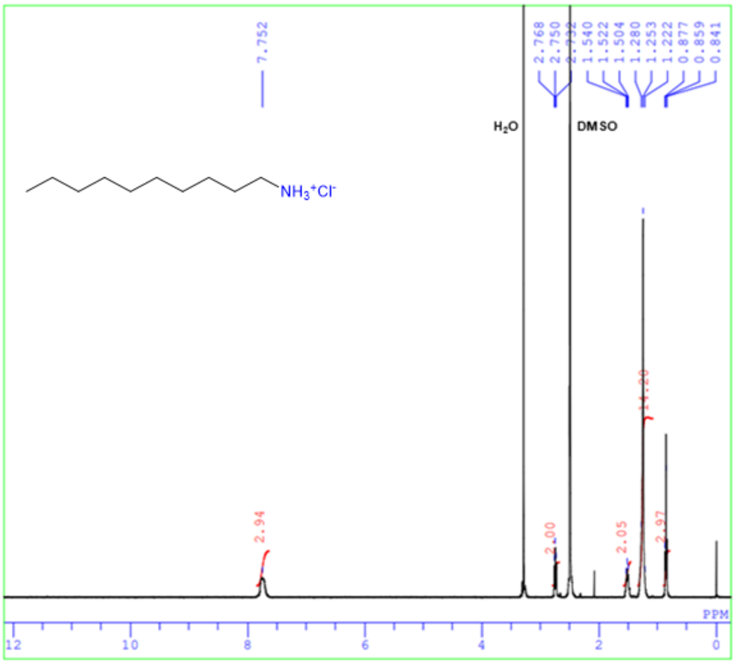

^{1}H NMR spectrum of 1-decanamine hydrochloride (**2ff**)

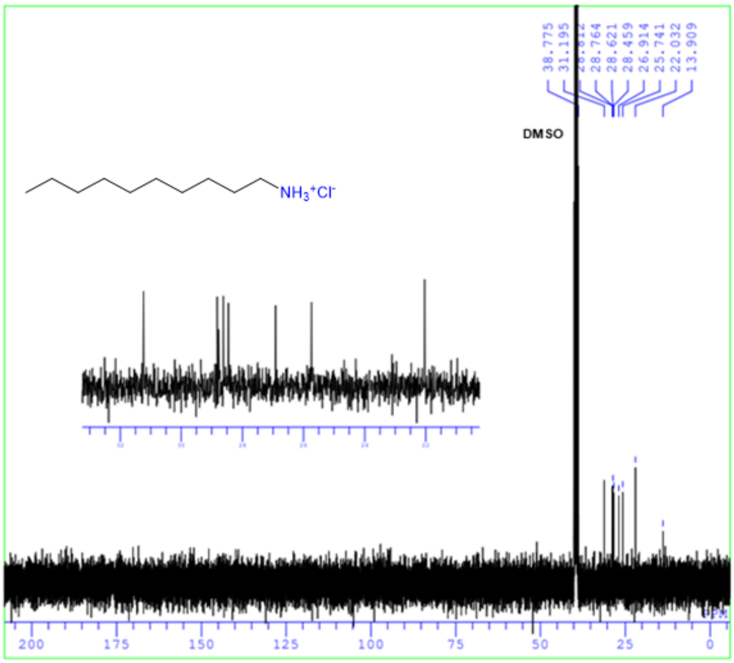

^{13}C NMR spectrum of 1-decanamine hydrochloride (**2ff**)

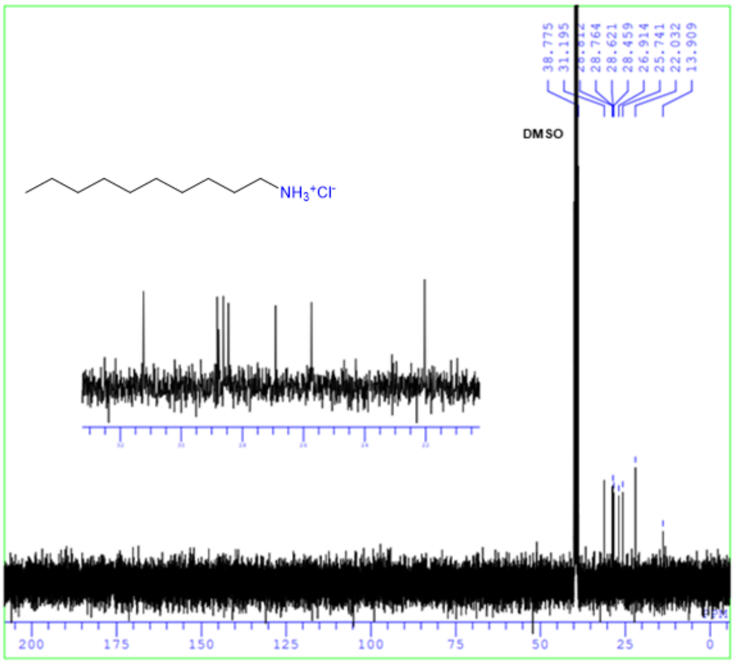

^{1}H NMR spectrum of 1-dodecanamine hydrochloride (**2gg**)

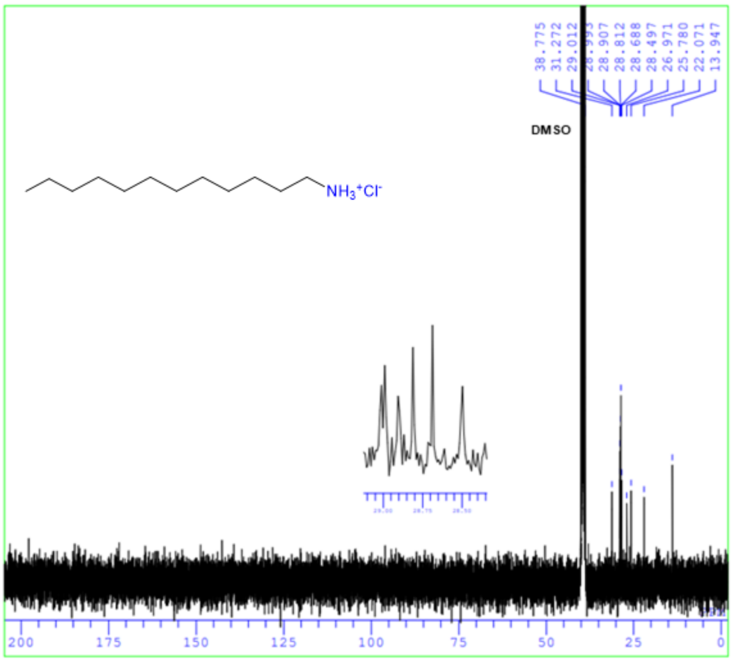

^{13}C NMR spectrum of 1-dodecanamine hydrochloride (**2gg**)

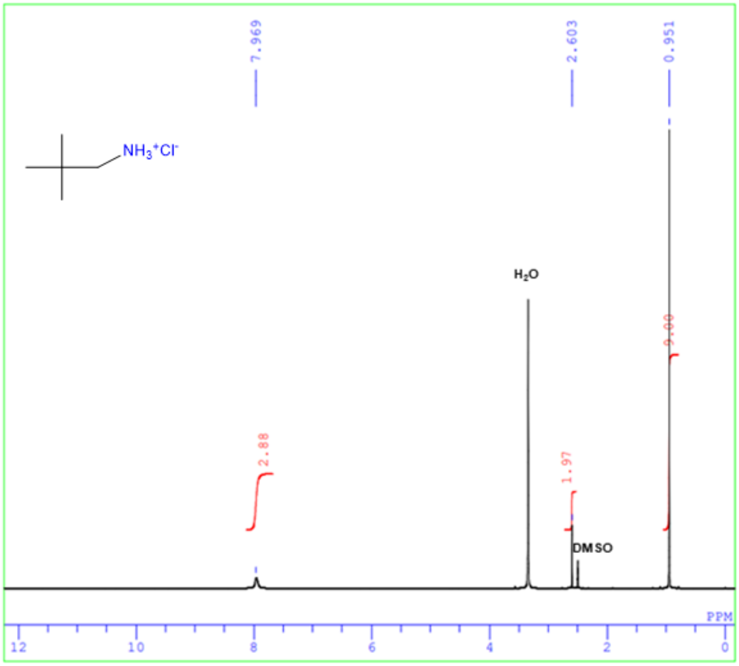

^{1}H NMR spectrum of neopentylamine hydrochloride (**2hh**)

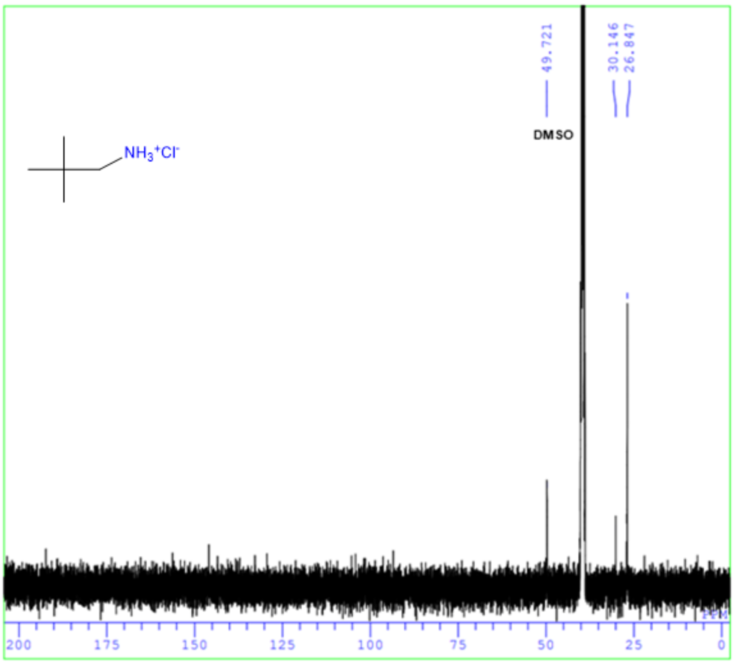

^{13}C NMR spectrum of neopentylamine hydrochloride (**2hh**)

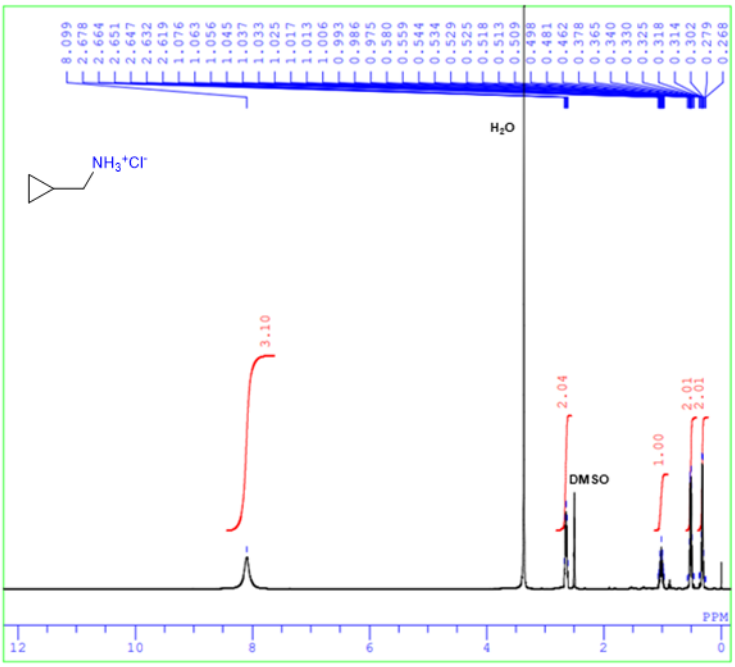

^{1}H NMR spectrum of cyclopropanemethylamine hydrochloride (**2ii**)

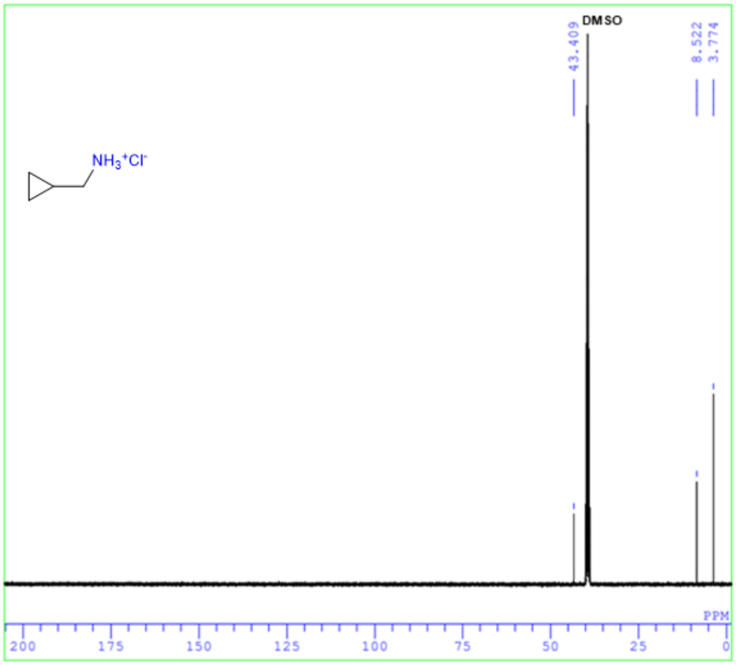

^{13}C NMR spectrum of cyclopropanemethylamine hydrochloride (**2ii**)

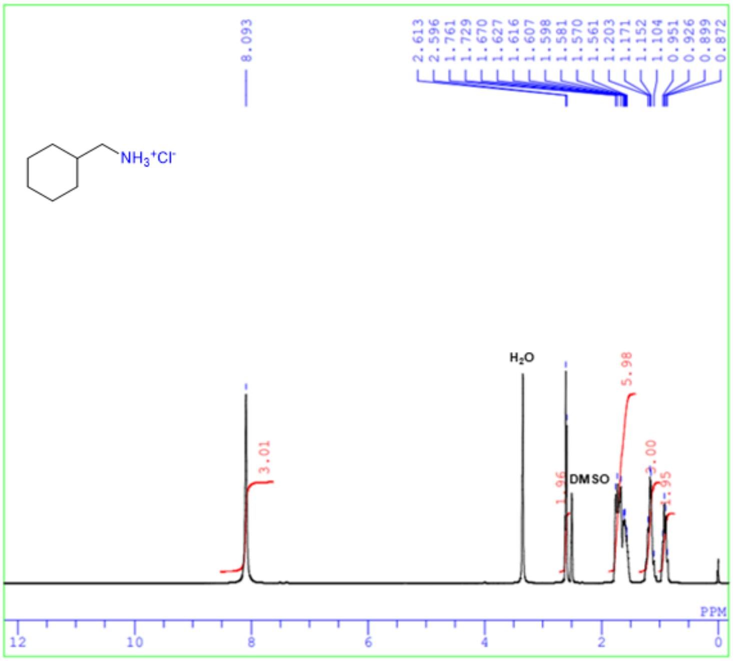

^{1}H NMR spectrum of cyclohexanemethylamine hydrochloride (**2jj**)

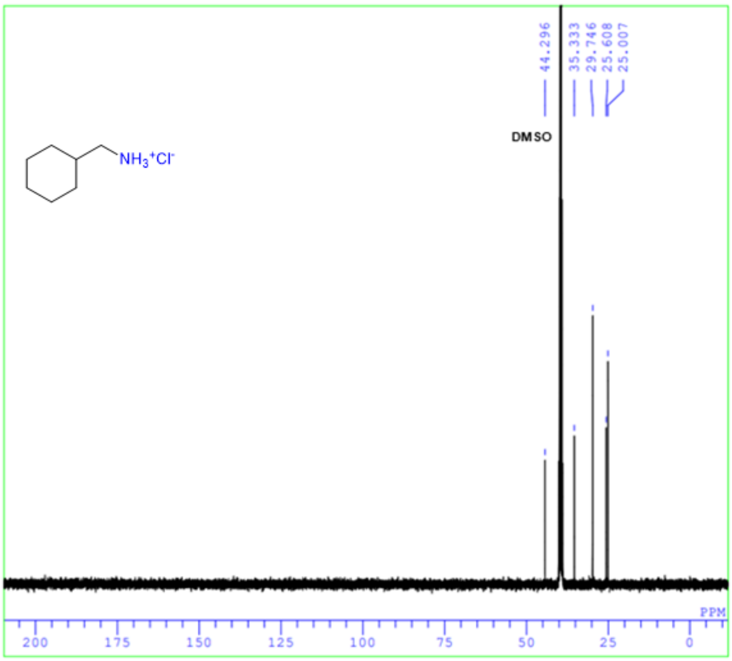

^{13}C NMR spectrum of cyclohexanemethylamine hydrochloride (**2jj**)

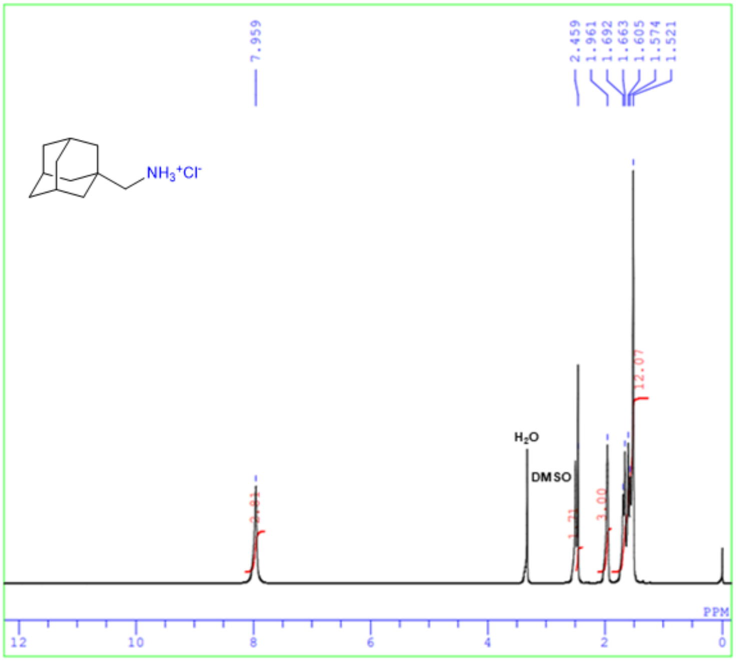

^{1}H NMR spectrum of 1-adamantanemethylamine hydrochloride (**2kk**)

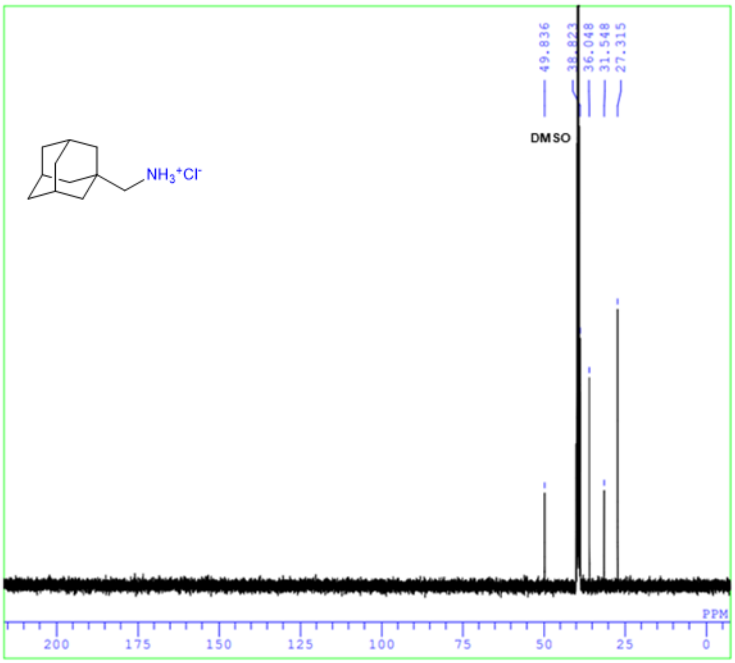

^{13}C NMR spectrum of 1-adamantanemethylamine hydrochloride (**2kk**)

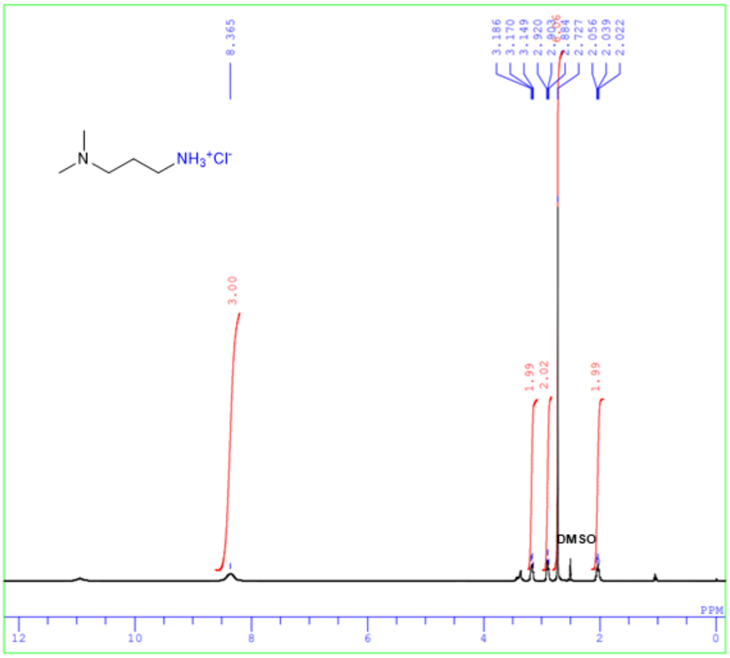

^{1}H NMR spectrum of *N,N*-dimethyl-1,3-propanediamine hydrochloride (**2ll**)

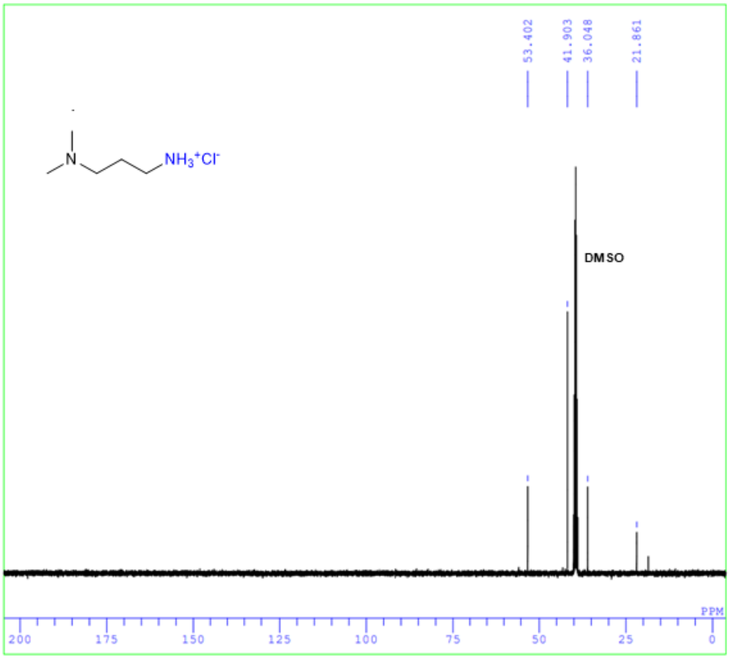

^{13}C NMR spectrum of *N,N*-dimethyl-1,3-propanediamine hydrochloride (**2ll**)

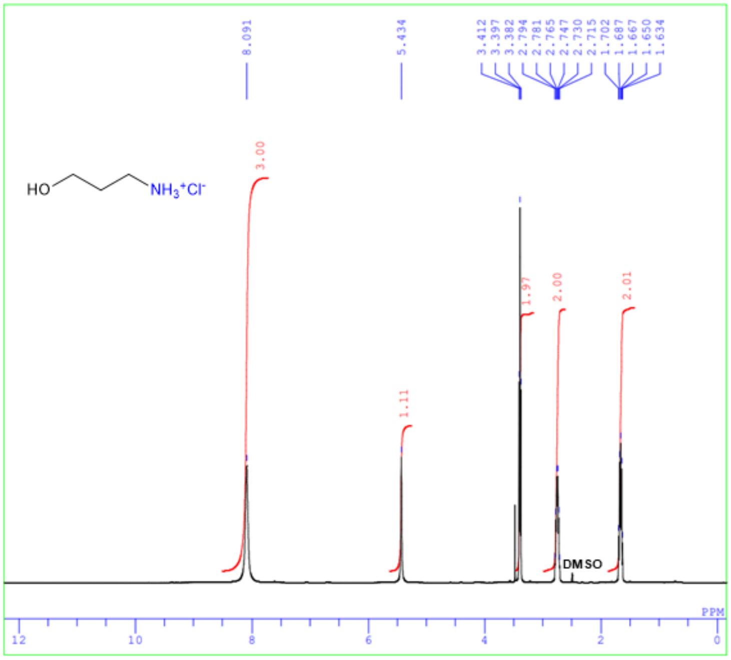

^{1}H NMR spectrum of 3-amino-1-propanol hydrochloride (**2mm**)

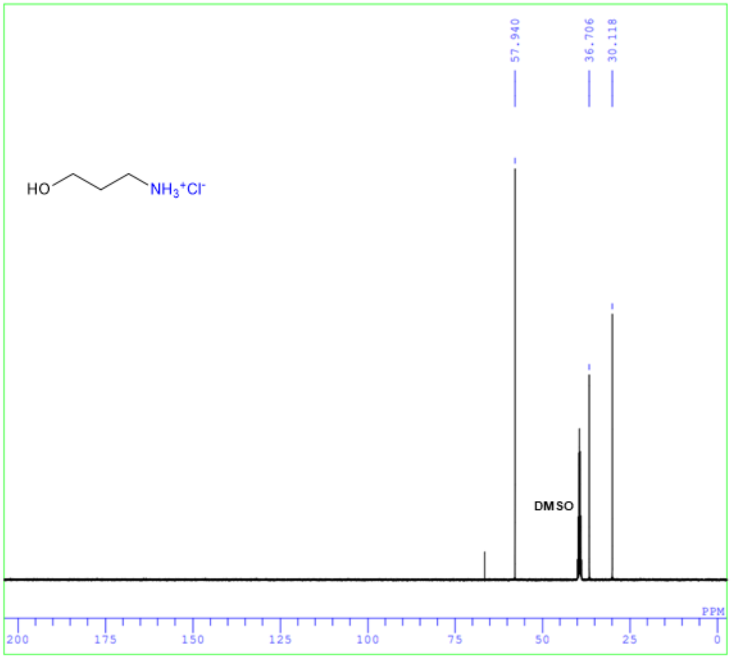

^{13}C NMR spectrum of 3-amino-1-propanol hydrochloride (**2mm**)

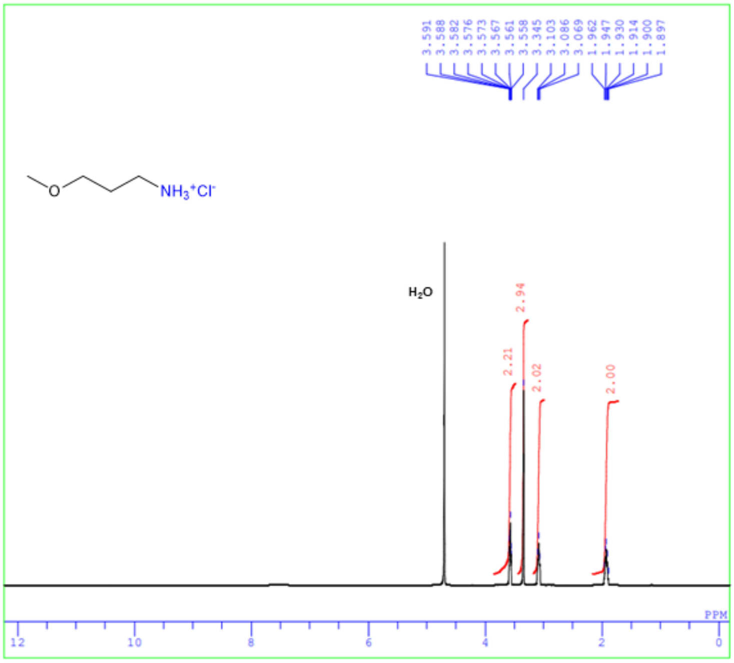

^{1}H NMR spectrum of 3-methoxypropanamine hydrochloride (**2nn**)

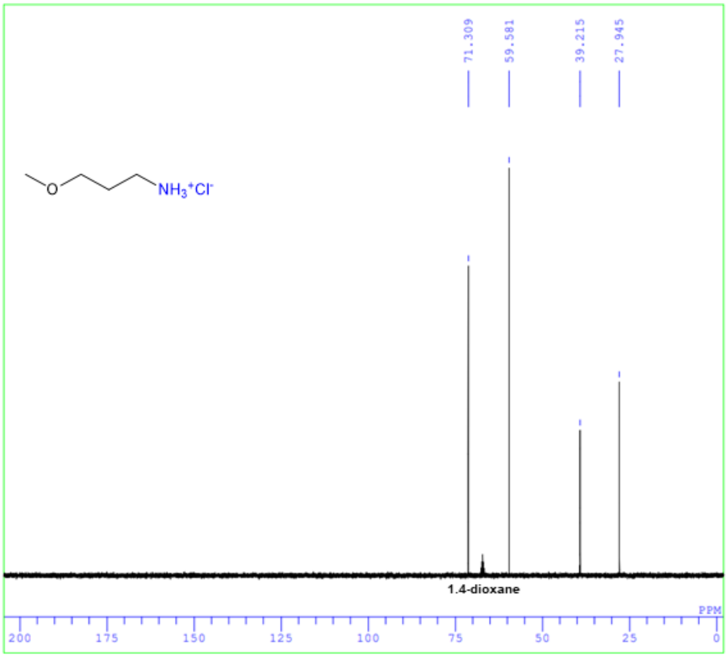

^{13}C NMR spectrum of 3-methoxypropanamine hydrochloride (**2nn**)

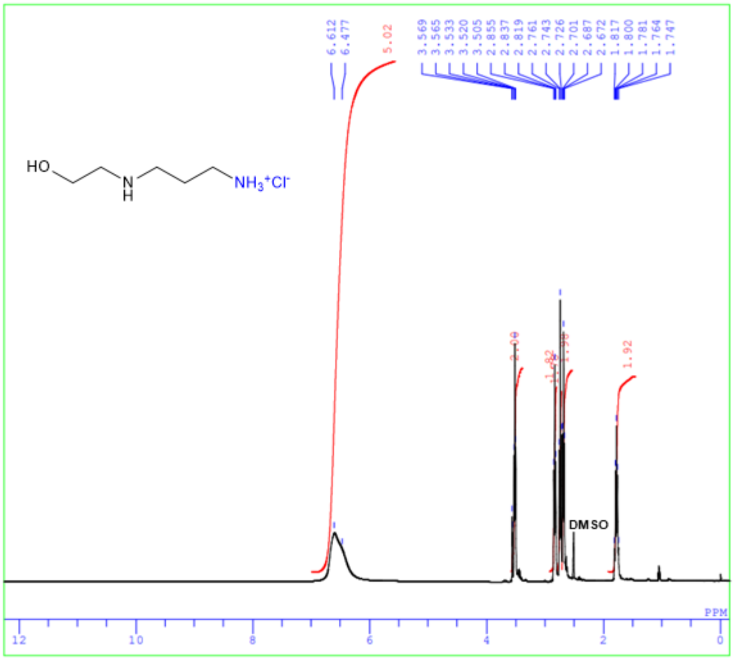

^{1}H NMR spectrum of 2-[(3-aminopropyl)amino]ethanol hydrochloride (**2oo**)

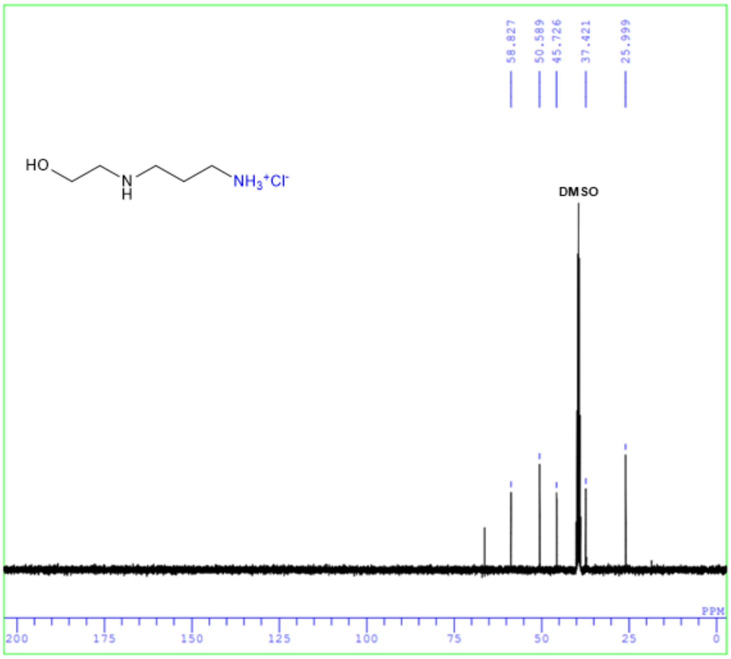

^{13}C NMR spectrum of 2-[(3-aminopropyl)amino]ethanol hydrochloride (**2oo**)

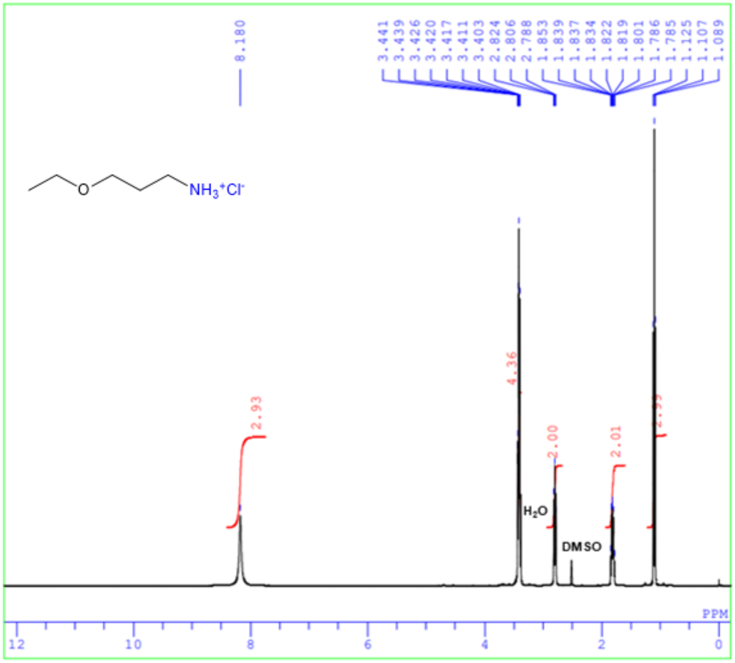

^{1}H NMR spectrum of 3-ethoxypropanamine hydrochloride (**2pp**)

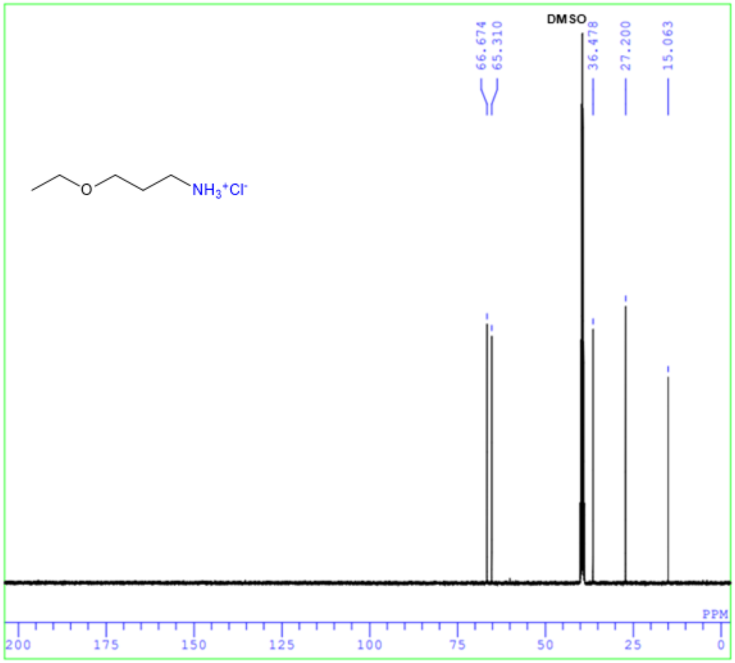

^{13}C NMR spectrum of 3-ethoxypropanamine hydrochloride (**2pp**)

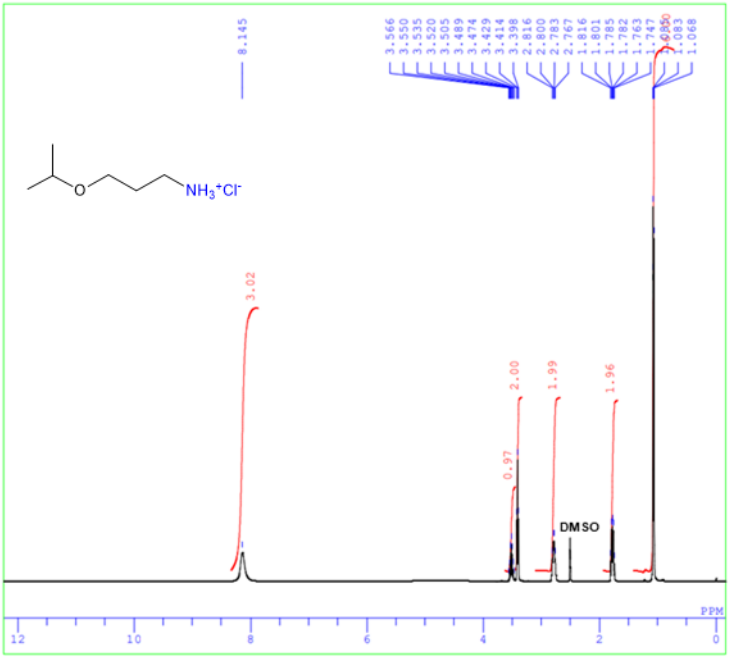

^{1}H NMR spectrum of 3-isopropoxypropanamine hydrochloride (**2qq**)

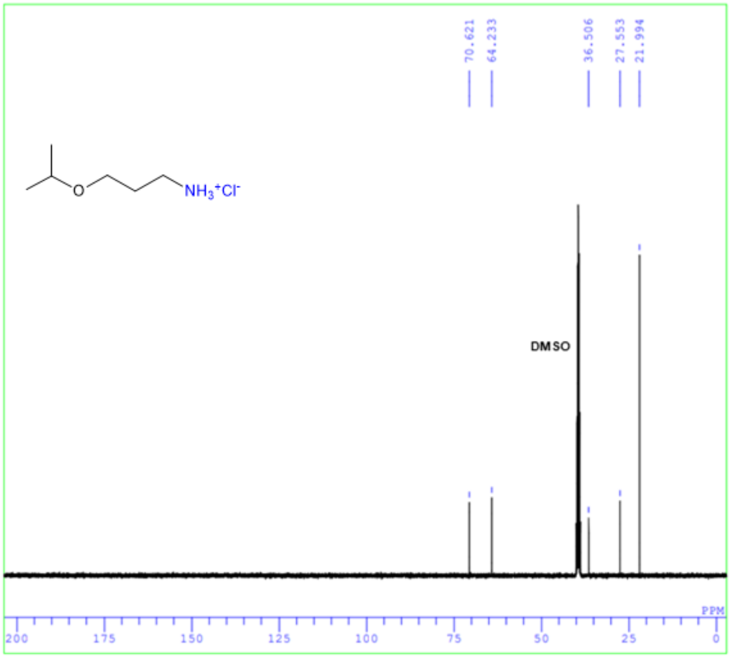

^{13}C NMR spectrum of 3-isopropoxypropanamine hydrochloride (**2qq**)

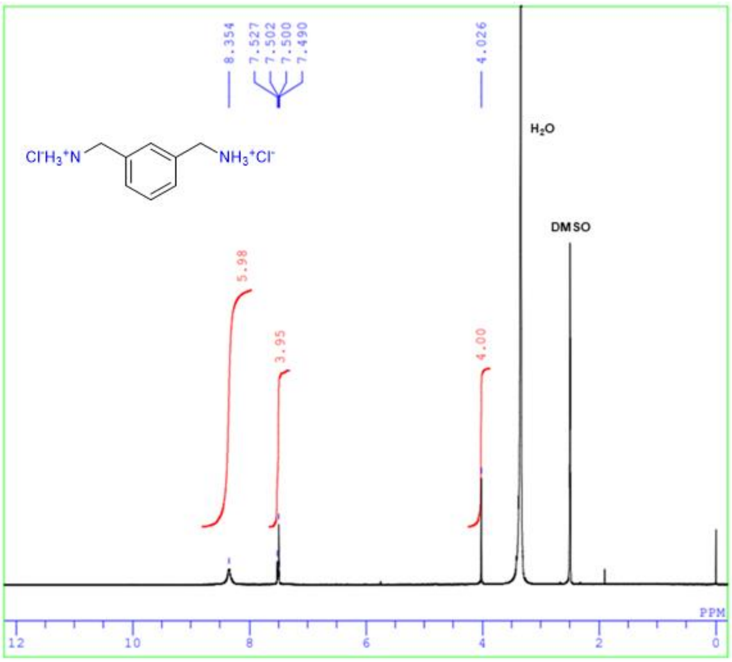

^{1}H NMR spectrum of *m*-xylylenediamine dihydrochloride (**2rr**)

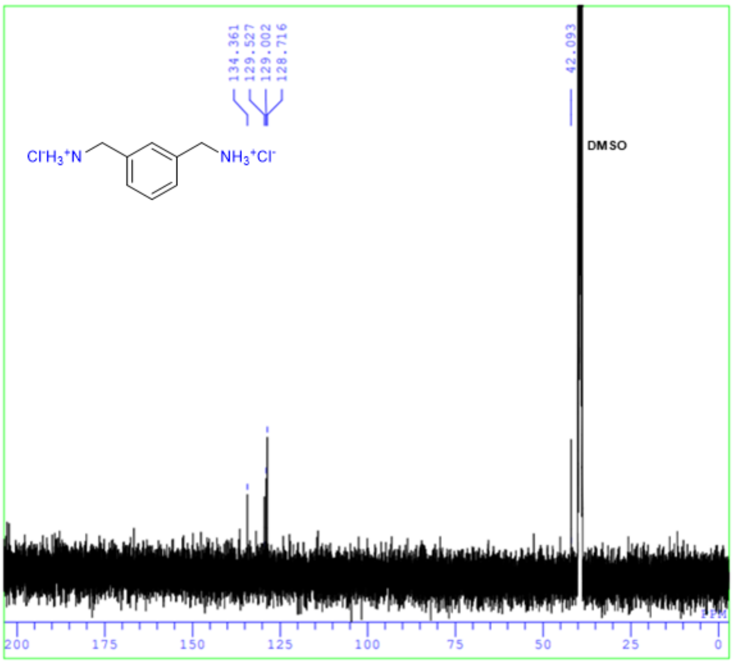

^{13}C NMR spectrum of *m*-xylylenediamine dihydrochloride (**2rr**)

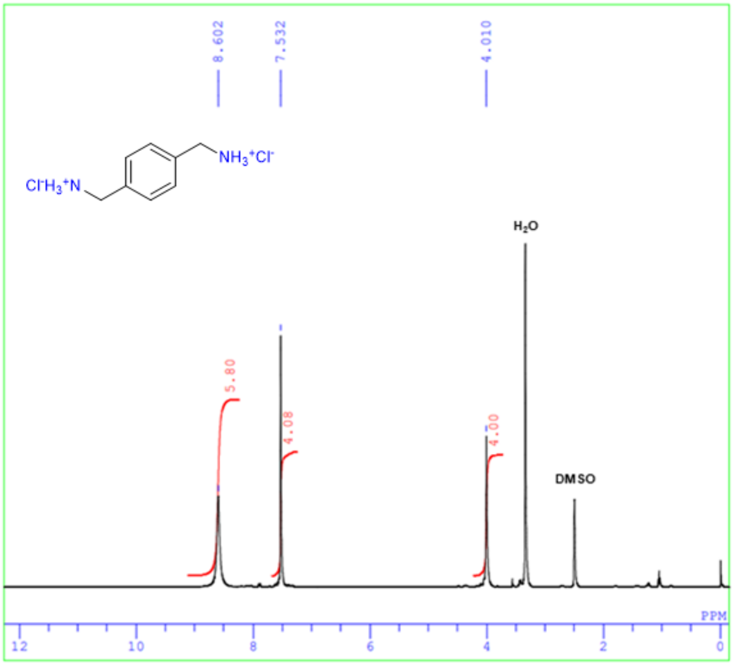

^{1}H NMR spectrum of *p*-xylylenediamine dihydrochloride (**2ss**)

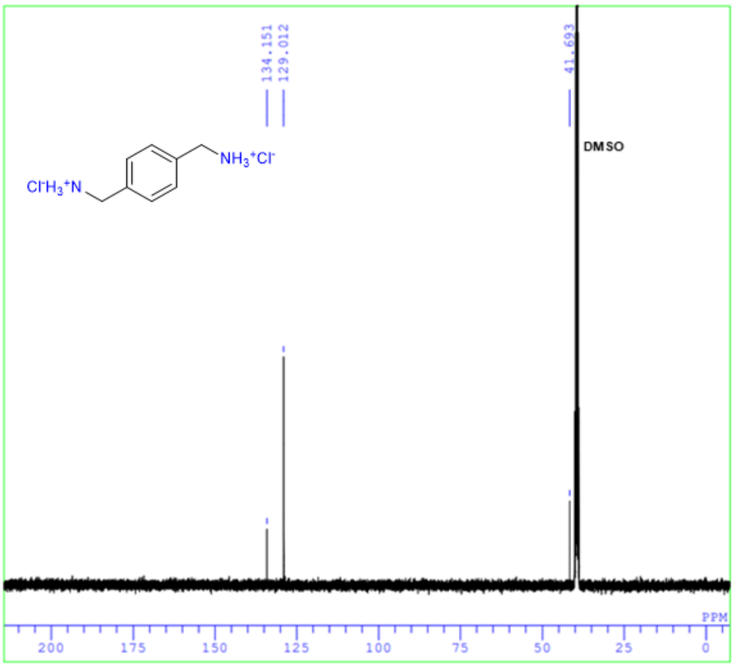

^{13}CNMR spectrum of *p*-xylylenediamine dihydrochloride (**2ss**)

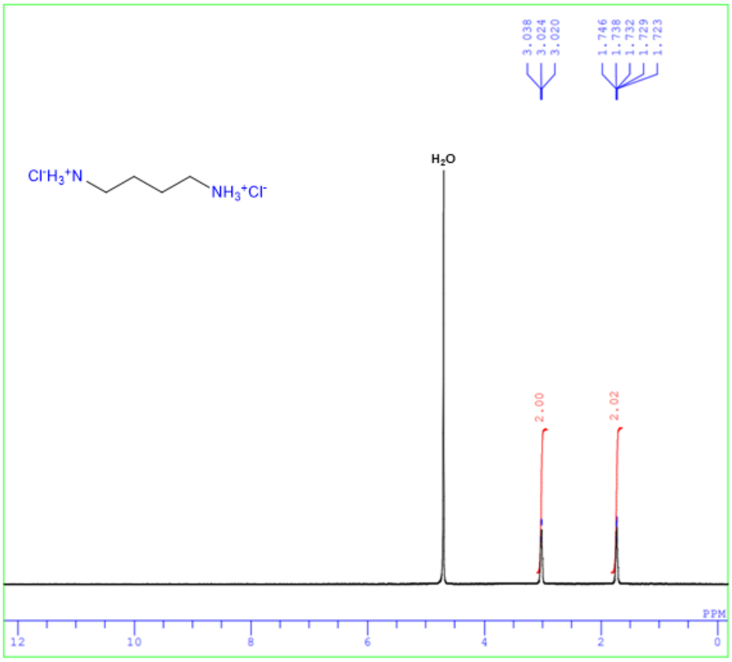

^{1}H NMR spectrum of 1,4-butanediamine dihydrochloride (**2tt**)

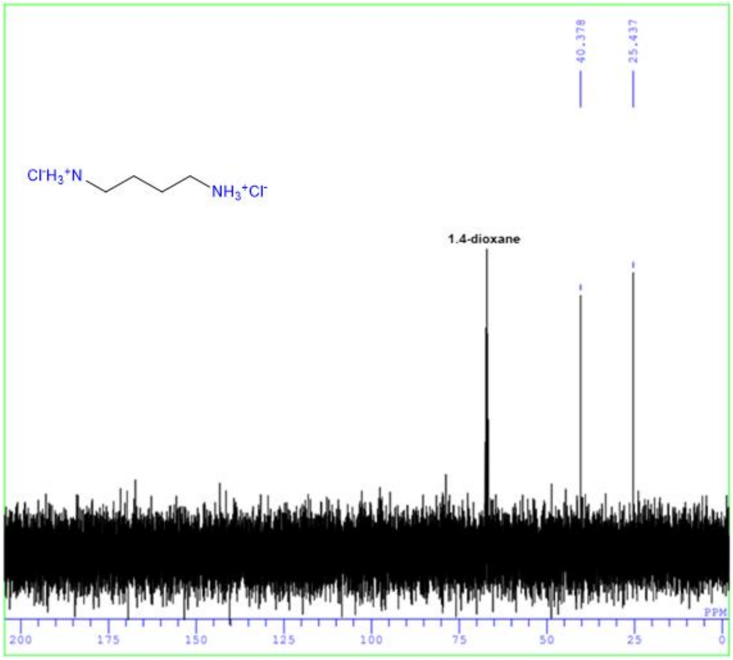

^{13}C NMR spectrum of 1,4-butanediamine dihydrochloride (**2tt**)

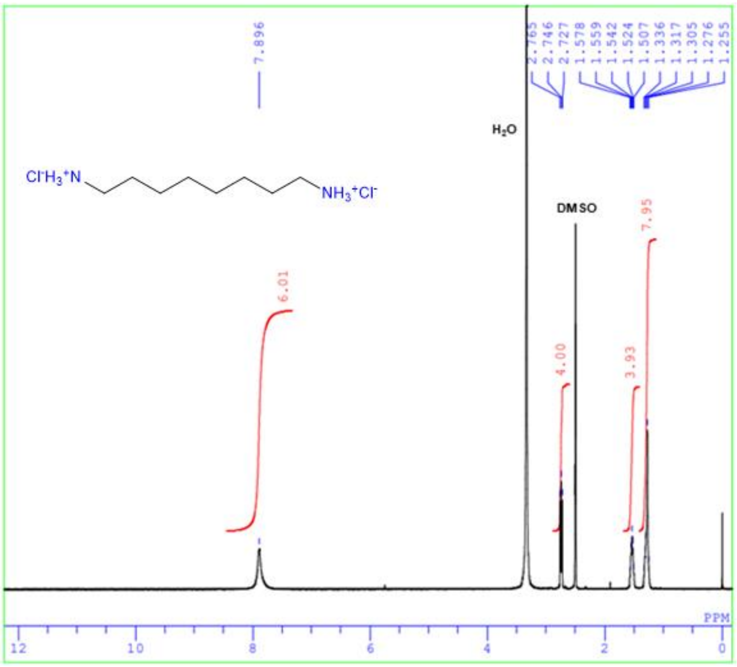

^{1}H NMR spectrum of 1,8-octanediamine dihydrochloride (**2uu**)

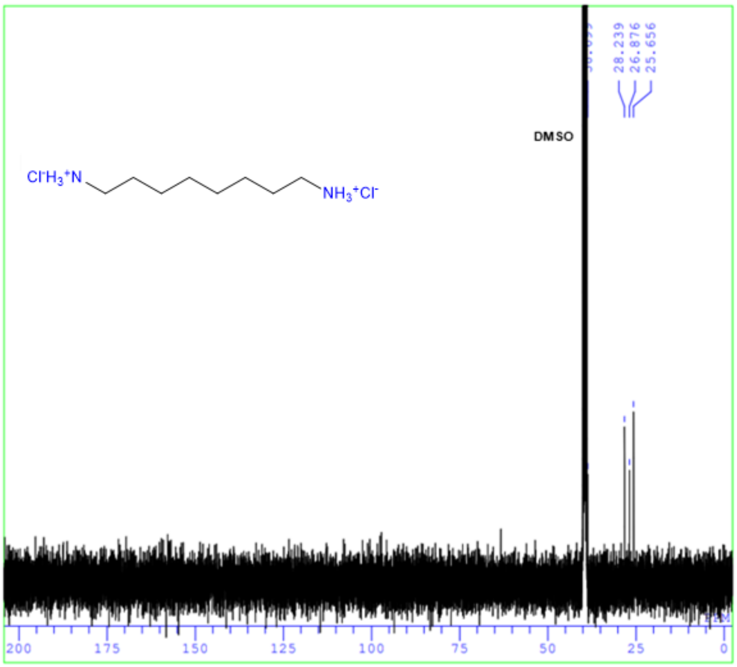

^{13}C NMR spectrum of 1,8-octanediamine dihydrochloride (**2uu**)

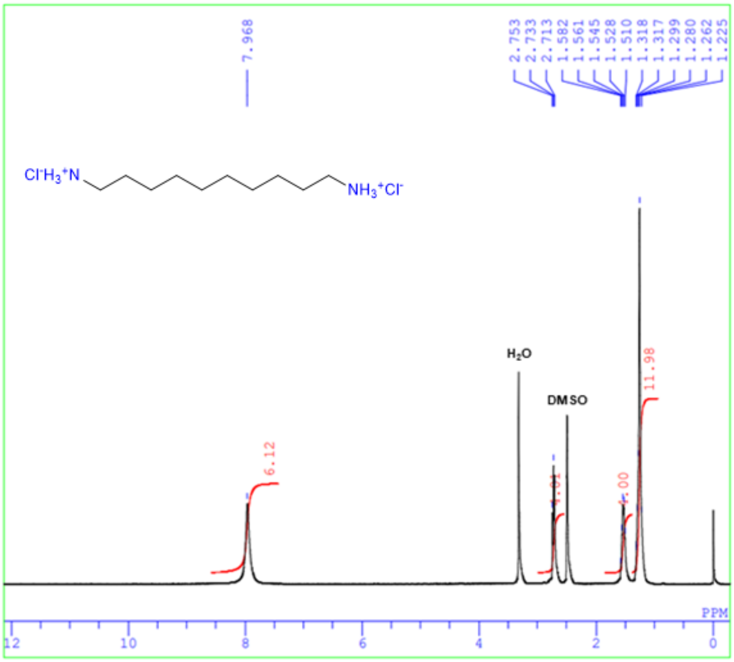

^{1}H NMR spectrum of 1,10-decanediamine dihydrochloride (**2vv**)

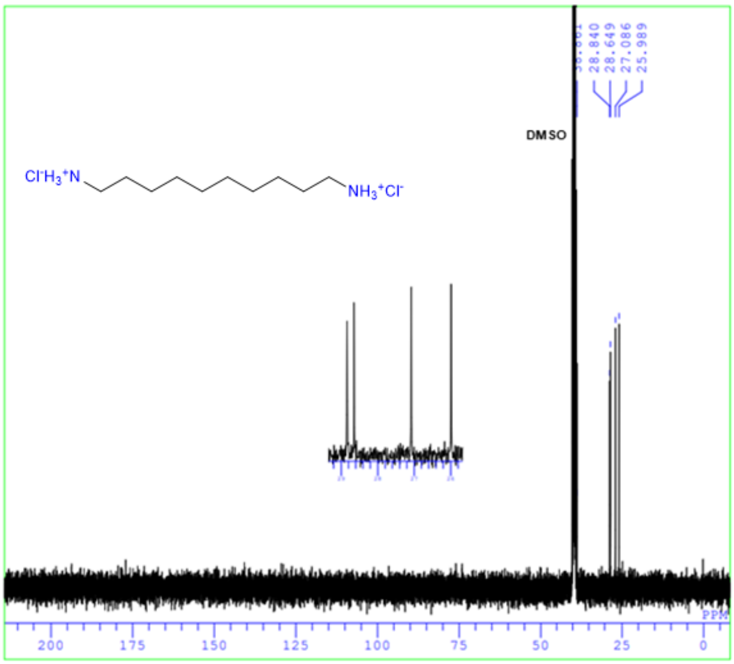

^{13}C NMR spectrum of 1,10-decanediamine dihydrochloride (**2vv**)

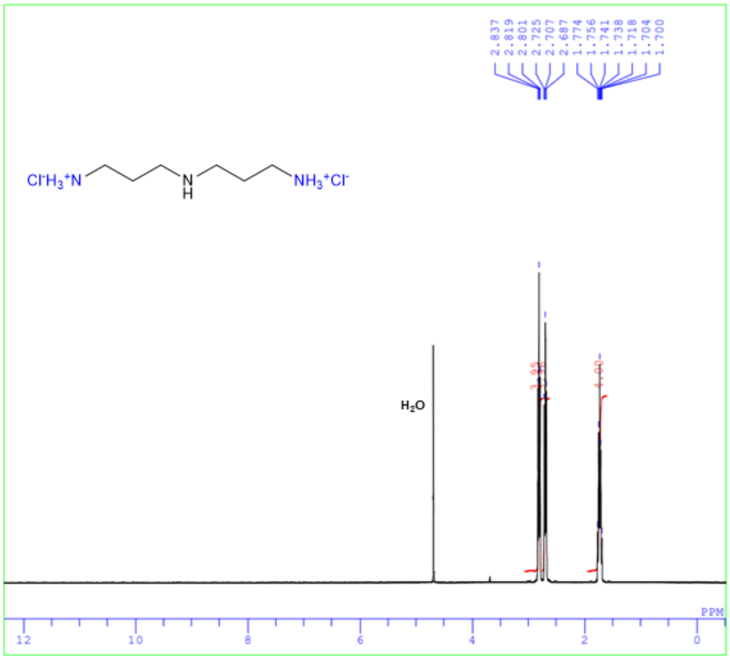

^{1}H NMR spectrum of bis(3-aminopropyl)amine dihydrochloride (**2ww**)

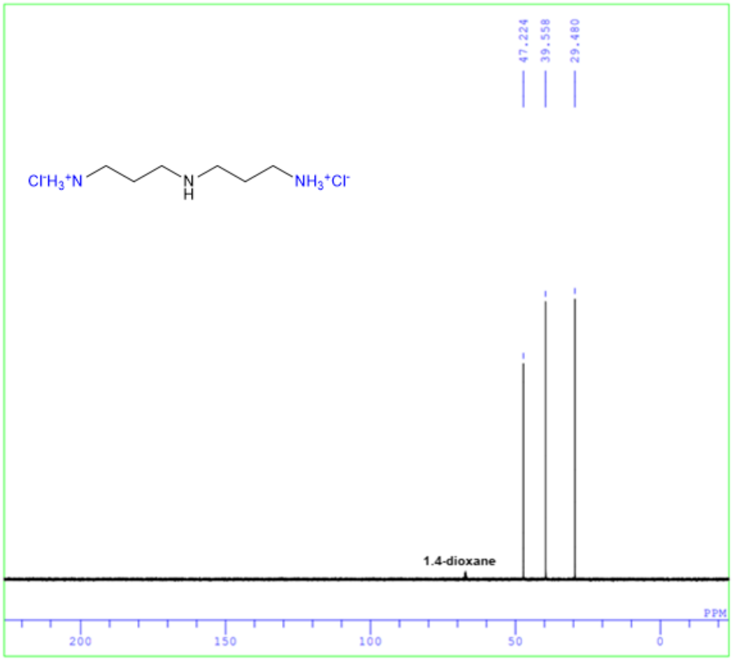

^{13}C NMR spectrum of bis(3-aminopropyl)amine dihydrochloride (**2ww**)

4. Supplementary discussion

In relation to the XPS analysis, as pointed out by one of the reviewers, the appearance of a FeO peak in the XPS spectrum of Fe_2P NC/C has raised suspicions of oxidation during the synthesis process of Fe_2P NC/support, as opposed to metal-support interaction.

We acknowledge these concerns. Generally, distinguishing whether a characteristic peak of a metal ion originates from surface oxidation or metal-support interaction through oxygen species of supports poses significant difficulty.

As depicted in Fig. 2 of the manuscript, the XPS spectra of Fe_2P NC/C, Fe_2P NC/TiO_2, and Fe_2P NC/SiO_2 show minor peaks associated with ionic Fe species (FeO). Among them, the intensity of ionic Fe species in Fe_2P NC/TiO_2 and Fe_2P NC/SiO_2 surpasses that of Fe_2P NC/C, probably due to the oxygen-rich nature of TiO_2 and SiO_2 surfaces in comparison to carbon. This discrepancy suggests the formation of Fe–O–X bonds (where X = Ti, Si, or C) which are facilitated by metal-support interactions. The relatively lower intensity of the peak corresponding to Fe–O–C bonds might be attributed to interactions between Fe and residual oxygen sites on the partially oxidized carbon support.

Furthermore, it's important to consider the expected negative effect of surface oxidation on the catalytic performance of Fe_2P NC. Unexpectedly, Fig. 3 demonstrates that the catalytic activity of Fe_2P NC/C exceeds that of unsupported Fe_2P NC (with product yields of 28% and 20%, respectively, in the hydrogenation of benzonitrile). This experimental result provides additional support for the interpretation that the peak attributed to ionic Fe species more likely signifies the formation of Fe–O–C bonds, rather than surface oxidation. It is acknowledged that low valent Fe species are loaded on carbon support through Fe–O–C bonding[S35,S36].

Based on these collective findings, we currently conclude that the minor peaks corresponding to ionic Fe species likely arise from the formation of Fe–O–X bonds (where X = Ti, Si, or C) facilitated by metal-support interactions.

5. Supplementary References

[S1] Chandrashekhar, V. G. et al. Silica-supported Fe/Fe–O nanoparticles for the catalytic hydrogenation of nitriles to amines in the presence of aluminium additives. *Nat. Catal.* 5, 20–29 (2022). 10.1038/s41929-021-00722-x.

[S2] Fujita, S. et al. Ni_2P nanoalloy as an air-stable and versatile hydrogenation catalyst in water: P-alloying strategy for designing smart catalysts. *Chem. Eur. J.* 27, 4439–4446 (2021). 10.1002/chem.202005037.

[S3] Zhang, Y., Yang, H., Chi, Q. & Zhang, Z. Nitrogen-doped carbon-supported nickel nanoparticles: A robust catalyst to bridge the hydrogenation of nitriles and the reductive amination of carbonyl compounds for the synthesis of primary amines. *ChemSusChem* 12, 1246–1255 (2019). 10.1002/cssc.201802459.

[S4] Konnerth, H. & Prechtl, M. H. G. Nitrile hydrogenation using nickel nanocatalysts in ionic liquids. *New J. Chem.* 41, 9594–9597 (2017). 10.1039/C7NJ02210G.

[S5] Wang, J. et al. Mild and selective hydrogenation of nitriles into primary amines over a supported Ni catalyst. *New J. Chem.* 44, 549–555 (2020). 10.1039/C9NJ05307G.

[S6] Ryabchuk, P. et al. Intermetallic nickel silicide nanocatalyst–A non-noble metal–based general hydrogenation catalyst. *Sci. Adv.* 4, eaat0761 (2018). 10.1126/sciadv.aat0761.

[S7] Cao, Y.et al. A stable nickel-based catalyst derived from layered double hydroxide for selective hydrogenation of benzonitrile. *Mol. Catal.* 475, 110452 (2019). 10.1016/j.mcat.2019.110452.

[S8] Mitsudome, T. et al. A cobalt phosphide catalyst for the hydrogenation of nitriles. *Chem. Sci.* 11, 6682–6689 (2020). 10.1039/D0SC00247J.

[S9] Adam, R. et al. Selective hydrogenation of nitriles to primary amines by using a cobalt phosphine catalyst. *ChemSusChem* 10, 842–846 (2017). 10.1002/cssc.201601843.

[S10] Ji, P. et al. Single-site cobalt catalysts at new $Zr_{12}(\mu_3\text{-O})_8(\mu_3\text{-OH})_8(\mu_2\text{-OH})_6$ metal–organic framework nodes for highly active hydrogenation of nitroarenes, nitriles, and isocyanides. *J. Am. Chem. Soc.* 139, 7004−7011 (2017). 10.1021/jacs.7b02394.

[S11] Chen, F. et al. Stable and inert cobalt catalysts for highly selective and practical hydrogenation of C≡N and C=O bonds. *J. Am. Chem. Soc.* 138, 8781–8788 (2016). 10.1021/jacs.6b03439.

[S12] Murugesan, K. et al. Cobalt-based nanoparticles prepared from MOF–carbon templates as efficient

hydrogenation catalysts. *Chem. Sci.* 9, 8553–8560 (2018). 10.1039/C8SC02807A.

[S13] Ferraccioli, R. et al. Synthesis of cobalt nanoparticles by pyrolysis of vitamin B_{12}: A non-noble-metal catalyst for efficient hydrogenation of nitriles. *Catal. Sci. Technol.* 8, 499–507 (2018). 10.1039/C7CY01577A.

[S14] Formenti, D. et al. A state-of-the-art heterogeneous catalyst for efficient and general nitrile hydrogenation. *Chem. Eur. J.* 26, 15589–15595 (2020). 10.1002/chem.202001866.

[S15] Sheng, M. et al. Hydrotalcite-supported cobalt phosphide nanorods as a highly active and reusable heterogeneous catalyst for ammonia-free selective hydrogenation of nitriles to primary amines. *ACS Sustain. Chem. Eng.* 9, 11238–11246 (2021). 10.1021/acssuschemeng.1c03667.

[S16] Sanagawa, A. & Nagashima, H. Hydrosilane reduction of nitriles to primary amines by cobalt-isocyanide catalysts. *Org. Lett.* 21, 287–291 (2019). 10.1021/acs.orglett.8b03736.

[S17] Gautam, N. et al. Bicyclic (alkyl)(amino)carbene (BICAAC) as a metal-free catalyst for reduction of nitriles to amines. *Chem. Commum.* 58, 3047–3050 (2022). 10.1039/D1CC06962D.

[S18] Pandey, V. K., Tiwari, C. S. & Rit, A. Silver-catalyzed hydroboration of C–X (X = C, O, N) multiple bonds. *Org. Lett.* 23, 1681–1686 (2021). 10.1021/acs.orglett.1c00106.

[S19] Elangovan, S. et al. Selective catalytic hydrogenations of nitriles, ketones, and aldehydes by well-defined manganese pincer complexes. *J. Am. Chem. Soc.* 138, 8809–8814 (2016). 10.1021/jacs.6b03709.

[S20] Bisai, M. K., Gour, K., Das, T., Vanka, K. & Sen, S. S. Lithium compound catalyzed deoxygenative hydroboration of primary, secondary and tertiary amides. *Dalton Trans.* 50, 2354–2358 (2021). 10.1039/D1DT00364J.

[S21] Yu, J. H. et al. Fabrication of ω-transaminase@metal-organic framework biocomposites for efficiently synthesizing benzylamines and pyridylmethylamines. *Adv. Synth. Catal.* 364, 380–390 (2022). 10.1002/adsc.202100997.

[S22] Reeves, J. T. et al. A practical procedure for reduction of primary, secondary and tertiary amides to amines. *Adv. Synth. Catal.* 355, 47–52 (2013). 10.1002/adsc.201200835.

[S23] Pandey, P. & Bera, J. K. Hydrosilylative reduction of primary amides to primary amines catalyzed by a terminal [Ni–OH] complex. *Chem. Commun.* 57, 9204–9207 (2021). 10.1039/D1CC03537A.

[S24] Wang, Y. et al. Generalized chemoselective transfer hydrogenation/hydrodeuteration. *Adv. Synth. Catal.* 362, 4119–4129 (2020). 10.1002/adsc.202000759.

[S25] Wübbolt, S. & Oestreich, M. Exhaustive chemoselective reduction of nitriles by catalytic hydrosilylation involving cooperative Si–H bond activation. *Synlett* 28, 2411–2414 (2017). 10.1055/s-0036-1588441.

[S26] Audubert, C., Bouchard, A., Mathieu, G. & Lebel, H. Chemoselective synthesis of amines from ammonium hydroxide and hydroxylamine in continuous flow. *J. Org. Chem.* 83, 14203–14209 (2018). 10.1021/acs.joc.8b02387.

[S27] Das, H. S. et al. Primary amides to amines or nitriles: A dual role by a single catalyst. *Chem. Commun.* 55, 11868–11871 (2019). 10.1039/C9CC05856G.

[S28] Yuan, H. & Silverman, R. B. Substrates and inhibitors of gamma-aminobutyric acid aminotransferase containing bioisosteres of the carboxylic acid group: Design, synthesis, and biological activity. *Bioorg. Med. Chem.* 14, 1331–1338 (2006). 10.1016/j.bmc.2005.09.067.

[S29] Utsumi, T., Noda, K., Kawauchi, D., Ueda, H. & Tokuyama, H. Nitrile synthesis by aerobic oxidation of primary amines and in situ generated imines from aldehydes and ammonium salt with Grubbs catalyst. *Adv. Synth. Catal.* 362, 3583–3588 (2020). 10.1002/adsc.202000663.

[S30] Tokmic, K., Jackson, B. J., Salazar, A., Woods, T. J. & Fout, A. R. Cobalt-catalyzed and Lewis acid-assisted nitrile hydrogenation to primary amines: A combined effort. *J. Am. Chem. Soc.* 139, 13554–13561 (2017). 10.1021/jacs.7b07368.

[S31] Neumann, J., Bornschein, C., Jiao, H. J., Junge, K. & Beller, M. Hydrogenation of aliphatic and aromatic nitriles using a defined ruthenium PNP pincer catalyst. *Eur. J. Org. Chem.* 2015, 5944–5948 (2015). 10.1002/ejoc.201501007.

[S32] Yao, W. B. et al. A BEt_3-base catalyst for amide reduction with silane. *J. Org. Chem.* 84, 6084–6093 (2019). 10.1021/acs.joc.9b00277.

[S33] Zhao, L. et al. Cyclic (alkyl)(amino)carbene ligand-promoted nitro deoxygenative hydroboration with chromium catalysis: Scope, mechanism, and applications. *J. Am. Chem. Soc.* 143, 1618–1629 (2021). 10.1021/jacs.0c12318.

[S34] Heilmann, M. & Tiefenbacher, K. A modular phosphorylated glycoluril-derived molecular tweezer for

potent binding of aliphatic diamines. *Chem. Eur. J.* 25, 12900–12904 (2019). 10.1002/chem.201902556.

[S35] Hussain, I. et al. Insights into the mechanism of persulfate activation with nZVI/BC nanocomposite for the degradation of nonylphenol. *Chem. Eng. J.* 311, 163–172 (2017). 10.1016/j.cej.2016.11.085.

[S36] Zhang, R. et al. Remediation and optimisation of petroleum hydrocarbon degradation in contaminated water by persulfate activated with bagasse biochar-supported nanoscale zerovalent iron. *Sustainability* 14, 9324 (2022). 10.3390/su14159324.
